# Brazilian Society for Angiology and Vascular Surgery guidelines on abdominal aortic aneurysm

**DOI:** 10.1590/1677-5449.202300402

**Published:** 2023-10-30

**Authors:** Grace Carvajal Mulatti, Edwaldo Edner Joviliano, Adamastor Humberto Pereira, Alexandre Fioranelli, Alexandre Araújo Pereira, André Brito-Queiroz, Arno Von Ristow, Lucas Marcelo Dias Freire, Marcelo Martins da Volta Ferreira, Marco Lourenço, Nelson De Luccia, Pierre Galvagni Silveira, Ricardo de Alvarenga Yoshida, Ronald José Ribeiro Fidelis, Sharbel Mahfuz Boustany, Walter Junior Boim de Araujo, Julio Cesar Peclat de Oliveira

**Affiliations:** 1 Universidade de São Paulo - USP, Faculdade de Medicina, Hospital das Clínicas, São Paulo, SP, Brasil.; 2 Universidade de São Paulo - USP, Faculdade de Medicina de Ribeirão Preto - FMRP, Ribeirão Preto, SP, Brasil.; 3 Universidade Federal do Rio Grande do Sul - UFRGS, Hospital de Clínicas de Porto Alegre - HCPA, Porto Alegre, RS, Brasil.; 4 Santa Casa de São Paulo, Faculdade de Medicina, São Paulo, SP, Brasil.; 5 Universidade Federal da Bahia - UFBA, Hospital Ana Nery, Salvador, BA, Brasil.; 6 Pontifícia Universidade Católica do Rio de Janeiro - PUC-Rio, Rio de Janeiro, RJ, Brasil.; 7 Universidade Estadual de Campinas - UNICAMP, Hospital das Clínicas - HC, Campinas, SP, Brasil.; 8 Serviço Integrado de Técnicas Endovasculares - SITE, Rio de Janeiro, RJ, Brasil.; 9 Santa Casa de Curitiba, Curitiba, PR, Brasil.; 10 Universidade de São Paulo - USP, Faculdade de Medicina, São Paulo, SP, Brasil.; 11 Universidade Federal de Santa Catarina - UFSC, Florianópolis, SC, Brasil.; 12 Universidade Estadual Paulista “Júlio de Mesquita Filho” - UNESP, Faculdade de Medicina de Botucatu, Botucatu, SP, Brasil.; 13 Universidade Federal da Bahia - UFBA, Salvador, BA, Brasil.; 14 Universidade Federal do Paraná - UFPR, Hospital de Clínicas, Curitiba, PR, Brasil.; 15 Universidade Federal do Estado do Rio de Janeiro - UNIRIO, Rio de Janeiro, RJ, Brasil.

**Keywords:** aortic aneurysm, aorta, aortic disease, endovascular procedures

## Abstract

The Brazilian Society of Angiology and Vascular Surgery, through the Guidelines Project, presents new Abdominal Aortic Aneurysm Guidelines, on the subject of care for abdominal aortic aneurysm patients. Its development prioritized descriptive guidelines, using the EMBASE, LILACS, and PubMed databases. References include randomized controlled trials, systematic reviews, meta-analyses, and cohort studies. Quality of evidence was evaluated by a pair of coordinators, aided by the RoB 2 Cochrane tool and the Newcastle Ottawa Scale forms. The subjects include juxtarenal aneurysms, infected aneurysms, and new therapeutic techniques, especially endovascular procedures. The current version of the guidelines include important recommendations for the primary topics involving diagnosis, treatment, and follow-up for abdominal aortic aneurysm patients, providing an objective guide for medical practice, based on scientific evidence and widely available throughout Brazil.

## INTRODUCTION

The Guidelines Project was established in 2002, by the combined efforts of the Federal Medical Council (CFM) and the Brazilian Medical Association (AMB).^1^ The Guidelines are a set of medical information about a given subject, organized by and based on high-quality scientific evidence. The intent at the time, and the project's guiding star to this day, was to provide information that could help medical professionals make diagnostic, therapeutic, and follow-up decisions for their patients.

In 2016, the Brazilian Ministry of Health published a document discussing the importance of medical guidelines and suggesting a methodology to enable the production of documents characterized by high scientific quality.^2^ The accumulated experience and the development of standard procedure protocols seem to have a direct influence on improved results, lowering morbidity and mortality for patients.

AMB maintains a website (http://www.projetodiretrizes.org.br/) where physicians throughout Brazil can read Guidelines divided by topic and medical specialty societies. One of the guiding principles of this project is to increase the accessibility and dissemination of the documents produced, and the AMB website makes guidelines available free of charge. The Brazilian Society of Angiology and Vascular Surgery (SBACV) is a very representative group, as expressed by the number of members of the organization—in 2022, there were 4,232 associates. Considering the Guidelines require constant updates to continuously provide specialists with information and security, this year, SBACV has updated and added new guidelines to its library. The goal is to provide a work instrument capable of assisting clinical reasoning, but also preserves physician autonomy, as described in the CFM Code of Medical Ethics.

Abdominal aortic aneurysms (AAA) are the most frequent form of aortic aneurysm. The disease increases with age, and is most prevalent among patients over 60. Smoking is one of the primary risk factors for its growth and rupture, its most feared complication.^3^ When they occur, ruptures are lethal in most cases. In Brazil, it is estimated that between 2000 and 2016, ruptures were the direct cause of 38,000 deaths, representing 55 percent of all aortic aneurysm-related mortality.^4^

### Objective

To develop new Abdominal Aortic Aneurysm Guidelines, with well-defined methods and widely disseminated in society.

## METHODS

To develop the Abdominal Aortic Aneurysm Guidelines, the SBACV Scientific Department chose a group of authors working in clinical practice with quality scientific publications on the subject. The previous Guidelines, published in 2015, were rewritten to answer new questions and discuss previously uncovered topics.^5^ The subjects include juxtarenal aneurysms, infected aneurysms, and new therapeutic techniques, especially endovascular procedures.

Prior to writing the guidelines themselves, the group gathered and took classes on how to sort and assess evidence quality with Prof. Dr. Wanderley Marques Bernardo, one of the authors of the Guidelines Project, who has vast experience and is dedicated to working with groups such as this. Descriptive guidelines presenting a synthetic recommendation were prioritized. The following reference databases were consulted in writing these guidelines: EMBASE, LILACS, and PubMed.

The EMBASE (Elsevier) database lists indexed periodicals, conference abstracts, and technical notes, among others. In Brazil, it is available free of charge through the CAPES journal portal. The PICO process (P: Population; I: Intervention; C: Comparators; O: Outcome) was developed based on that website. The list of questions or subjects was developed in advance by the group coordinator, while the authors provided input into how the writing process would be divided. Each author wrote one section, or two at the most.

The topics addressed in this document are:

1. Methods

2. Definition

3. Epidemiology and screening

4. Etiology, pathophysiology and risk factors

5. Clinical status

6. Diagnosis

7. Treatment

a. Clinical indications, regulatory aspects, choice of devices

b. Conventional open treatment

c. Endovascular treatment of infrarenal aneurysms

d. Endovascular treatment of juxtarenal aneurysms

8. Clinical follow-up

9. Postoperative complications

a. Graft infection

b. Endoleak

10. Ruptured abdominal aortic aneurysm

11. Inflammatory aneurysm

12. Aneurysms in women

Three primary articles were used as our starting point: the last version of the Brazilian Guidelines on Aortic Aneurysm (2015),^5^ the European Society for Vascular Surgery (ESVS) Clinical Practice Guidelines (2019),^6^ and the Society for Vascular Surgery practice guidelines (2018).^7^ Articles were also selected based on the following publication categories: randomized controlled trials, systematic reviews, meta-analyses, and cohort studies. Neither case series, case reports, nor experimental trials were accepted as sources of evidence.

Evidence quality was examined by a pair of coordinators chosen in advance. Randomized controlled trials were evaluated using the RoB 2 Cochrane tool.^8^ Cohort studies, systematic reviews, and meta-analyses were used to supplement the responses, while evidence quality was assessed using the New Castle Ottawa Scale.^9^

Guideline quality is variable and can be audited using specific instruments. Despite the existence of several assessment protocols, Appraisal of Guidelines for Research and Evaluation (AGREE) is the most frequently used instrument to assess guidelines.^10^ Assessment comprises six domains: scope and purpose, stakeholder involvement, rigor of development, clarity of presentation, applicability, and editorial independence. Scores above 80 percent are acceptable for guidelines. Only four abdominal aortic aneurysm guidelines achieve that score (European Society of Cardiology, Society for Vascular Surgery, European Society of Vascular Surgery, and National Institute of Health).^6,7,11,12^

Evidently, each guideline covering a given subject or pathology must take into consideration the economic situation of their region or country. Cost-benefit analyses and local surveys for each intervention are necessary, as well as assessing the characteristics of the local health system. The recommendations from one guideline may apply at some centers and not others, but can guide treatment even in suboptimal situations.

Development of this guideline was approved by Plataforma Brasil and the Research Ethics Committee under CAAE number 62177722.2.0000.0068. SBACV was registered as a sponsor of scientific research for the first time in its history.

## DEFINITION

Aortic aneurysms comprise a dilation of two standard deviations or 50 percent greater than the expected diameter for an artery in the region. This guideline discusses infrarenal aneurysms and, therefore, dilations greater than 3 cm of diameter. Diameter measurement techniques are widely discussed, but in order to consider the greatest dilation, antero-posterior and/or transversal measurements are accepted, from the external aortic wall, via abdominal ultrasonography or computed tomography.^6,7^

Definitions of terms such as hostile neck, short neck and juxtarenal are also widely discussed, possibly due to advancements in endovascular repair techniques, as well as in instructions for use (IFUs) for endografts.^13^ For standardization purposes, this guidelines classifies as juxtarenal or pararenal aneurysms those extending to a renal artery but not involving it, or with neck below 1 cm, also known as short neck or hostile neck.^6,7^

A hostile neck, in turn, has at least one of the following characteristics: infrarenal neck longer than 28 mm, infrarenal angle smaller than 60º, neck length < 1.5 cm, thrombus thickness greater than 50 percent of circumference, conical neck (tapering greater than 0,2 cm in a 1 cm infrarenal segment), bosselation (growth greater than 0.3 cm in the initial 1.5 cm infrarenal segment).^13,14^

Taking into account the anatomical treatment frontiers explored over the last decade, this guideline considered including open and endovascular repair of juxtarenal, infrarenal, and hostile neck aneurysms, following the example of recent international guidelines.^6,7^

## EPIDEMIOLOGY AND SCREENING

The current prevalence of AAA in men over the age of 65 is 1.7 percent in the Swedish population screening study (in addition, 0.5 percent had already been diagnosed),^15^ 1.3 percent in the British screening study,^16^ 3 percent in Denmark (men aged 65-74),^17^ and 5 percent in the U.S., where screening was only made available for smokers.^18^ In Brazil, there are no population-level screening data. Prevalence was four to six times higher among men than women in Brazilian and international studies,^4,19,20^ and a 2016 meta-analysis estimated a 0.7 percent prevalence rate among women over the age of 60.^19^

In developed countries, the prevalence and incidence of AAA has decreased significantly in recent decades, which can partly be attributed to lower rates of smoking.^6,15,21,22^ According to an analysis of Datasus data published in 2020, AAA mortality in Brazil increased between 2000 and 2008, followed by a decrease between 2008 and 2016.^4^ Brazil had one of the highest rates of smoking cessation between 1990 and 2015, which may partly explain the decrease.^4^

## SCREENING

There are four major population-level AAA screening studies, in the United Kingdom, Australia, and Denmark, and a smaller screening study for women in the United Kingdom,^23-27^ all for patients 65 and older. A Cochrane review^28^ of these studies assessing AAA-related mortality found an odds ratio of 0.60 (95% CI 0.47-0.78) in favor of screening. In the longest follow-up available, all-cause mortality was significantly lower in the screening group, with an odds ratio of 0,987 (95% CI 0.975-0.999, p = 0.03).^29^ The primary harm associated with screening is the number of elective procedures, which increases twofold. However, this problem is partly compensated by the reduction in emergency procedure.^16^ Due to the high mortality associated with ruptured aneurysms and low morbidity and mortality of elective treatment, the number of men in the screening needed to prevent one aneurysm-related death would be 667, and the number of aneurysms treated 1.5.^30^ With the data available at the moment, one cannot determine the optimum age for screen in cost-benefit terms. However, a standalone abdominal ultrasound is currently recommended for men over the age of 65.^6^ There is low evidence for screening women, considering the only randomized trial focusing on the issue was underpowered for a proper statistical analysis. Therefore, at the moment, population-level screening for women is not recommended.^31^

Relatively small studies found an association between peripheral arterial disease and AAA. However, the higher prevalence rates among members of this subgroup are counterbalanced by their shorter life expectancy and high surgical risk. At the moment, there is no unequivocal evidence for screen peripheral arterial disease patients.^32^ Solid evidence correlate positive family history with risk of AAA, rapid aortic growth, and higher risk of rupture. Though the subgroup has not been adequately assessed in current studies, routine screening for patients of both genders is suggested.^33,34^ Likewise, given the frequent concomitances of peripheral aneurysms (iliac, femoral, popliteal) and AAA, screening every 5 to 10 years is also recommended for this group.^35^

The ideal periodicity of subsequent examinations was not properly assessed in randomized trials, but a model developed using a 15,000-patient database^36^ suggests intervals should be stratified according to aneurysm diameter. For aneurysms between 3 and 3.9 cm, a 3 year interval between examinations is suggests, while for AAAs between 4 and 4.9 cm, annual examinations are recommended. When the aneurysm reaches 5 cm, intervals between examinations drop to 3-6 months. Though the information on the ideal management of patients with ectatic aortas (diameter < 3 cm) is limited, a new ultrasound every 5-10 years for patients with good life expectancy is reasonable.^37^ Recommendations for abdominal aortic aneurysms can be found in Table 1.

**Table 1:** Level of recommendation for abdominal aortic aneurysm screening.

**Recommendation**	**Level of evidence**
Men over the age of 65 should be screened for abdominal aortic aneurysms with an abdominal ultrasound.	IIa
Men and women with a positive family history of abdominal aortic aneurysm should be screened starting at age 50.	IIb
The use of ultrasonography to track aortic diameters greater than 3 cm is recommended, with periodicity depending on initial diameter.	I

## ETIOLOGY, PATHOPHYSIOLOGY AND RISK FACTORS

Few countries have population-wide early screening and diagnosis programs for AAA. This means knowledge about the true incidence of new cases and the prevalence of existing ones is subject to real constraints. Consequently, we have limited data available for a more precise understanding of its major risk factors. The dearth of global data causes distortions and biases interpretation when it comes to the causes and formation mechanisms of AAA. The pursuit of that data has practical effects, since countries with national diagnosis and prevention programs (such as the United Kingdom, Sweden, and Australia) have in recent years observed remarkable decreases in rates of mortality and other complications from AAA compared to the same rates for countries with similar socioeconomic status, such as Hungary, Austria, and Romania.^32,38-42^

Male gender, advanced age, low levels of low-density lipoprotein, and smoking are the risk factors historically related to AAA, and are considered criteria for screening tests.^43,44^ Likewise, studies have found a relationship between some diseases and concomitant AAA:^3,11,43,45^ hypertension, peripheral arterial disease, ischemic heart failure, prior myocardial infarction, and chronic obstructive pulmonary disease (COPD).^46,47^

“How” and especially “why” of aneurysms form are frequent questions from patients and their family members after the AAA diagnosis, directed to angiologists and vascular surgeons. These questions embody the natural drive to understand what could be done to prevent further dilations and their frightening complications. Therefore, this section of the Guidelines discusses important aspects of what is currently known about the causes (etiology), formation (pathophysiology), and risk factors of AAA.

### Etiology

#### “Hereditary or behavioral”?

Is having an AAA predetermined at birth or are environmental factors and issues outside the body more important? Is it true that “a human being is as old as their arteries” and that dilations are the inevitable consequences of genetically determined senescence, or is aortic senility with dilation a predictable and preventable disease?

Discussing the etiology of AAAs (as well as any other pathological process) necessarily means discussing genetics as well as its younger sister, epigenetics. Both try to explain why the walls of a vessel as sturdy as the aorta can weaken to the point of becoming aneurysmal. In fact, AAA cases cluster in certain families, and there is evidence for a strong genetic component to AAA risk.^48,49^ Twin studies report genetic heritability may be as high as 70 percent. The Swedish twin register reports a monozygotic twin has a 24 percent chance of having an aneurysm if the other twin has it, compared to 4.8 percent for dizygotic twins.^15,50-52^ Positive family history approximately doubles the risk of developing AAA in these studies.

The literature also contains reports that individuals with a family history of AAA are more likely to suffer ruptures and are less likely to have heart disease compared to aneurysm cases with no family history of the disease.^53,54^ This has led some specialists to consider interventions for smaller diameters than usual for cases without positive family history.

AAA inheritance may be Mendelian (single gene) or non-Mendelian, with a more complex cause stemming from various genes. Rare genetic diseases, such as Ehlers-Danlos syndrome, Marfan syndrome, Loeys-Dietz syndrome, and fibromuscular dysplasia, which may cause AAA, are examples of Mendelian inheritance. However, these diseases are rare, and epidemiological analyses find that only 10 to 20 percent of AAA patients have at least one relative who suffer from a genetic disease. This may suggest that a more complex mechanism, rather than changes to a single gene, are behind the genetic causes of AAA. Further evidence suggests epigenetic mechanisms (environmental and behavioral risk factors) play an important role in vascular disease and the smooth muscle cell plasticity in the vascular system associated with the process.^55^

Epigenetics refers to hereditary and acquired changes to the genome affecting gene expression without changing DNA sequences. In some cases, epigenetic changes are stable and passed down across generations, but many are relatively dynamic and respond to environmental cues.^56^ Epigenetic changes include DNA methylation, histone modifications, and non-coding RNA, which may interact directly with the primary nucleotide sequence and regulate gene expression. Methyltransferases are enzymes that methylate DNA and their support elements, including histones, to change genetic activity and chromatin structure. DNA methylation is a powerful epigenetic mechanism, important in the preservation of DNA structure, chromosomal stability, chromosome inactivation and even activation. It is a natural consequence of aging and cell differentiation, but is also acknowledged as an important modifier of disease risk. In DNA methylation, a methyl group is added to a region where a cytosine base 5' is linked to a guanine by enzymes called DNA methyltransferases. Recent studies have looked into the role of that process in the pathogenesis of AAA. A major study evaluated the DNA of control peripheral blood mononuclear cells from AAA patients and reported that global DNA methylation was significantly higher for men with large major AAA compared to small AAA and controls. Smooth muscle cells from isolated vessels from aneurysm patients show altered DNA methylation levels. Advanced age, smoking, and inflammation are the primary risk factors for AAA, and may have a substantial impact on DNA methylation patterns. Studies of aging find hypomethylation throughout the genome and hypermethylation of the senescence/senility-specific promoter. Smokers have lower methylation levels than non-smokers. Smoking cessation results in partial restoration of DNA methylation patterns, but never to the same levels found in non-smokers. However, it is still unclear whether DNA methylation changes are a cause or a consequence of inflammatory and degenerative processes.^57^

### Pathophysiology

Smooth muscle cell (SMC) loss, extracellular matrix (ECM) destruction, inflammation, and oxidative stress are key phenomena in the pathophysiology of AAA.^58^ Recent *in vivo* and *in vitro* studies in genetics and epigenetics have shown that certain patterns of SMC differentiation and proliferation, combined with structural changes in the ECM, lead to senile degeneration and subsequent dilation of the arterial wall. The studies also detected cellular and fluid infiltrates typical of inflammatory reactions.^59,60^

The five main physiopathological processes observed in AAA formation are:

1. Changes to connective tissue proteins;

2. Imbalance between metalloproteinases and tissue inhibitors of metalloproteinases (both produced by SMC);

3. Chronic inflammation with cytokine release, as well as metalloproteinases by neutrophils and macrophages;

4. SMC transdifferentiation (turning them into macrophage-like cells);

5. Early cell death (apoptosis).

One factor suggesting a key role for SMC in the pathogenesis of AAA is that these aortic abdominal cells come from a different embryonic lineage than other aortic segments, making the infrarenal segment more prone to enlargement. The peculiar embryonic origin of abdominal aortic SMC leads to a specific condition for gene transcription in those cells, with different cell content, genetic activity, and histological structure.

Elastin degradation in the infrarenal aorta is greater than in ascending aortic segments or the aortic arch, and is one of the most powerful mechanisms promotion dilation in this particular aortic segment. All these factors make it more susceptible to specific clinical conditions.^61^

In general, inflammation is a trademark of aneurysm formation, and its role in AAA is well-documented compared to thoracic aortic aneurysms. Initially, neutrophils infiltrate the aortic wall very early, though only transiently. They are sources of metalloproteinases and oxygen free radicals that can trigger ECM degradation and weakening of the aortic wall.^62^ SMC are more consistently influenced by another type of inflammatory cells: macrophages. Macrophages are hematopoietic cells. A relatively recent development is that even differentiated tissue cells, such as vessel SMC, can “trans” differentiate into macrophage-like metalloproteinase producers. This is a well-documented phenomenon in tissue samples from human AAA vessel walls.^63-65^

We now know that the DNA demethylation process may be responsible for changes in protein coding and synthesis that ultimately result in the phenotypic expression of certain genetic codes. Histone H3/Lisin K4 demethylation of the MyH11 gene has been shown to be specific to SMC in guine pig as well as human tissue. This epigenetic change may be a clue to where the inflammatory process begins, to the imbalance between metalloproteinases and their tissue inhibitor factor, leading to the degeneration and degradation of the extracellular matrix of the aortic wall and subsequent AAA formation.

One of the earliest tissue changes identified in the pathophysiology of AAA is the increased concentration of highly reactive oxygen species, such as superoxide. These chemical compounds, known to cause oxidative stress, can induce and potentiate pro-inflammatory gene activity, increase local metalloproteinase concentration, and cause SMC apoptosis. Administering vitamin E as an antioxidant has led to reductions in AAA size and rupture in animal models.^66-69^

### Risk factors

For practical purposes, in developing these guidelines, risk factors were related to the most expressive and best documented odds ratios found in the literature.^70^

#### Age

Age is one of the most important risk factors for AAA development. Compared to a man aged 40-44, the risk increases almost 200-fold for a man aged 75-79 (0.83 *versus* 164 per 100,000). Most studies use 65 as the age cutoff, the inflection point in the AAA prevalence curve.

#### Gender

There is a wide consensus in the literature that AAA is more prevalent in males. The overwhelming majority of studies considering this variable points to a higher likelihood of diagnosis for men and a higher risk of rupture for women (see section on AAA in women). The Male:Female odds ratio ranges from 4.26 to 8.25 (mean 5.93), according a recent meta-analysis which included thirteen studies that looked into gender differences in AAA.

#### Smoking

Increased risk of AAA in current and former smokers ranges from 1.20 to 7.30 (mean 2.97), according to six recent studies. Current smokers have aneurysms at younger ages. In addition, current smokers are at higher risk of AAA than former smokers, and the risk increases proportionally with time of smoking.

#### Systemic hypertension

Diagnosis of hypertension increases the risk of being diagnosed with AAA. The risk increases 1.55 times, ranging from 1.02 to 2.34.

#### Diabetes mellitus

Diabetes mellitus diagnosis is unrelated with significant risk of AAA, having actually been considered a protective factor rather than a risk factor. Though the subject is controversial, diabetes is associated with a 1.18 risk of AAA. Since the confidence interval of this odds ratio ranges from 0.99 to 1.41, crossing the 1.0 neutral threshold, current evidence leads us to state that diabetes is not a significant risk factor for AAA, but neither is it a protective factor.

#### Coronary artery disease

Closely related to peripheral arterial disease (PAD), coronary artery disease (CAD), when present, also increases the likelihood of AAA. According to recent studies, a CAD diagnosis increases the risk of AAA 2.29 times (from 1.75 to 3.01).

#### Family history

Though relatively rare, some studies do include data on the family history of AAA patients. However, this is a more relevant risk factor in practice, since the risk of first-degree relatives being diagnosed with an aneurysm and their complications is almost 10 times higher. Studies point to an odds ratio of 9.64 (ranging from 1.72 to 53.98).

#### Sedentary lifestyle

The impact of lifestyle on noncommunicable chronic disease risk is a growing cause of concern, and represents one of the most important risk factors for PAD and for CAD in general. In the case of AAA, there is little evidence of a direct relation with physical exercise at the moment. Adequate blood pressure (BP) control is known to improve general cardiovascular health. Performing moderate-intensity aerobic exercise at least 3-4 times a week, 30-60 minutes per session, achieves that goal. However, resistance training can increase central aortic BP, so benefits for patients with aneurysms are less well understood. In theory, increases in BP can contribute to subsequent aortic growth and aneurysm complications. High-intensity isotonic and isometric training can increase systolic BP to approximately 300 mmHg with associated Valsalva maneuver. Further longitudinal studies are required.

Recommendantions regarding etiology and physiopathology are on Table 2.

**Table 2:** Recommendations for environmental and genetic risk factors for the genesis of abdominal aortic aneurysms.

**Recommendation**	**Level of evidence**
Considering the role of epigenetic changes on the phenotypic expression of genes related to the genesis of the dilation may be useful to understand the cause of abdominal aortic aneurysms. Cases with genetic-familial history have a higher likelihood of rupture and lower association with heart disease, facts which may be decisive for an indication of earlier intervention.	IIa
Presence of chronic inflammation, oxidative stress, and transdifferentiation of smooth muscle cells of the aortic wall into macrophages producing extracellular matrix-degrading proteolytic enzymes seems reasonable. However, an analysis of inflammatory markers still requires validation for use as prognostic factor.	IIb
Age over 65, male gender, smoking, systemic hypertension, family history, and coronary artery disease are factors that significantly increase the risk of being diagnosed with an abdominal aortic aneurysm.	IIa

## CLINICAL STATUS

Most AAA patients are asymptomatic. At times, some mention feeling a pulse in their stomach. Asymptomatic aneurysms are occasionally found during routine abdominal palpation or by imaging examinations performed for other purposes. Since aneurysm progression means growth, it can compress neighboring structures, causing various symptoms:

- For duodenal compression, symptoms may include vomiting;
- Vena cava compression can cause lower limb edema, progressing to vein thrombosis;
- Ureter compression can result in hydronephrosis and even renal failure;
- Spinal cord compression may cause back pain and sometimes progresses to vertebral body erosion;
- Radicular compression causes neuropathic symptoms in lower limbs.

An additional symptom is lower limb ischemia caused by aortic aneurysm thrombosis or distal artery embolism. In case of acute expansion of the aneurysm, an important symptom is intense abdominal pain in the aneurysm area, usually subsiding only after the aneurysm is repaired. When the aneurysm ruptures, its most frequent complication, the patient usually indicates high-intensity abdominal pain, and may also swoon or pass out due to hypotension.

## DIAGNOSIS

AAA can be diagnosed by clinical examination, but is primarily associated with the use of imaging methods, whether directly or as an incidental finding of examinations performed for other purposes. This section assess different diagnostic methods for AAA, including clinical diagnosis and imaging methods. In imaging methods, we assess their capacity to diagnose and detail aneurysm anatomy, in addition to their advantages and disadvantages.^71,72^

### Clinical examination

Physical examination may reveal the presence of an pulsatile abdominal mass, but its sensitivity is low, especially in obese patients or those with large abdominal circumference^73,74^

### Imaging examinations

Ultrasonography: Doppler and non-Doppler abdominal ultrasonography is the examination of choice for AAA diagnosis. As a low-cost, non-invasive, and widely available test, it is the most frequently used in initial diagnosis.^75^ Some authors have shown the high sensitivity and specificity of ultrasounds to diagnose abdominal aneurysms, which can exceed 95 percent. However, it is an examiner-dependent method, and has limitations: different methods among examiners, obesity, presence of gas in intestinal loops, diameter variations between systole and diastole, limited view of suprarenal aorta, inability to create imaging sequences for later reconstruction and planning.^76^

Ultrasonography has also become an important diagnostic method that does not require the use of iodinated contrast material or radiation. However, it has some limitations, cannot provide enough detail for surgical planning, and does not enable the surgeon to manipulate and reconstruct images for treatment planning purposes, whether open or endovascular repair.

Computed tomography: computed tomography (CT) is a more reproducible method than ultrasonography and does not depend on the examiner for the interpretation and manipulation of images. Interobserver variability is also lower than for ultrasonography.^77^ In addition, tomography allows for a single examination to assess the thoracic and thoracoabdominal aorta, as well as the iliac and femoral arteries, essential for preoperative planning. It can also better detail aneurysms for surgical planning, providing measurements and characteristics of the aneurysm wall, proximal neck, and presence and location of main and accessory renal arteries, as well as accesses for treatment and possible presence of synchronous aneurysms. Radiation is inherent to tomography, but the use of contrast materials can be avoided, or at least reduced, in various situations with less complex anatomy, as well as in the case of allergies and renal dysfunction.

Standardizing measurements is still required, however, such as establishing the diameter from the outer edge of the aortic wall. Greater access to medical imaging software, with the use of centerlines and multiplanar and three-dimensional reconstructions, has enabled greater accuracy and reproducibility in measurements. Software advances have even enabled us to fuse CT images with intraoperative digital angiography images for greater surgical precision. The diameter of the aneurysm and the sealing zones (proximal neck and iliac arteries) should be measured on the true axial direction of the aorta, thus avoiding errors attributed to vessel tortuosity and inclination.

Though there are limits associated with tomography equipment, software availability, and patient characteristics, such as artifacts and renal dysfunction, it should be stressed that the ideal examination uses iodinated contrast material, axial sections with thickness equal to or greater than 0.2 cm, and appropriate software to analyze the images using multiplanar and three-dimensional reconstruction.^77,78^

Other imaging methods:

Simple spin or abdominal x-rays may incidentally discover AAAs, especially in heavily calcified aortas. These methods are not indicated for that purpose, however. Magnetic nuclear resonance (MNR) has limitations compared to tomography, such as availability, claustrophobia, and presence of metallic devices. However, an MNR does not require the use of radiation and iodinated contrast material. It is neither easily interpreted nor widely adopted by vascular surgeons. For aneurysms, there are few studies comparing resonances with the gold standard method, CT angiography.^77,78^

Positron emission tomography (PET/CT) is of limited use for aneurysms, adopted for select cases of inflammatory aneurysms, mycotic aneurysms, and graft infection, where one can identify increased metabolic activity.^79^

Digital subtraction angiography is not an adequate method for diagnosis of aortic aneurysms. Though widely used in the past, it does not enable an accurate measurement of aortic diameter, showing only the true lumen. Aneurysm dimensions may also be understated due to the presence of a parietal thrombus or arterial wall thickening. Angiography is an invasive method, requiring arterial access, and should not be used as a standalone diagnostic method, but rather intraoperatively during endovascular repair.

Recommendations for diagnosis of AAAs can be found in Table 3.

**Table 3:** Recommendations for diagnostic methods for abdominal aortic aneurysm patients.

**Recommendation**	**Level of evidence**
In cases of suspected abdominal aortic aneurysm, standalone clinical examination for diagnosis is not recommended.	IIb
Ultrasonography is the recommended examination for initial diagnosis in investigations of abdominal aortic aneurysms.	I
Ultrasonography is the examination of choice to track small aneurysms, for which there is no surgical planning at the moment.	I
Computed tomography angiography is the examination of choice in therapeutic decision-making and surgical planning for abdominal aortic aneurysms.	I
The aortic diameter should be measured from the outer edge of the vessel wall.	IIb
Imaging processing for CT angiography and multiplanar reconstruction using three perpendicular planes should be used to correctly measure diameters.	IIb

## TREATMENT

### Clinical treatment, indications for surgical treatment, regulatory aspects, choice of devices

#### Clinical treatment

The primary goal of medical treatment for this group of patients is to retard growth, lower the risk of rupture, and consequently obviate the need for surgical or endovascular repair. Periodical imaging examinations (such as Doppler ultrasound and CT angiography) are important for monitoring growth. In addition, strict blood pressure control, treatment for dyslipidemia and diabetes, smoking cessation, and assessment of other risk factors for atherosclerosis are also recommended.

#### Hypertension control

There is a well established association between uncontrolled hypertension and increased frequency of cardiovascular, acute myocardial infarction (AMI), and strokes, as well as rapid aneurysm growth or aortic dissection. The use of beta-blockers and antihypertensives (angiotensin II receptor blockers) to maintain systolic pressure below 130 mmHg and diastolic pressure below 80 mmHg, associated with statins, retards growth as well as minimizes the frequency of these events.

According to the American College of Cardiology/American Heart Association (ACC/AHA)^80^ and European Society of Cardiology (ESC) guidelines,^11^ treatment of AAAs with angiotensin-converting enzyme inhibitor (ACEI) and beta-blockers is a Class IIa recommendation, meaning the benefits of the treatment outweigh the risks and that using the treatment is reasonable. However, the weight of the evidence supporting the use of ACEIs and beta-blockers for AAA has Level of Evidence B, meaning the evidence comes primarily from nonrandomized or observational studies.^81-85^

#### Use of statins

Several studies looked into the impact of statin therapy on aortic aneurysm growth, with mixed results. While some found statin therapy can retard the growth of aortic aneurysms and reduce the risk of rupture, others found no significant impact.

Reducing LDL levels by 50 percent in patients 75 and younger led to a decrease in the number of strokes and cardiovascular events. Another meta-analysis showed that statin use retarded aneurysm growth due to its action on the matrix metalloproteinase-9 or interleukin-6 concentrations. That reduction had a favorable effect on the process of medial degeneration of the aortic wall while acting on the progression of inflammation and atherosclerosis.

A meta-analysis of several studies published in the Journal of Vascular Surgery in 2018 found that statin therapy is associated with a significant reduction in the risk of aneurysm expansion and aneurysm-related deaths, as well as a significant reduction in abdominal aortic aneurysm growth rates in patients who underwent repair.^85^

Statin use before and/or after endovascular treatment of AAA is associated with a 5-year increase in survival compared to the group that did not use statin; however, it should be stressed that these studies have limitations, and further research is required to fully understand the impact of statin therapy on aortic aneurysm growth. It should also be stressed that statin therapy is not specific to aortic aneurysms, and its use should be considered as part of a more comprehesive approach to the management of risk factors associated with aortic aneurysms.^82,86-89^

#### Smoking cessation

Smoking cessation is considered a critical recommendation for treatment of AAA, since it is strongly correlated with aneurysm growth and rupture. Smoking is a key risk factor for the development of AAAs and is associated with higher incidences of aneurysm growth and rupture.

The ACC/AHA^80^ and ESC guidelines^11^ for AAA management list smoking cessation as a Class I recommendation. The level of evidence for smoking cessation in AAA management is listed as Level B, meaning the evidence comes primarily from observational studies or nonrandomized trials. The studies show that smokers are at a much higher risk of developing AAA and of aneurysm rupture than nonsmokers.

Smoking cessation has been shown to reduce the risk of aneurysm growth and rupture by lowering the pressure on the aneurysm, reducing inflammation and oxidative stress in the aorta, and improving aortic wall health. In addition, smoking cessation reduces the risk of other cardiovascular diseases, AMI, stroke, and peripheral arterial disease. Therefore, health professionals should strongly encourage AAA patients to quit smoking and, if necessary, refer them to smoking cessation programs. Smoking is an independent risk factor for AAA development, growth and complications, in addition to increasing the morbidity and mortality of surgical and endovascular repair.^90-92^

#### Use of antiplatelet drugs

Antiplatelet therapy, such as aspirin and clopidogrel, is considered an important aspect of AAA management, used to reduce the risk of cardiovascular events. The ACC/AHA^80^ and ESC guidelines^11^ for AAA management recommend the use of antiplatelet therapy for all AAA patients unless contraindicated. The use of antiplatelet therapy, such as acetylsalicylic acid at 75-162 mg/day, reduces the risk of cardiovascular events in this group of patients and is listed as a Class I recommendation, indicating the benefits of treatment outweigh the risks and that treatment should be administered.

However, when we assess growth speed, the data is controversial. Some studies show that among patients with AAA larger than 4 cm in diameter, aneurysm growth slowed down. On the other hand, a Danish study found higher mortality after rupture in the antiplatelet group compared with nonusers.^93,94^

#### Screening and surveillance

The Aneurysm Detection and Management Study (ADAM) is a multicenter randomized controlled clinical trial with the goal of determining the best strategy to detect and treat AAAs. The study compared two strategies: one, ultrasound screening followed by surveillance for aneurysms with diameter equal to or larger than 3 cm; the other, no screening and treatment of AAAs only when found incidentally or when causing symptoms.^95^

The results showed that ultrasound screening followed by surveillance for aneurysms with diameter equal to or larger than 3 cm was associated with a significant reduction in the number of AAA-related deaths compared to the non-screening strategy. Ultrasound screening followed by surveillance for aneurysms with diameter equal to or larger than 3 cm is listed as Class of Recommendation I and Level of Evidence A. Table 4 summarizes recommendations for clinical treatment and follow-up.

**Table 4:** Recommendations for clinical treatment and follow-up for abdominal aortic aneurysm patients.

**Recommendation**	**Level of evidence**
Consider prescribing beta-blockers and/or angiotensin II receptor blockers to keep blood pressure below 130 and 80 mmHg. Consider prescribing statins to retard the growth or reduce the incidence of cardiovascular events.	IIa
Smoking cessation is recommended for all patients diagnosed with abdominal aortic aneurysm.	IIa
Antiplatelet therapy is recommended for all patients diagnosed with abdominal aortic aneurysm, unless expressly contraindicated.	IIa
Regular abdominal ultrasound for follow-up of patients with aortic diameter greater than 3 cm is recommended.	I

### Indication for surgical and endovascular treatment

Currently, most international guidelines recommend using endovascular aneurysm repair (EVAR) as the treatment of choice for most AAA patients, but open surgery (OS) is still recommended for some patients, especially those poorly suited for EVAR due to the extent, site and anatomy of the aneurysm. Indications for AAA repair, both surgical and endovascular, may be described in simpler terms as:

13. Fusiform aneurysm with diameter larger than 5 cm for women and 5.5 cm for men;

14. Rapid growth fusiform aneurysm, faster than 0.5 cm in 6 months or 1 cm in 1 year;

15. AAA associated with complications and/or symptoms;

16. Dilation shape: saccular aneurysm.

However, key considerations are required in terms of indications for treatment, discussed in the following paragraphs.

## DIAMETER

Even though basing indications for treatment only on large aneurysm diameters is controversial, that criteria is still the most frequently utilized. Therefore, the current thresholds of 5 cm for women and 5.5 cm for men are the most frequently cited for indication for surgical or endovascular treatment. However, the issue is not without controversy in the literature and recommendations may vary, depending on aneurysm site, clinical condition, and the patient's individual risk factors.

The use of EVAR in small and asymptomatic AAAs is still the topic of discussion and research, and recommendation may change as new evidence emerges. Considering the significant decrease in surgical morbidity and mortality from the use of endovascular procedures, early repair of small aneurysms (4-5 cm) is once again the subject of ongoing debate. However, currently available data shows no evidence of the benefits of early treatment for small aneurysms compared to conservative treatment. The UK Small Aneurysm Trial, ADAM, and Comparison of surveillance versus aortic endografting for small aneurysm repair (CAESAR): results from a randomised trial^96-98^ corroborate these premises—even though there is significant crossover in the groups compared in all three studies, as well as the finding that one in every six aneurysms in clinical follow-ups lose optimal anatomy for endovascular repair.^95-99^ Randomized trials have yet to find evidence of the effectiveness of repair for aneurysms smaller than 5.5 cm, but that does not mean that they cannot be more efficient.^100^ Variables such as delays in referrals, the conditions of health system access, and mortality rates at the center where the intervention is to be performed all impact these numbers. Therefore, it is plausible that with aneurysms between 5 and 5.5 cm, a given intervention may be justified.^6,99,100^

For aneurysms smaller than 4 cm in diameter, with no growth or significant symptoms, rigorous monitoring with imaging examinations is usually recommended. For aneurysms between 4 and 5 cm in diameter, with no growth or significant symptoms, rigorous monitoring with imaging examinations is recommended. EVAR may be considered for aneurysms with significant growth or in case of other risk factors for rupture.

## RAPID GROWTH

Two factors associated with aneurysm rupture are its diameter and rapid growth rate. The mean annual growth rate for AAAs is approximately 0.26 cm per year for aneurysms smaller than 5 cm, increasing to 0.5 cm per year for aneurysms larger than 5 cm. Therefore, faster growth, i.e., greater than 0.5 cm in 6 months^80^ or 1 cm in 1 year,^6,80^ is considered a primary criteria for elective AAA repais.

## PRESENCE OF SYMPTOMS

AAAs are typically asymptomatic, and often found incidentally during imaging examinations prescribed for other conditions. It should be noted that AAA symptoms may be subtle, and often barely perceptible. However, as the aneurysm increases, it may cause symptoms such as:

17. Abdominal or back pain;

18. Back pain radiating to the inguinal region;

19. Feeling of fullness or abdominal discomfort;

20. Pulsating feeling in the abdomen.

Multivariate analysis of a number of studies has determined that the strongest predictors of risk of rupture are rapid documented expansion and presence of significant abdominal or back pain, regardless of AAA size.^101-104^

## SACCULAR SHAPE

Aneurysms are considered saccular when the alteration or deformity is found only on one side of the aorta, creating a focal dilation in saccular shape. Saccular aneurysms are less frequent, and data on their natural progression is scarce. According to studies, they are more frequent in women and, compared to fusiform aneurysms, symptoms manifest at smaller diameters. Overall, endovascular or surgical repair is indicated for saccular aneurysms, despite the lack of data and the lack of consensus regarding their size.

In general, the level of evidence for treatment of saccular aneurysms is moderate to low. This is due to the lack of high-quality randomized controlled trials for this type of aneurysm. Most of the evidence available comes from observational studies and case series, which can provide useful information, but are considered less reliable than randomized controlled trials. For example, the Society for Vascular Surgery recommends endovascular repair for saccular aneurysms smaller than 5 cm in diameter, but lists it as a weak recommendation.^7^ The reason is the low quality of the evidence supporting that recommendation, and the decision to treat should be based on the patient's individual characteristics and preferences. Do keep in mind that treatment recommendations for saccular aneurysms may vary, depending on the guidelines or specific sources used, and are based on the current state of knowledge and understanding about the condition.^105,106^

Recommendations for elective surgical repair of AAA can be found in Table 5.

**Table 5:** Recommendations for elective surgical repair for asymptomatic abdominal aortic aneurysm patients.

**Recommendation**	**Level of evidence**
Conventional or endovascular surgical repair is recommended for fusiform aortic aneurysms greater than 5 cm in diameter for women and 5.5 cm for men.	I
Elective abdominal aortic aneurysm repair may justified for diameters between 5 and 5.5 cm based on the patient's clinical and anatomical conditions, health system access, hospital mortality rate.	IIb
Conventional or endovascular surgical repair is recommended for fusiform aortic aneurysms with growth rates greater than 5 cm per year.	Ib
Conventional or endovascular surgical repair is recommended for symptomatic fusiform aortic aneurysms.	IIb
Conventional or endovascular surgical repair is recommended for saccular aortic aneurysms regardless of diameter.	IIb

### Conventional and endovascular surgery

AAAs can be treated with two types of procedure: EVAR and OS. For over 50 years, surgical procedures with the interposition of a straight or bifurcated graft was the first choice of treatment for AAA repair. However, with the development of new endovascular procedures, the strategy came to be replaced by minimally invasive procedures. Despite the high technological level involved, the issue of whether one technique is superior to the other on the short, medium, and long term has always been controversial. Currently, there is consensus in the literature that given the same risk conditions, i.e., patients with the same clinical characteristics and adequate anatomy for endovascular repair, both approaches can be used, with similar outcomes—therefore, the decision about which technique to employ should be made jointly by the medical team and the patient. There is Class of Recommendation I and Quality Level A data in the literature in that direction.^107,108^

Choice of procedure depends on several factors, including patient age, risk factors, and aneurysm size and site. The physician can discuss the best option after assessing the patient's medical history and clinical condition. Several studies and international guidelines are used to guide the treatment of AAA patients.^6,7,12,80^

Physicians should also consider the Brazilian Abdominal Aortic Aneurysm Treatment Guidelines published in August 2016 by the Brazilian Unified Health System (SUS) National Committee for Technology Incorporation (CONITEC).^109^ Based on a thorough literature review and analysis of outcomes from endovascular treatment of AAA compared to conventional surgery in SUS reference hospitals, the guideline strongly recommends the use of endovascular repair to treat AAAs, reserving OS exclusively for situations where EVAR is not possible.

Long term outcomes may be influenced by many different factors related to patient characteristics, quality of repair, and extent of follow-up. In addition, long-term studies also show that the durability of the endografts used in EVAR may be limited, especially first-generation grafts, and reintervention may be necessary in the future in order to replace, complement, or repair endograft migration. Again, it should be stressed that rates of reintervention and adverse events are usually more frequent for EVAR compared to OS, but the mortality rates are still lower.

Currently, most international guidelines and randomized trials recommend EVAR as the treatment of choice for most AAA patients. These are all considered high-quality studies, providing strong evidence for the use of EVAR, but OS is still recommended for some patients, specially those poorly suited for endovascular repair due to the extent, site and anatomy of the aneurysm.

#### Decision-making

The decision-making process for AAA treatment should be individualized, based on patient characteristics. The decision should be discussed by a multidisciplinary team involving a vascular surgeon as well as other specialists, such as interventional practitioners and cardiologists. A multidisciplinary approach enables the team to consider a wide range of factors that might influence treatment choice. The approach is key to determine whether the patient meets the conditions for various treatment options as well as to consider the long term outcomes and possible complications of each option. A multidisciplinary team can integrate various perspectives and sources of knowledge, leading to a more accurate and comprehensive decision-making process and better outcomes for patients. The collective approach also enables the team to manage possible complications and to coordinate patient follow-up, which may improve continuity of care. Shared decision-making in medicine, now a major topic of discussion in the area, where the patient is at the center of the decision-making process, still has room to grow in AAA management.

#### Adherence to instructions

IFUs are mandatory for endovascular repair of aneurysms to ensure the safety and efficacy of the product and the procedure. They provide information such as indications, contraindications, warnings, precautions, and instructions for use of endovascular devices, such as the endografts used in EVAR. Noncompliance with IFUs can lead to complications, such as device failure, endoleaks, and device migration, among others. In some cases, complications may require reintervention or conversion to OS. However, according to the literature, approximately 42 percent of cases met the most conservative recommendations of device IFUs; and 69 percent met the most liberal recommendations, while all others fail to meet recommendations of use.^13^

Adherence to IFUs includes following proper implantation procedures, ensuring correct sizing and placement of device, and monitoring patient condition during and after the procedure to detect potential complications. It is also important to note that IFUs may change over time as new or updated information becomes available, so practitioners should keep abreast of the most recent version of the IFU and contact the manufacturer for additional information.

### Conventional open treatment

#### Indication for open repair

Indications for OS for AAA include asymptomatic and rapidly expanding aneurysms, distal embolization or rupture, and do not differ from indications for EVAR. On the other hand, OS is indicated for most infected aneurysms or when requiring conversion after unsuccessful endovascular repair.

Major contraindications for OS include hostile abdomen, comorbidities (especially heart and renal), and short life expectancy.^7,38,110^

#### Open repair technique

The steps of OS procedures have changed little over the last 7 decades. Typically, a xiphoid-to-pubis midline incision is made and the retroperitoneum approached after right visceral rotation. Dissection of the retroperitoneum is carried out from the proximal neck of the aneurysm to the common iliac arteries or iliac bifurcation, depending on patient anatomy. After systemic heparinization, aortic and iliac clamping is performed, the aneurysm sac is opened, and the lumbar arteries are ligated. A straight or bifurcated graft is then interposed; end-to-end anastomoses are then created using continuous sutures. A teflon ring can be used to reinforce the proximal anastomosis in select cases. After releasing the vascular clamps, the anastomoses and the ligation sites of the lumbar arteries are inspected for bleeding. The aneurysm sac is then brought over the graft, and laparosynthesis is performed.

#### Maintenance of pelvic and visceral flow

Pelvic and visceral perfusion depends on the communication between the superior mesenteric, inferior mesenteric, and hypogastric arteries. Occlusion of hypogastric arteries may lead to erectile dysfunction, buttock claudication and, more rarely, to colon and medular ischemia. During OS, all efforts should be directed at maintaining flow to at least one of the hypogastric arteries.

The risk of colon ischemia increases significantly when revascularization excludes both hypogastric arteries (aortobifemoral bypass with hypogastric exclusion).^6,7^ In a Canadian prospective study, risk of colon ischemia increased eightfold when both iliac arteries were excluded (from 0.3 to 2.6 percent).^111^

Inferior mesenteric artery reimplantation to prevent colon ischemia has conflicting results. Considering reimplantation is reasonable in very specific situations, such as previous colectomy, occluded collateral pathways between the superior mesenteric artery (SMA) and the inferior mesenteric artery (IMA), SMA occlusion and/or stenosis, or absence of the arc of Riolan.^112,113^ A 2006 randomized prospective trial concluded that IMA reimplantation could be beneficial for elderly patients and those with considerable intraoperative blood loss.^114^ The Canadian prospective study^111^ found that postoperative bleeding was more frequent in patients who underwent reimplantation (5 percent of the series).

#### Type of incision

##### Transverse versus midline incision

A randomized prospective study from 2005 assessed select patients randomized to transverse versus longitudinal incision.^115^ Logistic regression analysis after over 4 years found, for a small study group comprised of 69 patients, a higher rate of incisional hernia for the midline incision group (p = 0.010). Transverse incision is a reasonable recommendation for COPD patients because of its lower respiratory restriction.^7^

##### Midline versus retroperitoneal incision

Even randomized trials comparing midline to retroperitoneal incisions had conflicting results.^116-118^ Only two measurable variables—shorter period of adynamic ileus and earlier feeding—favored retroperitoneal incision. Limited exposure of the right renal artery and right iliac artery are drawbacks of retroperitoneal incisions. In the prospective randomized trial conducted by Sieunarine et al.,^116^ no significant differences were found between the two incisions, except for bulging and longer and more intense pain in the retroperitoneal group. On the other hand, longitudinal incisions were associated with more frequent hernias. It seems clear that the retroperitoneal incision should be reserved for cases of hostile abdomen and select cases of inflammatory aneurysm^119^ and horseshoe kidney.

##### Mesh reinforcement in midline incision

A randomized trial^120^ and a 2018 meta-analysis^121^ have shown that the prophylactic use of mesh reinforcement reduces the risk of incisional hernia in xiphoid-to-pubis midline incisions. However, there was no long-term follow-up for these groups and the number of reinterventions was not reported for a more appropriate analysis. Table 6 lists recommendations for open surgery for AAAs.

**Table 6:** Recommendations for open surgical repair for abdominal aneurysms.

**Recommendation**	**Level of evidence**
Preserving flow into at least one internal iliac artery is recommended, both in open surgery and endovascular procedures.	I
Using a transverse incision in chronic obstructive pulmonary disease patients is recommended.	IIb
A retroperitoneal incision is recommended in cases of horseshoe kidney, inflammatory aneurysm, and hostile abdomen.	IIa

#### Juxtarenal aneurysm

By definition, a juxtarenal aneurysm is characterized by a proximal neck short enough to demand suprarenal clamping in open repair, but no anatomical involvement of the aorta at the origin of the renal arteries.^122^ Approximately 15 percent of AAAs are classified as juxtarenal.^122^ Several clinical series do not to properly define this anatomical status, include pararenal aneurysms, and fail to provide long-term outcomes.

There are no randomized controlled trials comparing OS to EVAR at the moment, which represents the main obstacle to comparing the two techniques. It is important to note that branched (BrEVAR) and fenestrated endovascular repair (FEVAR), with low rates of complications, are performed at highly experienced centers with low mortality rates. The decision on whether to use open or endovascular repair for a juxtarenal aneurysm is absolutely multifactorial. Therefore, this document has no intention of comparing the two techniques.

The low mortality rate of OS for juxtarenal aneurysms is evident in various clinical series and meta-analyses. A 2010 meta-analysis assessed the results of OS, including 1,256 patients from 21 nonrandomized trials from the MEDLINE, EMBASE, and Cochrane databases. Perioperative mortality was 2.9 percent (95% CI, 1.8 to 4.6) and incidence of new onset of dialysis was 3.3 percent (95% CI, 2.4 to 4.5).^123^ A recent retrospective study in five french academic centers,^124^ including 315 consecutive patients, also found low mortality for OS (0.9 percent).

With the advent of endovascular repair with branched, parallel stent, or fenestrated endografts,^125,126^ several clinical series and a few meta-analyses have compared OS to endovascular repair. A 2022 meta-analysis confirmed the excellent outcomes of fenestrated endografts in treating juxtarenal aneurysms,^127^ but with no significant reduction in mortality, compared to endovascular treatment; the latter was associated with a higher number of reinterventions, despite lower morbidity. That same year saw the publication of a meta-analysis by Doonan et al.,^128^ which included pararenal aneurysms and several endovascular procedures. That systematic review found lower mortality rates for EVAR. A more recent meta-analysis (UK-COMPASS) of 7,000 patients compared OS to various forms of endovascular repair,^129^ including the use of conventional endografts outside IFUs, parallel stent grafts, and fenestrated endografts. There was a lower mortality rate for EVAR, but difference was not seen at 30 months.

A comparative study in elderly patients found no difference in operative mortality for OS and fenestrated endografts,^130^ but one should bear in mind that comparisons are not possible, and that even with paired patients, anatomical differences, patient selection criteria, and differences in team experience, both for OS and for FEVAR and BrEVAR, present difficulties for this kind of analysis.

The obvious obstacles for a wider indication of fenestrated endografts are related to their high cost, low availability, and requirement of proper training in endovascular techniques. On the other hand, OS of juxtarenal aneurysms has its own technical difficulties and requires additional surgical team expertise and highly specific postoperative intensive care.

OS should preferably be performed by teams treating AAA with in-hospital mortality rates less than 5 percent and depends on the number of repairs completed per year at the center.^131^ This means endovascular repair should be reserved for patients with considerable comorbidities and/or centers completing high volumes of endovascular procedures.

Table 7 summarizes OS and endovascular indications for juxtarenal aneurysms.

**Table 7:** Recommendations for repair of juxtarenal aneurysms.

**Recommendation**	**Level of evidence**
Open surgery is recommended for juxtarenal aneurysms in centers reporting mortality rates below 5 percent.	IIb

### Endovascular treatment of infrarenal aneurysms

EVAR has staked its place as an important technical advance and has become the therapy of choice is several countries. The primary goal of EVAR is to achieve a proximal and distal seal, preventing contact between blood and the aneurysm wall and ultimately preventing rupture. Since the first implantation, and since the first in Brazil in 1994, many endografts have been modified in terms of diameter, material, proximal fixation mechanism, and navigability, among others. Many anatomical constraints have been overcome, and hundreds of vascular surgeons have been trained in the technique; today, it is widely available throughout the country, both in the private health insurance system and in the Brazilian Unified Health System (SUS). IFUs may vary among manufacturers, and we strongly recommend checking the anatomical requirements in the product's manual.

There are many randomized controlled trials on EVAR. One of the most important is the Randomized Controlled Trial of Endovascular Repair versus Open Repair for Abdominal Aortic Aneurysm (EVAR 1).^132^ The trial showed EVAR was associated with a lower short-term risk of death compared to OS. Another important study is Endovascular Aneurysm Repair (EVAR 2), where patients considered unfit for OS underwent EVAR with no improvement in mortality rates and increased need for reintervention.^133^

DREAM is an additional randomized trial to find lower perioperative mortality in the EVAR group.^134^ As well as previous studies, it also showed EVAR was associated with lower risk of death and lower risk of complications compared to OS, despite the higher number of reinterventions.^135^ That advantage evidently favors EVAR during the first 6 months, but does not hold over the long term. Even long-term trials, such as Open versus Endovascular Repair of Abdominal Aortic Aneurysm (OVER), show that survival at long-term follow-up periods (4-8 years) was similar between both groups.^136^

In 2008, a meta-analysis of randomized and observational studies which included 42 studies and totaled 21,178 patients compared OS to EVAR. In elective procedures, the findings were shorter surgery times, less blood loss, lower 30-day mortality, shorter hospital stays, shorter ICU stays, and fewer cardiac and respiratory complications in patients who underwent EVAR. The authors recommend EVAR be the treatment of choice for patients with adequate anatomy, both for elective and emergency surgeries.^137^

The meta-analysis by Sajid et al.^138^ included three randomized trials comparing EVAR to OS (totaling 1,468 patients), confirming EVAR is associated with lower operative mortality, less postoperative pain, shorter ICU stays, and shorter hospital stays. The authors conclude EVAR can be recommended as the treatment of choice for elderly and high-risk patients. A Cochrane Review published in 2014 compared EVAR to OS in AAA repair. Five studies were included, finding a statistical difference favoring EVAR for short-term mortality, but no medium and long-term difference. The reintervention rate in the EVAR group was statistically higher compared to open surgery, but the result should be interpreted with caution, given the heterogeneity between studies. Most reinterventions used endovascular procedures and were associated with low mortality.^139^

The evidence indicates that the decision-making should involve the patient, especially frail and very high surgical risk patients, some of who possibly should be advised not to undergo an operation. For all others, with adequate anatomy and good to moderate surgical risk, EVAR and OS may be suggested as therapies.

Recommendations for EVAR for AAA can be found in Table 8.

**Table 8:** Recommendations for endovascular repair for abdominal aneurysms.

**Recommendation**	**Level of evidence**
Endovascular repair is recommended as the preferred mode of treatment in the presence of a trained team and favorable anatomical conditions.	IIa
Open repair is recommended for young patients and those with low clinical and surgical risk.	IIa
Ultrasound-guided percutaneous access and closing at the conventional surgical access site is recommended to reduce operation time, blood loss, length of hospital stay, wound healing time, and pain.	IIb

## ACCESS CHOICE FOR EVAR

The greater availability of percutaneous closure devices and low-profile endografts have made ultrasound-guided percutaneous access and closure more feasible. Two randomized trials^140,141^ and a major retrospective review found favorable results for percutaneous access and closure of the common femoral artery, with shorter operative time, less blood loss, and better patient-centered results, such as less pain.

The PEVAR trial showed total percutaneous access and closure of the common femoral artery for EVAR patients with adequate anatomy was not inferior.^140^ The Percutaneous access in Endovascular Repair vs Open (PiERO) trial found less pain and improved wound healing among patients who underwent percutaneous access compared to those who underwent groin access approaches.^141^ There was no difference in the incidence of infection at the access site between groups. In addition, a multicenter observational study of common femoral artery access showed a significant reduction in groin hematomas with routine ultrasound-guided accesses.^142^

In patients undergoing endovascular repair of AAAs with adequate common femoral artery anatomy, ultrasound-guided percutaneous access and closing at the conventional surgical access site is recommended to reduce operation time, blood loss, length of hospital stay, wound healing time, and pain.^80^

Table 8 lists a recommendation regarding access for EVAR.

### Endovascular treatment of juxtarenal aneurysms

EVAR has lower mortality rates, short operative time and shorter hospital stays compared to OS in the short and medium term, making it the most widely used treatment method at present.^132,134,143^ However, all commercially available endografts have inclusion criteria defined in their IFUs. These instructions establish the minimum anatomical requirements to use these devices. For most endografts, minimum proximal neck length is 1-1.5 cm, maximum angulation of 45-60º, and absence of calcification and circumferential thrombus.^144^

Despite these explicit recommendations in instructions for use, a retrospective study using a database of patients who underwent endovascular repair showed only 42 percent of patients had anatomy that met the most conservative criteria, and 69 percent met the most liberal definition.^13^ In follow-up, these patients had aneurysm sac enlargement > 0.5 cm in 41 percent of cases. The risk factors associated with enlargement were: presence of endoleak, age above 80, proximal neck diameter > 2.8 cm, angulation > 60º, and common iliac artery diameter > 2 cm.

The presence of a hostile neck, especially short, reverse conical or irregular necks, increases the risk of endoleak fourfold, and the risk of aneurysm-related death ninefold in 1 year.^145^ The combination of hostile neck criteria and implantation of the endograft in off-label anatomies, i.e., those that did not meet the IFUs, increases the need for adjunctive procedures, presence of intraoperative endoleaks, and all-cause mortality.^146^

Therefore, endovascular repair of short neck, juxtarenal or pararenal aneurysms requires more advanced techniques than conventional EVAR to ensure an adequate sealing zone, with a high rate of technical success, as well as a long-lasting repair.

### Parallel stenting

One of those techniques is the use of parallel stents, whether chimney, periscope or sandwich stenting.^147-149^ The advantage of this technique is that it does not use customized devices, which take time to manufacture, but it has the drawback of forming “gutters” that can lead to endoleaks.

Several studies about the procedure are registered rather than prospective studies, suffer from patient selection bias, and problems associated with definition, procedure standardization, patency assessment, and long-term follow-up.^147^ Most data comes from the PERICLES registry, and 95 percent of the 517 patients had juxtarenal aneurysms.^150^ For elective cases, 30-day mortality was 3.7 percent. The incidence of transient renal dysfunction was 28 percent, and 3 percent required permanent dialysis. Technical success was achieved in 97.1 percent of cases, while 2.9 percent of patients had persistent endoleaks. Global survival at 17 months was 79 percent. Chimney patency in patients for whom imaging examinations were available was 94 percent. Mean aneurysm sac regression was 0.44 cm, though there was no mention of how many patients presented no regression.

In a systematic review of the literature, the incidence of endoleaks was 7.6 percent, compared to 3.7 percent for FEVAR.^151^ The better outcomes from parallel stenting come from patients where the physician can create a sealing zone ≥ 1.5 cm, oversize the endograft by 30 percent, and use two chimneys maximum.^151,152^ With the development of fenestrated and branched grafts, the use of parallel stenting is increasingly the province of emergency situations, with anatomy unsuited for FEVAR, or as a salvage procedure in cases of inadvertent occlusion of branch vessels.

### Endoanchors

Endosuture devices were developed to increase endograft fixation to the aortic wall, improving alignment and preventing migration of type Ia endoleaks in cases where conventional EVAR would not be enough due to short or angulated necks. Experimental studies show the use of these devices increases the strength required to dislodge the graft from the aortic wall, approaching or even exceeding that of conventionally hand-sutured grafts.^153^

The use of this device is relatively simple, adding on average only 17 minutes to total operative time,^154^ and it has a short learning curve.

In the ANCHOR multicenter registry, endoanchors were deployed prophylactically in 208 cases where surgeons rated patient anatomy as at high risk of migration or development of Ia endoleaks. In total, 78.3 percent of patients enrolled met the criteria for hostile neck. The technical success rate was 95.2 percent. At 14 months follow-up there were no ruptures, migration, or conversion to open surgery. In patients submitted to control CT, 1.5 percent had type Ia endoleaks. Aneurysm sac > 0.5 cm decreased in 42.9 percent of patients, while it increased in 1.6 percent. Major limitations in this registry were incomplete follow-up and the absence of a control group.^155^

### Physician modified endografts

Several techniques may be used for bench modifications of conventional endografts, such as creating fenestrations, scallops, or branches to incorporate visceral arteries, establishing a fixation zone in patients with inadequate anatomy for conventional grafts. These techniques have the benefit of obviating the wait time for manufacturing a customized graft, and are usually deployed in emergency situations, in high surgical risk patients, or at institutions where customized grafts are not available, whether in Brazil or abroad.

With the ongoing development of customized grafts, the use of modified devices in elective procedures is increasingly restricted. In their retrospective study, Oderich et al.^156^ observed a time shift, where physician-modified endografts (PMEGs) were more widely used in the first years of endovascular treatment for juxtarenal, pararenal or thoracoabdominal aneurysms, while custom-made devices (CMD) were more frequently used in more recent years. In that comparison, patients treated with PMEGs had more comorbidities and larger aneurysms. Technical success was higher with CMD grafts (99.5 versus 98 percent, p = 0.02), and 30-day mortality was higher for the PMEG group (5.5 versus 0 percent, p = 0.0018). At 3-year follow-up, survival and primary and secondary branch vessel patency were similar for both groups.^156^ More recently, a large case series with 5-year follow-up published by a group with extensive experience in PMEGs found good patency and branch stability levels.^157^

### Fenestrated and branched endografts

Technical progress and increased experience in endovascular repair have enabled physicians to extend the proximal fixation zone for endografts, incorporating visceral and renal arteries to repair juxtarenal and pararenal aneurysms^6161^. The greatest advantage of fenestrated/branched endovascular repair (FEVAR/BrEVAR) compared to open surgery is that it does not require aortic clamping, avoiding the subsequent risk of renal dysfunction. They also have lower surgical trauma and faster recovery times, which may benefit high surgical risk patients. FEVAR and BrEVAR are challenging techniques. They should be performed at specialty centers, by expert and experienced surgical teams.^6^

Systematic reviews show the safety and efficacy of FEVAR.^108,158-160^ In a review of 14 case series including 751 patients,^108^ in-hospital or 30-day mortality was 4.1 percent, the prevalence of transient renal failure was 11 percent, and 2 percent of patients required permanent dialysis. The GLOBALSTAR database included 318 patients treated with FEVAR in 14 British centers.^126^ The perioperative mortality rate was 4.1%. Rates of patients free from secondary reintervention were 90, 86, and 70 percent at 1, 2, and 3 years, respectively.

Single and multicenter series on fenestrated and branched endografts have shown promising results.^161,162^ When performed by experienced surgeons, technical success was achieved in a large majority of cases (92 to 99.6 percent), with low perioperative mortality rates. At 1-year follow-up, visceral branch vessel patency was also good (96 to 98 percent), and at 3 years, 91 percent of patients were free of aneurysm-related mortality, with a global survival rate of 57 percent.^163^

Recent observational studies have tried to compare the outcomes of open surgery and FEVAR for complex aortic aneurysms. Varkevisser et al.^164^ compared FEVAR, OS for complex aortic aneurysm and EVAR and found a higher risk of death within 30 days for open repair compared to endovascular repair (OR, 4,9; 95% CI 1.4-1.9), and mortality rates comparable to EVAR.^164^ One should also keep in mind that candidates for complex aortic repair such as FEVAR or BrEVAR have also been selected for good to moderate risk, since very frail or very high risk patients might not survive neither open nor endovascular repair.

However, the late reintervention rate is higher after FEVAR compared to open surgery,^108,165^ as well as persistent kidney injury, and 3-year mortality (excluding perioperative deaths) (HR 1.7; 95% CI 1.1-2.6).^165^ Like for infrarenal endovascular repair, the available data show similar findings, with an initial benefit for survival rates, but diminishing advantages over time and higher reintervention rates. Therefore, FEVAR may be more beneficial for moderate to high surgical risk patients, who are more likely to suffer from perioperative complications.

In addition to branch vessel patency, another critical point for FEVAR/BrEVAR is spinal cord ischemia, especially associated with greater extents of aortic coverage. The manufacturing of customized grafts or modification of off-the-shelf grafts have been described as ways of reducing the length of aortic coverage.^166,167^ Another critical point is absolutely the manufacturing time for customized grafts, which means that PMEGs and off-the-shelf grafts are likely to retain an important role for emergency cases and symptomatic aneurysms in the near future.^157,168^

Therefore, for young, good surgical risk patients, OS is the recommended alternative, while for moderate to high surgical risk patients, endovascular repair is indicated, preferably FEVAR/BrEVAR, as long as they are anatomically possible. Treatment options should be discussed with patients and family members, taking into consideration the advantages and disadvantages of each technique. In patients who underwent endovascular treatment, rigorous follow-up with annual imaging examinations are required.^161^

Table 9 summarizes recommendations for endovascular treatment of juxtarenal and pararenal aneurysms.

**Table 9:** Recommendations for endovascular treatment of juxtarenal and pararenal aortic aneurysms.

**Recommendation**	**Level of evidence**
In young patients at low clinical risk, open surgical repair of juxtarenal or pararenal aneurysms greater than 5.5 cm is recommended.	I
In patients at moderate to high surgical risk and symptomatic patients, consider endovascular repair using one of the available techniques.	IIa
In frail and very high surgical risk patients, consider nonsurgical treatment, with open or endovascular repair, using a shared decision-making process.	IIa

## POSTOPERATIVE FOLLOW-UP

After treatment of AAAs, the goal of follow-up is to avoid aneurysm-related complications or deaths. After open surgery, the formation of pseudoaneurysms at anastomoses or the dilation and formation of new aortic or iliac artery aneurysms is unusual at initial follow-up, with rates of around 1 percent at 5 years, 6-12 percent at 10 years, and up to 35 percent at 15 years.^169-171^

In patients who underwent endovascular treatment, the rate of complications and need for reintervention is significantly higher, and the most frequent complications are endoleaks. Aneurysm sac enlargement without detectable endoleak, endograft migration, and endograft failure may also be found.

Though postoperative follow-up with imaging examinations is recommended from the outset of endovascular repair of AAAs, that plan is rarely followed to the letter, with compliance failures in over 60 percent of cases.^172,173^ The rate of late aneurym rupture after endovascular treatment can be as high as 5 percent in 8 years, and it should be stressed that this incidence is closely related to inadequate case selection, especially cases of unfavorable anatomy and noncompliance with device IFUs. These factors may be more relevant than intrinsic failures of endovascular procedures or materials.^13^

Aneurysm sac retraction during follow-up is an important indicator of successful aneurysm exclusion, and has been shown to be a predictor of low risk of complications at 5-year follow-up.^174^ On the other hand, no study has been able to show increased survival or lower rupture rates in patients who underwent rigid follow-up protocols.^174^

Though there is no consensus among the various health services and publications, recommendations for postoperative follow-up after endovascular treatment are based on CT angiography 30 days after index procedure and, in the absence of leaks and with satisfactory sealing zones, patients should undergo annual CT angiographies. In case of endoleak or aneurysm sac enlargement, examinations should be performed every 6 months.^175,176^

Some authors have shown safety in ultrasound follow-up, either standalone or after an initial CT angiography, showing adequate sealing zones and absence of type I or III endoleak.^175,177^ Contrast ultrasonography is still little used, but has superior sensitivity to CT angiography in identifying endoleaks. It is cheaper, does not use radiation, and has no renal toxicity. Its drawbacks are similar to those of conventional ultrasonography: dependence of examiner experience and limitations related to obesity and abdominal gases.^178^ In patients who underwent open surgery, imaging examinations should be performed every 5 years, preferably including CT angiography.^171^

Recommendations for postoperative follow-up for AAAs treated with OS or EVAR can be found in Table 10.

**Table 10:** Recommendations for postoperative follow-up for abdominal aneurysms treated with conventional or endovascular procedures.

**Recommendation**	**Level of evidence**
CT angiography 30 days after endovascular treatment of abdominal aortic aneurysms is recommended at follow-up.	I
After the initial CT angiography, if there is no endoleak or aneurysm growth, the next examination should be performed at 12 months. This examination may be a CT angiography or Doppler ultrasound, depending on patient and health care service characteristics.	I
If the initial 30-day CT angiography finds a type II endoleak, an imaging examination should be repeated at 6 months. This examination may be a CT angiography or Doppler ultrasound, depending on patient and health care service characteristics.	IIb
If the imaging examination at 12 months finds no endoleak, no aneurysm sac growth, and adequate sealing areas, an imaging examination should be performed annually or at longer intervals no greater than once every 5 years.	IIb
CT scans of the aorta and iliac arteries should be performed every 5 years for patients who underwent open repair of abdominal aortic aneurysms.	IIb

## POSTOPERATIVE COMPLICATIONS

### Graft infection

Aortic graft infection is an infection of a primary prosthesis. The concept includes both grafts used in OS and endografts used in endovascular repair. The complication is rare: the literature reports a 1 percent rate of incidence,^179,180^ mostly within one year of the procedure. Recent data found no statistically significant difference between patients who underwent OS and those who underwent endovascular repair.^181^ The primary sources of infection are: contamination during implantation; aortoenteric fistula (AEF) or erosion to adjacent organ (gastrointestinal tract or airways); or, more rarely, hematogenous dissemination.^182^

Early infections (within 3 months of intervention) are frequently associated with fever, abdominal or back pain, and leukocytosis, while in late infections (after 3 months of intervention), symptoms are insidious, such as fatigue, malaise, and weight loss, with or without fever.^179^ AEF patients often have more severe symptoms, such as bleeding, sepsis, and hemorrhagic shock.^183^

Initial supplementary diagnostic tests include laboratory tests, blood culture, and imaging examinations, preferably CT angiography. In cases of suspected AEF or hemorrhage, an endoscopy and/or colonoscopy is indicated. The diagnostic criteria proposed by Lyons et al.^184^ are especially useful in cases of suspected aortic graft infection. Radiological findings include perigraft fluid ≥ 3 months postimplantation, perigraft gas ≥ 7 months postimplantation or increased gas in serial CT scans, abscess, inflammatory changes, rapid aneurysm growth or pseudoaneurysm.^183,184^

Conservative treatment is exceptional, and includes percutaneous drainage and long-term antibiotic therapy. It may be initiated for patients in pre-op preparation or for very high surgical risk patients as palliative care.^179,185,186^

For acceptable surgical risk patients and aortic graft infection, the recommended treatment is complete excision of the prosthesis/endograft and infected tissues as definitive treatment.^179,187^ The choice of arterial reconstruction should be made on an individual basis, taking into account the type of infected graft, surgeon experience, patient status, and available materials.

The techniques available for arterial reconstruction include extra-anatomic or *in situ* bypass with autologous graft, cryopreserved grafts (not available in Brazil), or rifampin-soaked synthetic grafts. There is no evidence for the superiority of any form of graft, and using any of these options is acceptable for stable patients without extensive infection by multiresistant microorganisms.^179,188^

For patients with extensive perigraft abscesses or infection by multiresistant bacteria, such as methicillin-resistant *S. aureus* and *Pseudomonas,* extra-anatomic bypass or *in situ* femoral vein or allograft reconstruction procedures may offer more time free from reinfection.^189^

*In situ* reconstructions using autologous grafts have lower reinfection rates; however, they are associated with longer operative time, incompatibility with artery size, and venous complications in the legs.^189,190^ Unstable patients requiring rapid proximal vascular control and arterial reconstruction should preferably be treated with allografts (if readily available) or rifampin-soaked synthetic grafts.^183,189-191^

Endovascular treatment has a role in situations requiring rapid hemorrhage control, and can increase AEF patient survival when used as bridge therapy to definitive treatment. In patients clinically unable to undergo infected graft excision, endovascular repair may be considered as the definitive treatment. In these cases, lifelong antibiotic therapy should be considered.^179,183,192^

Aortic graft infection has a high early mortality rate, ranging from 15 to 22 percent.^179^ Despite the progressive increase in the treatment of aortic aneurysms in recent years with the advent of endovascular repair, it remains a rare complication and scientific evidence about its treatment consequently remains limited.

Treatment and follow-up for these patients require a multidisciplinary team, involving infectious disease specialists to establish appropriate antibiotic therapy. Intravenous antibiotic therapy is recommended for a period of 6 weeks, followed by oral antibiotics for 3-6 months, depending on the extent of the infection, associated microorganisms, and type of repair.^189,193,194^ Lifelong antibiotic therapy is recommended in select cases, such as patients with extensive infections, resistant microorganisms, *in situ* reconstructions with grafts or endovascular without full resection of the infected graft.^179,192,195,196^

Follow-up should include imaging examinations and laboratory tests every 3-6 months during the first year, and every 6-12 months afterwards.^179^

### Endoleak

EVAR is associated with complications that may lead to endograft failure and rupture of the aneurysm sac over time, thus requiring follow-up and monitoring.^197-200^ The goal of this section is to provide a critical discussion of major post-EVAR complications associated with endoleaks and their implications in follow-up in order to recognize them and treat them before refilling and pressurization of the aneurysm sac.^200,201^

Post-EVAR complications are found in 16 to 33 percent of patients.^200,201^ A more recent study found a 26 percent complication rate, of which 39.4 percent were observed within the first year.^200^ The most frightening complication is an aneurysm rupture, which may be the result of endoleaks, pressurization of the aneurysm (endotension), migration, deterioration of endografts or degeneration of the proximal neck caused by aneurysm progression.^198,200^

*Endoleak* refers to the presence of flow in the aneurysm sac outside the endograft after EVAR,^202^ and is observed in 1/3 of all cases,^199^ though prevalence depends on type of endograft used as well as imaging examinations performed during follow-up.^197,200,201^ Endoleaks are classified as primary/early (present at the time of the repair) or secondary/late (detected postoperatively using prior normal control images),^203^ as well as to the cause of the periendograft flow. The presence of an endoleak affects aneurysm sac retraction over time due to the pressurization of the aneurysm sac.^199^ Approximately half of all leaks (especially type II leaks) resolve spontaneously, without requiring reintervention.^199^ Antiplatelet therapy may increase the risk of endoleaks after EVAR.^204^ Early detection of the complication, before it becomes clinically relevant, enables its treatment and prevents more severe scenarios. Thus, vascular surgeons should be familiar with existing imaging examinations for post-EVAR follow-up in order to diagnose and better manage potential complications.^205^

Currently, patients with anatomies not favoring endovascular treatment increasingly undergo this form of therapy, requiring more adjunctive procedures and suffering from higher rates of secondary intervention, despite improvements in materials. Therefore, surgeons who offer the choice of EVAR for patients are expected to be familiarized with these complications and know how to properly utilize studies and preparatory work, accurately classify the type of leak, and indicate the proper moment for a secondary intervention.^206^

### Type I endoleak

A type I endoleak is defined as an inadequate circumferential sealing in the areas of graft fixation to the aortic wall, promoting persistent direct flow in the aneurysm sac.^197,199,200^ It can be further subdivided into type Ia, when the leak comes from proximal fixation (proximal neck); Ib, with the leak in the distal fixation region (iliac axis); and Ic, when the leaks is caused by direct communication between the aorta and the iliac artery after an occluder plug is placed in the iliac artery when using a mono-iliac endograft.^207^ Since this form of leak involves direct pressurization of the aneurysm sac, often accompanied by aneurysm enlargement and consequently an increased risk of rupture, a type I endoleak should be promptly treated with the aim of excluding the aneurysm from pressurized circulation. Endovascular repair options for type Ia endoleaks include balloon dilation of endograft fixation points with or without stenting or use of endovascular clamps (endoanchors) for endograft tissue fixation to the aortic wall if there was no migration and in the presence of an adequate sealing zone (proximal neck).^208^ More often, proximal extension of the sealing zone is required, including implantation of a proximal cuff or fenestrated endograft. In type Ib endoleaks, extension to the distal iliac artery is usually enough.^208^ However, if the disease progresses by distal neck degeneration, involving the iliac bifurcation, the physician should consider extension to the external iliac artery or the use of a bifurcated iliac endograft. If an endovascular option is not available in a timely manner and the patient meets the criteria for open surgery, conversion is recommended and has acceptable outcomes.^209^

### Type II endoleak

A type II endoleak is defined as retrograde blood flow from collateral aortic branches filling the aneurysm sac. The most frequently involved arteries in type II endoleaks are the inferior mesenteric and lumbar arteries.^203205206^ In case of aneurysm sac enlargement due to suspected type II endoleak, adequate imaging examinations should be performed to rule out other causes, such as inadequate sealing or type III internal endoleak (connection, integrity of graft or suture holes).^210^ It is classified as type IIa when only one collateral branch is involved and type IIb when two or more branches flow into the aneurysm sac.^211^

They are divided into:

21. Early type II, when diagnosed within 30 days post-EVAR;

22. Late type II, when diagnosed between 30 days and 6 months post-EVAR;

23. Persistent type II, when sustained for over 6 months post-EVAR.

In a meta-analysis of 2,367 patients who underwent EVAR, 18 percent had early type II endoleaks that resolved spontaneously, 5 percent had persistent type II endoleaks, and 11 percent had new type II leaks during follow-up.^206^ Approximately half of all patients with persistent or late endoleaks suffered from sac enlargement, and the reintervention rate was 50 percent within 2 years. Factors associated with persistent or recurring type II endoleaks include internal iniliac artery coil embolization, distal graft extension, age over 80, and anatomic characteristics such as number of patent side branches arising from the aneurysm, thrombus in aneurysm sac, and diameter of lumbar (> 0.2 cm) and inferior mesenteric arteries (> 0.3 cm).^212,213^ Preoperative embolization of the aneurysm sac in select patients has been suggested to reduce the risk of developing type II endoleaks,^214,215^ but the benefit of fewer reinterventions or lower incidence of rupture is still controversial.^216^

The progression of most type II endoleaks seems to be benign, but aneurysm rupture may happen even so.^217^ However, in a systematic review of retrospective studies, fewer than 1 percent of type II endoleaks resulted in ruptures, and reintervention was indicated to repair persistent leaks with aneurysm sac enlargement.^210,218^ Though most ruptures appear to be related to aneurysm enlargement, there are also report of ruptures in the absence of aneurysm expansion.^211^ Some centers treat type II endoleaks in case of aneurysm sac enlargement > 1 cm, while others use > 0.5 cm; the latter number is the threshold for detecting aneurysm sac enlargement when comparing two imaging examinations using the same technique.^206^

Endovascular repair may be performed using embolization of the aneurysm sac and/or feedback vessels using transarterial, translumbar, transcaval or transealing (between the iliac branch of the endograft and the native iliac artery wall) approaches, with a wide variety of devices.^210,218^ Technical success is achieved in 60 to 80 percent of cases; however, there is no objective definition of indication and management for these cases, which may impact interpretation.^206^

Surgical treatment options include laparoscopic ligation or open repair of side branches flowing back into the aneurysm sac, suturing of the ostia under direct visualization after opening the aneurysm sac or surgically removing the endograft, with conversion to conventional surgery in cases of failure of endovascular treatment.^206,210,219^

### Type III endoleak

A type III endoleak can be defined as a secondary leak after a structural failure of the endograft. It is classified as type IIIa if caused by disconnection between components, and type IIIb if caused by manufacturing defects, with the latter further subdivided into those with holes larger or smaller than 0.2 cm. These endoleaks may also be caused by malpositioned endografts with inadequate superposition, proximal or distal endograft migration, or material fatigue.^197,199,200^ It has an incidence rate of 2.1 percent within 4 years post-EVAR, with type IIIa accounting for 56 percent of cases and type IIIb for 44 percent.^220^ However, with the use of more modern endografts, the incidence rate can fall to 1 percent.

Similar to type I endoleaks, in type III there is direct pressurization of the aneurysm sac, with subsequent risk of rupture.^217^ Therefore, immediate endovascular repair is recommended; the most widely used options are implantation of iliac extension, coaxial cuff at the leak site, implantation of new bifurcated endograft, or conversion to mono-iliac endograft, followed by revascularization of the controlateral limb using femoral-femoral crossover bypass. Conversion to open surgery is required only if the endovascular measures described are unable to control the leak.^220,221^

### Type IV endoleak

Type IV endoleaks are very rare in clinical practice, related to graft fabric porosity, and may be related to the use of anticoagulant or antiplatelet therapy. The blood leaks through intact fabric; in the vast majority of cases it is resolved within 30 days of the procedure, and is considered benign.^197,199,200^ According to a review of post-EVAR ruptures reported in the literature until 2008, no cases of ruptures caused by type IV leaks were found.^217^ Type IV leaks are rare for most modern devices and do not require reintervention.

### Type V endoleak or endotension

The type V endoleak, also known as endotension, is the presence of sac enlargement without an identifiable endoleak. The incidence rate ranges from 1.5 to 5 percent, and all other types of endoleak must be ruled out before a definitive diagnosis.^197,199,200^ Several possible mechanisms for endotension have been suggested, including endograft permeability, resulting in direct transmission of pressure through endografts to the aortic wall, or the use first-generation polytetrafluoroethylene endografts, which might hinder thrombus organization and fibrinolysis in the aneurysm sac.^222^ However, given the definition, cases classified as endotension might be caused by a form of endoleak undeterminable by current imaging technologies.^222^ Treatment sis recommended for cases of significant aneurysm sac enlargement (> 1 cm) and consists in open surgery to realign or remove the endograft.^223^

### Migration

Conceptually, endograft migration is defined as endograft movement > 1 cm compared to fixed anatomic reference points, checked against the midline of tomographic reconstructions, or any migration resulting in symptoms or reintervention.^224^ Endograft migration used to be a common event, and most studies on risk factors for proximal device migration were performed using case series involving first-generation endografts, but the development of suprarenal or infrarenal active fixation in more modern endografts led to decreased prevalence of migration.^225,226^

Migration can result in type I endoleak, disconnection between endograft components, kinking, and branch vessel occlusion. Factors contributing to proximal migration include short proximal fixation, angulated neck, large aneurysms, endograft type,^224,227,228^ and oversized endografts (> 30 percent); the latter is controversial, but there is evidence that it may also contribute to migration.^229,230^ Disease progression with proximal neck dilation may cause migration, and is related to initial neck diameter.^231^

Migration may also occur due to changes in aneurysm morphology or aneurysm sac retraction after EVAR. Iliac fixation length (distal neck) of at least > 2 cm, or preferably up to the iliac bifurcation, reduces the risk of endograft migration.^232,233^

Table 11 summarizes recommendations for treatment of endoleaks.

**Table 11:** Recommendations for treatment of endoleaks after endovascular repair of abdominal aortic aneurysms.

**Recommendation**	**Level of evidence**
In patients with type I endoleak after endovascular repair of an abdominal aortic aneurysm, early reintervention is recommended to achieve aneurysm sealing and exclusion, preferably via endovascular procedure.	I
Preserving at least one hypogastric artery in case of iliac bifurcation involvement.	I
Reintervention for type II endoleak treatment after endovascular repair for abdominal aortic aneurysm should be considered in the presence of significant aneurysm growth, preferably via endovascular procedure.	IIa
Aneurysm sac diameter growth greater than 1 cm found at follow-up after endovascular repair of abdominal aortic aneurysm, using the same imaging and measurement techniques, may be considered a reasonable criteria to detect significant growth in cases of type II endoleak.	IIb
In patients with type III endoleak after endovascular repair of an abdominal aortic aneurysm, reintervention is recommended, preferably via endovascular procedure.	IIa
Significant aneurysm sac growth after endovascular repair of abdominal aortic aneurysm without endoleak identified in standard imaging examinations requires considering additional diagnostic assessment and possible endograft realignment or explantation.	IIa

## RUPTURED ABDOMINAL AORTIC ANEURYSM

A ruptured aortic aneurysm is one of the most dramatic conditions in emergency care patients. It is lethal in the vast majority of cases, if not all, when untreated. The primary factors for rate of rupture are size, morphology, and growth rate.^234^ In Brazil, it is estimated that between 2000 and 2016, ruptures were the direct cause of 38,000 deaths, representing 55 percent of all aortic aneurysm-related mortality.^4^ Historically, the in-hospital mortality rate for ruptured aneurysms was 50 percent, but widespread endovascular treatment has led to a drop in mortality, which currently ranges from 20 to 30 percent.^234-237^

The classic symptom triad of ruptured AAAs includes hypotension, abdominal pain, and pulsatile mass. There may be other manifestations, however, such as back pain and groin pain; in case of visceral perforation, there may be gastrointestinal tract bleeding, with subsequent hematemesis or melaena. There is a peculiar form of rupture, i.e., rupture into the inferior vena cava, that manifests as an aortocaval fistula, with abdominal fremitus as its frequent sign, which may be associated with acute and severe heart failure and paradoxical pulmonary embolism.^6^

Anatomically, the rupture site is also associated with prognostic factors. AAA rupture is the most frequent, and there are differences if it occurs in the anterior or posterior aneurysm wall. In the interior wall, it usually also perforates the retroperitoneum and flows into the peritoneal cavity, which hinders tamponading, and many patients in this situation die before they can have access to hospital care. Ruptured aneurysm tamponade is often caused by rupture of the posterior wall, and the spinal column and iliopsoas muscles help contain the bleeding, giving the patient a higher statistical likelihood of arriving at the hospital alive to receive care.

Diagnosis begins with clinical suspicion, and is possibly easier when the patient is aware of a prior diagnosis of AAA. The challenge may be maintain a high degree of suspicion for hypotensive patients with a life-threatening condition, but no apparent cause.

The establishment of protocols and the volume and experience of the health care service are associated with better outcomes and higher chances of survival throughout the world.^7,238-240^ Both diagnosis and referral for treatment require a pre-established workflow in hospital departments.

Patients with ruptured aneurysms should receive initial care at the emergency room, where large-bore peripheral access should be inserted and the first sample for laboratory tests collected. Volume replacement should be used with the goal of achieving permissive hypotension,^7,238^ which means keeping the patient conscious and their systolic blood pressure between 70 and 90 mmHg. The stabilization is recommended in case the patient has to be transferred to a center with an aortic team and prepared for both types of therapy, endovascular or conventional repair.

Abdominal ultrasound and chest X-rays are usually widely available in emergency rooms; the first can reveal free fluid in the abdominal cavity or costal recess, while the latter can show opacification of a hemithorax, or increased cardiac area in case of thoracic

However, these tests cannot rule out rupture, because the bleeding may be contained, and the diagnostic examination of choice is a CT angiography of the aorta, which should include planes from the ascending aorta and the arch to the common femoral arteries. Adequate technique is crucial for surgical planning.

CT angiography can find clear or indirect signs of abdominal aortic rupture. These are blurring of the psoas, aortic wall discontinuity, contrast extravasation, crescent sign or thrombus fissuration, and intraperitoneal or retroperitoneal hematoma.^241^

Historically, the treatment of ruptured aneurysms had a high mortality rate, but the advent of endovascular surgery has significantly decreased that rate. After 20 years of consistent endovascular repairs, there is strong evidence, from randomized trials and meta-analyses, that faced with a ruptured aortic aneurysm, endovascular repair is absolutely better than open surgery.^6,7,234,236,237,242,243^

The better outcome translates into lower medium and long-term mortality as well as lower 30-day mortality rates, in addition to lower complications rates in recovery, such as ostomies and amputations, and better quality of life.^242^ Therefore, training the team, providing material and operating rooms for open or endovascular repair can directly impact the likelihood that patients survive ruptures.^238^

The main limitation to EVAR in cases of ruptured AAA is patient anatomy, more specifically the diameter and proximal neck length of the endograft fixation site. Therefore, in the absence of these conditions or of materials and teams for endovascular treatment, open surgery is required, and the reference center should be ready for both options.

Permissive hypotension and patient preparation for anesthesia with the surgical team in position for intervention may reduce the chance of overcoming the tamponade. Therefore, coordination with the anesthesia team is critical. Volume and blood product replacement are part of an effective therapy.

Both repairs may require supraceliac aortic clamping in case of severe hypovolemic shock. This may be an open procedure, via midline laparotomy, blunt dissection of the lesser curvature of the stomach, and aortic clamping to the spinal cord. It may also be an endovascular procedure, and even performed under local anesthesia, via femoral access by dissection, placement of 12FR introducer sheath and ipsilateral guidewire for the aortic occluding balloon, which can be inflated at the level of the 12th thoracic vertebra.^238^ It should be stressed that hemodynamically unstable patients have high mortality rates, and systolic pressure below 70 mmHg is an independent predictor of death. Finally, keep in mind that in cases of this nature, submitting an unstable patient to conventional repair is almost always fatal, and endovascular repair is even more justified in that condition.^7^

EVAR has an additional advantage over conventional treatment of ruptured aneurysms, i.e., the possibility of administering local anesthesia and using sedation only if required^238^—current evidence indicates better outcomes for patients treated under local anesthesia, hemodynamics permitting.^244-246^ In addition, not intubating patients with ruptured aneurysms is associated with a higher likelihood of survival.^234^

The choice between bifurcated and mono-iliac endografts has to involve surgeon experience and preferences, since achieving rapid hemostasis is key. The time to catheterization of the contralateral branch vessel should not be extended, but avoiding a femoral-femoral crossover bypass graft also has advantages for the patient. Overall, prioritizing the use of bifurcated endografts seems reasonable as group experience advances.^6^

Recommendations for treatment of ruptured AAAs can be found in Table 12.

**Table 12:** Recommendations for treatment of ruptured abdominal aortic aneurysms.

**Recommendation**	**Level of evidence**
In cases of ruptured abdominal aortic aneurysm, endovascular repair is recommended.	I
If the hospital is unable to offer endovascular repair, transferring the patient to a specialty center is acceptable, as long as their clinical condition and hemodynamics allow it.	IIa
Volume replacement should be used in order to keep the patient conscious and their systolic blood pressure between 70 and 90 mmHg.	I
In patients arriving at the hospital, consider stabilization and thoracoabdominal CT angiography to assist surgical planning.	IIb
The use of bifurcated endografts is preferred, given adequate anatomical conditions.	IIb

## INFLAMMATORY ANEURYSM

Inflammatory aortic aneurysms are characterized by a thick layer of inflammatory tissue frequently associated with periaortic and retroperitoneal fibrosis and adherence to neighboring organs and tissues. Exuberant inflammatory infiltrates in the adventitia are among the primary differences between it and an atherosclerotic degenerative aneurysm.^247^ The condition makes open surgical treatment difficult, and potentially lethal.^248^ The literature reports incidence rates ranging from 5 to 18 percent.^248,249^

From a physiopathological perspective, the mechanisms causing the inflammation are still poorly understood. Clinical symptoms, such as back pain, fever, weight loss, and loos of appetite, may be present. Major risk factors identified thus far are male gender, smoking, and genetics^253.255^. Unlike other inflammatory or rheumatological diseases, aortic inflammation is most often isolated, with no involvement of other arteries.^248^

Laboratory tests may be altered, including leukocytes and inflammatory markers, such as C-reactive protein. CT angiography of the aorta reveals signs such as periaortic thickening, which may also include retroperitoneal involvement, and has high diagnostic sensitivity.^247^ Though they are not as sharp and clear as the images produced by CT angiography, abdominal ultrasounds can often identify a hyperechogenic halo around the aortic wall. Positron emission tomography can also reveal periaortic inflammation.^248^

OS can be challenging, and possibly lethal, due to adhesions to adjacent organs, such as the inferior vena cava, ureters, and small intestine, which may require enterectomies.^247,250^ Give this history, endovascular repair is increasingly used to treat inflammatory aneurysms. However, it should be stressed that after analyzing the published data, we still lack randomized and long-term follow-up trials to verify its efficacy and safety.^248,250^

## ANEURYSMS IN WOMEN

AAAs are less common in women compared to men, at a ratio ranging from 1:4 to 1:9 in the literature. There are no randomized clinical trials analyzing AAA in women specifically. However, there are many scientific studies about this important subject. The prevalence of AAAs among people over of 60 is 0.7 percent, and it increases rapidly with age.^100^ Operative mortality increases with age, and women have clinically relevant AAA at older ages compared to men. AAA morphology differs significantly between the two: men tend to have larger aortas, iliac arteries, and femoral arteries than women. Women are older and have smaller AAAs at treatment.^19,36,100,251^

The EVAR 1, DREAM, OVER, and ACE randomized trials found a significant decrease in mortality among open surgery and endovascular repair patients and similar long-term survival.^206^ It is not clear that both genders benefited equally. Women had fewer advantages, but since they were only a small part of the trials (0.6 to 9 percent), a more accurate statistical analysis is not possible.

Several theories have been suggested to explain the differences between male and female AAA patients, but there are still no definitive conclusions. A few studies using statistical data stand out: Ulug et al.,^100^ in a systematic meta-analysis of nine studies conducted between 2005 and 2016, which included 52,018 men and 11,076 women, found a post-EVAR 30-day mortality rate of 1.4 percent for men and 2.3 percent for women. In open surgery, the numbers were worse: 2.8 percent for men, 5.4 percent for women. Sidloff et al.,^252^ in their analysis of the UK National Vascular Registry (United Kingdom, 2010-2014), which included 23,245 patients, of which 13 percent were women, found a 30-day post-EVAR mortality rate of 0.7 percent for men and 1.8 percent for women. In open surgery, mortality numbers were worse: 4 percent for men, 6.9 percent for women. Deery et al., in their analysis of the American College of Surgeons National Surgical Improvement Program (2011-2014), found a 30-day mortality rate of 1.2 percent for men and 3.2 percent for women post-EVAR. In open surgery, the numbers were worse: 4 percent for men, 8 percent for women.^253^ Erben et al.^254^ report a higher rate of complications and reinterventions in women, especially due to arterial thrombosis of the lower limbs.

Though the mortality of AAA treatment decreased exponentially over the last decade, female mortality remains excessively high, especially for open surgery. Wanhainen et al.,^6^ in the Guidelines they organized, found a 6.9 percent mortality rate for women who underwent open repair for AAA, compared to 1.8 percent after endoluminal repair. Though not statistically important, statistically significant higher rates of complications and mortality for women still persist. Recent studies, such as Tumer et al.,^255^ Corsi et al.,^256^ Ilyas et al.,^257^ Tedjawirja et al.^258^ continue to find higher mortality for women.

The causes are multifactorial: even though they have fewer AAAs, women have them at more advanced ages, with more comorbidities, and are generally underdiagnosed. In particular, AAA at advanced ages are more often associated with occlusive arterial disease, which represents a more challenging anatomy for endovascular treatment. For these reasons, women have higher rates of complications and postoperative mortality, both for open surgery and for endovascular treatment. In short, they have worse prognoses. For better prognoses, their AAAs should be treated more often once they reach 4 cm in diameter^36,100,251-259^

In women, AAAs rupture at smaller diameters than men. Solberg et al.^260^ showed that AAAs grow faster in women compared to men. In terms of risk of rupture, a 4.5 cm AAA in women has the same risk as a 5.5 cm one in men. With a diameter of 5 cm, women are at risk of rupture in 1/30 of cases (3.3 percent). It is also known that with identical diameters, the risk of rupture is 4 times greater for women than for men.^19,36,251,259^ The causes may also be multifactorial, but in absolute values, the most important one is that women's arteries are approximately 1/3 smaller than men's in diameter, meaning AAA diameter is proportionally much larger in women, which makes ruptures easier. We also cannot compare anatomical data from English-speaking countries, where most population studies about AAA are conducted, with data for Brazilians, who tend to be smaller, especially women. The data for Asian populations, more similar to Brazilians in size, has been very well analyzed.^261^ Therefore, in recent years, many publications have indicated treating AAA starting at 4.5 cm in diamater in women, and especially not letting it grow past 5 cm, in elective cases.^252,254,257-263^ The surgical risk for endovascular treatment in several studies is under 2 percent. Therefore, it would be logical to recommend this effective form of treatment prophylactically for women with AAA when the diameter is greater than 4.5 cm.

Women are known to often have aortoiliac morphology unfavorable to EVAR—short and angulated necks and smaller iliac and femoral arteries, frequently incompatible with endograft introducer system diameters. Sweet et al.^264^ report that 70 percent of men, but only 40 percent of women, had anatomies compatible with instructions for use for the endografts available in 2011. In previous decades, the situation was even worse. At the moment, there are no endografts designed specifically for women. However, the challenges specific to female anatomy have recently become the topic of specific studies, and adequate devices are being developed to expand indications for EVAR in women. This would also decrease complication rates. Recent data suggests poor historical outcomes may be related to technical problems rather to gender differences themselves. There are three ultra-low profile endograft systems: Incraft^®^, Ovation^®^, and Altura^®^. Others are currently in development. There are long term studies about the first two systems, and they compare favorably to historical results for AAA treatment in women.^265-267^

Women with AAA are treated at older ages, with more comorbidities, and have proportionally larger aneurysms. They frequently have anatomies hostile to EVAR, with small-caliber aortas, iliac arteries, and femoral arteries, as well as more advanced atherothrombotic disease. These situations require specific devices to overcome these challenges. In women, low profile and high flexibility endografts have had better outcomes in terms of technical success and mortality, reducing the complications associated with the procedure.

Improvements in materials, reductions in profile without loss of resistance, and accuracy of implantation should help narrow the gap between outcomes for men and women. Larger patient numbers and more multicenter trials studying the new devices to verify their long-term efficacy and durability are required before a definitive position about the subject can be established.

Recommendations for treatment of AAAs in women can be found in Table 13.

**Table 13:** Recommendations for treatment of abdominal aortic aneurysms in women.

**Recommendation**	**Level of evidence**
Women with abdominal aortic aneurysms with aortic diameter of 4 cm should undergo aneurysm control semiannually.	IIa
Elective treatment of abdominal aortic aneurysms in women with aortic diameter of 5 cm or larger is recommended.	IIa
For endovascular treatment of abdominal aortic aneurysms in women, a device with delivery system dimensions compatible with patient anatomy should be found. Low-profile endografts should be considered, if required.	IIb
