# Supplementary material for: Brazilian Society for Angiology and Vascular Surgery guidelines on abdominal aortic aneurysm
Source: J Vasc Bras. 2023 Oct 30;22:e20230040. doi: 10.1590/1677-5449.202300402 (PMC10648059; doi:10.1590/1677-5449.202300402)
Supplement: Translation

# Translation: Projeto Diretrizes, Sociedade Brasileira de Angiologia e Cirurgia Vascular: aneurisma da aorta abdominal

**DOI:** 10.1590/1677-5449.202300401

**Authors:** Grace Carvajal Mulatti, Edwaldo Edner Joviliano, Adamastor Humberto Pereira, Alexandre Fioranelli, Alexandre Araújo Pereira, André Brito-Queiroz, Arno Von Ristow, Lucas Marcelo Dias Freire, Marcelo Martins da Volta Ferreira, Marco Lourenço, Nelson De Luccia, Pierre Galvagni Silveira, Ricardo de Alvarenga Yoshida, Ronald José Ribeiro Fidelis, Sharbel Mahfuz Boustany, Walter Junior Boim de Araujo, Julio Cesar Peclat de Oliveira

**Affiliations:** 1 Universidade de São Paulo – USP, Hospital das Clínicas – HC, Faculdade de Medicina – FMUSP, São Paulo, SP, Brasil.; 2 Universidade de São Paulo – USP, Faculdade de Medicina de Ribeirão Preto – FMRP, Ribeirão Preto, SP, Brasil.; 3 Universidade Federal do Rio Grande do Sul – UFRGS, Hospital de Clínicas de Porto Alegre – HCPA, Porto Alegre, RS, Brasil.; 4 Santa Casa de São Paulo, Faculdade de Medicina, São Paulo, SP, Brasil.; 5 Universidade Federal da Bahia – UFBA, Hospital Ana Nery, Salvador, BA, Brasil.; 6 Pontifícia Universidade Católica do Rio de Janeiro – PUC-Rio, Rio de Janeiro, RJ, Brasil.; 7 Universidade Estadual de Campinas – UNICAMP, Hospital das Clínicas – HC, Campinas, SP, Brasil.; 8 Serviço Integrado de Técnicas Endovasculares – SITE, Rio de Janeiro, Rio de Janeiro, RJ, Brasil.; 9 Santa Casa de Curitiba, Curitiba, PR, Brasil.; 10 Universidade de São Paulo – USP, Faculdade de Medicina – FM, São Paulo, SP, Brasil.; 11 Universidade Federal de Santa Catarina – UFSC, Florianópolis, SC, Brasil.; 12 Universidade Estadual Paulista “Júlio de Mesquita Filho” – UNESP, Faculdade de Medicina de Botucatu, Botucatu, SP, Brasil.; 13 Universidade Federal da Bahia – UFBA, Salvador, BA, Brasil.; 14 Universidade Federal do Paraná – UFPR, Hospital de Clínicas, Curitiba, PR, Brasil.; 15 Universidade Federal do Estado do Rio de Janeiro – UNIRIO, Rio de Janeiro, RJ, Brasil.

**Keywords:** aneurisma da aorta, aorta, doenças da aorta, procedimentos endovasculares

## Abstract

A Sociedade Brasileira de Angiologia e Cirurgia Vascular, por meio do projeto Diretrizes, apresenta as novas Diretrizes de Aorta Abdominal, referentes aos cuidados de pacientes com aneurisma de aorta abdominal. Para sua elaboração, foram priorizadas diretrizes descritivas, utilizando as bases EMBASE, LILACS e PubMed. As referências incluem ensaios clínicos randomizados, revisões sistemáticas, metanálises e estudos de coorte. A qualidade das evidências foi examinada por uma dupla de coordenadores, com auxílio da ferramenta RoB 2 da Colaboração Cochrane e dos formulários da Newcastle Ottawa Scale. Aneurismas justarrenais, infectados e novas técnicas terapêuticas, principalmente no âmbito endovascular, estão entre os temas estudados. A versão atual das Diretrizes apresenta importantes recomendações para os principais itens que envolvem o diagnóstico, tratamento e acompanhamento de pacientes com aneurisma de aorta abdominal, oferecendo um guia objetivo para prática médica, construído a partir de evidências científicas e amplamente acessível em todo o território nacional.

## INTRODUÇÃO 

O projeto Diretrizes foi criado em 2002, fruto da união do Conselho Federal de Medicina (CFM) e da Associação Médica Brasileira (AMB)^1^. As Diretrizes são um conjunto de informações médicas acerca de um tema, organizadas e embasadas por evidência científica de qualidade. O intuito, naquela época, e que norteia o projeto até hoje, é fornecer informações que possam ajudar o profissional médico na tomada de decisões, diagnósticas, terapêuticas e de acompanhamento a seus pacientes.

O Ministério da Saúde publicou documento em 2016 em que trata da importância das Diretrizes médicas, e sugere uma metodologia para que sejam construídos documentos com boa qualidade científica^2^. A experiência acumulada e a criação de protocolos de padronização de condutas parecem ter influência direta na melhora dos resultados, trazendo redução da morbidade e da mortalidade para o paciente.

A AMB mantém em seu site (https://amb.org.br/projeto-diretrizes/) uma seção na qual médicos em todo o Brasil podem consultar as Diretrizes separadas por temas ou por sociedades médicas de especialidades. Um dos princípios norteadores é o amplo acesso e divulgação dos documentos, e este site permite livre acesso. A Sociedade Brasileira de Angiologia e Cirurgia Vascular (SBACV) tem ampla representatividade, expressa no número de seus associados, que até o ano de 2022 somavam 4.232 sócios. Entendendo que as Diretrizes necessitam de atualização constante para manter um caráter informativo e de segurança para o especialista, a SBACV este ano atualiza e incorpora novas diretrizes à sua biblioteca. O intuito é fornecer um instrumento de trabalho que auxilie o raciocínio clínico, mas preserve a autonomia de atuação do médico, previsto no Código de Ética Médica do CFM.

O aneurisma da aorta abdominal (AAA) é o aneurisma aórtico mais frequente. A doença tem um aumento com a idade, sendo mais prevalente nos pacientes acima de 60 anos. O tabagismo é um dos principais fatores de risco para o seu crescimento e rotura, que é sua complicação mais temida^3^. A rotura, quando ocorre, é letal na maioria dos casos. No Brasil, estima-se que de 2000 a 2016 38.000 pessoas morreram em decorrência direta da rotura, o que representa 55% de todas as mortes relacionadas a aneurisma de aorta^4^.

### Objetivo

Elaboração das novas Diretrizes de Aneurisma de Aorta Abdominal, com metodologia bem definida e ampla divulgação para a sociedade.

## METODOLOGIA

Para elaboração das Diretrizes de Aneurisma da Aorta Abdominal, o Departamento Científico da SBACV selecionou um grupo de autores que atuam na assistência e têm pesquisas científicas consistentes no tema de interesse. As últimas Diretrizes, publicadas em 2015, foram reescritas para responder a novas perguntas e tópicos não cobertos previamente^5^. Os aneurismas justarrenais, os infectados e novas técnicas terapêuticas, principalmente no âmbito endovascular, estão entre os temas estudados.

Previamente ao início dos trabalhos para elaboração do texto, o grupo se reuniu e teve aulas sobre como organizar e avaliar a qualidade das evidências com o Prof. Dr. Wanderley Marques Bernardo, um dos autores do Projeto Diretrizes, com extensa experiência e dedicação a trabalhar com grupos como este. Foram priorizadas diretrizes descritivas que apresentam uma síntese de recomendação. Para elaborar o texto, foram consultadas as seguintes bases na literatura: EMBASE, LILACS e PubMed.

Na base de dados do EMBASE (Elsevier), são listados periódicos indexados, resumos de anais de congresso e notas técnicas de materiais, entre outros. Seu acesso no Brasil pode ser feito gratuitamente pelo portal de periódicos da CAPES. A estratégia PICO – P: população a ser estudada, I: intervenção, C: comparadores, O: desfecho (*outcome*) – foi construída através desse site. A lista de perguntas ou assuntos foi feita previamente pela coordenadora do grupo e os autores consultados para a divisão da redação. Cada autor elaborou uma ou no máximo duas sessões.

Os tópicos abordados neste documento são:

1. Metodologia
2. Definição
3. Epidemiologia e rastreio
4. Etiologia, fisiopatologia e fatores de risco
5. Quadro clínico
6. Diagnóstico
7. Tratamento
  - Indicações clínicas, aspectos regulatórios, escolha de dispositivos
  - Tratamento convencional aberto
  - Tratamento endovascular do aneurisma infrarrenal
  - Tratamento endovascular do aneurisma justarrenal
8. Seguimento clínico
9. Complicações pós-operatórias
  - Infecção de prótese
  - *Endoleak*
10. Aneurisma de aorta abdominal roto
11. Aneurisma inflamatório
12. Aneurisma em mulheres

Três principais artigos foram usados como pontos de partida: a última versão da Diretriz Brasileira de Aneurisma de Aorta (2015)^5^, os Guidelines da European Society of Vascular Surgery (2019)^6^ e os Guidelines da Society for Vascular Surgery (2018)^7^. Também foram levantados artigos de acordo com as seguintes categorias de publicação: ensaios clínicos randomizados, revisões sistemáticas, metanálises e estudos de coorte. Não foram aceitos como fonte de evidências as séries de casos ou relatos de casos, tampouco estudos experimentais.

A qualidade da evidência foi examinada por uma dupla de coordenadores previamente selecionada. Os ensaios clínicos randomizados foram submetidos a avaliação por meio da ferramenta RoB 2 da Colaboração Cochrane^8^ *.* Estudos de coorte, revisões sistemáticas e metanálises foram usados para complementar as respostas, e o instrumento para avaliação da qualidade da evidência foi o New Castle Ottawa Scale^9^.

A qualidade das diretrizes é variável e pode ser auditável, utilizando-se instrumentos específicos. Apesar de existirem vários protocolos de avaliação, o Appraisal of Guidelines for Research and Evaluation (AGREE II) é aquele que mais utilizado para avaliar diretrizes^10^ **.** A avaliação inclui seis domínios: abrangência e propósito, envolvimento de indústrias ou investidores, rigor na coleta de dados, clareza da apresentação, aplicabilidade e independência editorial. O nível aceitável para uma diretriz é atingir uma pontuação acima de 80%. Apenas quatro diretrizes que tratam dos aneurismas da aorta abdominal alcançam essa pontuação (European Society of Cardiology, Society for Vascular Surgery, European Society of Vascular Surgery e National Institute of Health)^6,7,11,12^.

Parece evidente que cada diretriz que cobre determinado assunto ou patologia deve levar em consideração a situação econômica de cada região ou país. Análises de custo-benefício e resultados locais de cada intervenção devem ser realizadas, assim como avaliações das características do sistema de saúde. As recomendações de uma determinada diretriz podem não ser aplicáveis em alguns centros, mas servem para nortear o tratamento mesmo em situações que não sejam as ideais.

Aprovação ética para a elaboração desta Diretriz foi obtida através da Plataforma Brasil e do Comitê de Ética e Pesquisa sob o número de CAAE: 62177722.2.0000.0068. Foi realizado e aprovado o cadastro da SBACV como instituição proponente de pesquisas científicas pela primeira vez desde a sua fundação.

## DEFINIÇÃO

O aneurisma de aorta compreende uma dilatação de dois desvios padrão ou de 50% a mais que o diâmetro esperado para aquela artéria naquela região. Esta diretriz trata especificamente dos aneurismas infrarrenais e, portanto, das dilatações acima de 3 cm de diâmetro. Muito se discute sobre técnicas de mensuração de diâmetro, mas para fins de considerar a maior dilatação, as medidas anteroposterioras e/ou transversais são aceitas, na parede externa da aorta, via ultrassonografia de abdome ou tomografia computadorizada^6,7^.

Existe também muita discussão sobre as definições de colo hostil, colo curto e justarrenais, muito possivelmente devido ao avanço das técnicas de correção endovascular, assim como das indicações de uso habituais de endoprótese (IFUs)^13^. Para fins de padronização adotou-se a classificação de justarrenal ou pararrenal para aquele aneurisma que se estende até uma das artérias renais, sem, contudo, comprometê-la, ou aquele que tem colo abaixo de 1 cm, também denominado colo curto ou hostil^6,7^.

O colo hostil por sua vez é aquele que possui pelo menos uma destas principais características: colo infrarrenal maior que 28 m, ângulo infrarrenal menor que 60^o^, comprimento de colo < 1,5 cm, mais que 50% da circunferência de trombo, colo cônico (aumento de mais de 0,2 cm em um comprimento de 1 cm infrarrenal), boceladura (aumento de mais de 0,3 cm nos primeiros 1,5 cm infrarrenais)^13,14^.

Visto que as fronteiras anatômicas de tratamento foram cruzadas na última década, esta diretriz considerou incluir os reparos abertos e endovasculares de aneurismas justarrenais, infrarrenais e de colo hostil como objeto de estudo, assim como fizeram as últimas diretrizes internacionais^6,7^.

## EPIDEMIOLOGIA E RASTREIO

A prevalência atual de AAA em homens com mais de 65 anos é de 1,7% no programa de rastreamento sueco (adicionalmente, 0,5% já apresentava o diagnóstico)^15^, 1,3% no programa de rastreamento do Reino Unido^16^, 3% na Dinamarca (homens com 65-74 anos)^17^ e 5% nos EUA, onde somente é oferecido rastreamento para pacientes fumantes^18^. No Brasil, não existem dados de rastreamento em nível populacional. Homens apresentam uma prevalência quatro a seis vezes maior em relação às mulheres, em estudos internacionais e nacionais^4,19,20^, e uma metanálise publicada em 2016 estimou uma prevalência de 0,7% em mulheres com mais de 60 anos^19^.

Em países desenvolvidos, a prevalência e incidência de AAA vêm diminuindo significativamente nas últimas décadas, o que é parcialmente atribuído à redução dos índices de tabagismo^6,15,21,22^. Segundo análise dos dados do Datasus publicada em 2020, a mortalidade por AAA no Brasil aumentou no período de 2000-2008, seguido de uma diminuição entre 2008-2016^4^. O Brasil está entre os países que mais apresentou redução nos índices de tabagismo entre 1990-2015, o que pode em parte explicar essa redução^4^.

## RASTREAMENTO

Existem quatro grandes estudos de rastreamento de AAAs em nível populacional, no Reino Unido, Austrália, Dinamarca, e um estudo menor de rastreamento em mulheres no Reino Unido^23-27^, todos realizados em pacientes com mais de 65 anos. Uma revisão da Cochrane^28^ desses estudos, avaliando mortalidade relacionada ao AAA, calculou uma razão de riscos de 0,60 (IC95% 0,47-0,78) em favor do rastreamento. No acompanhamento mais tardio disponível, a mortalidade por qualquer causa foi significativamente menor no grupo rastreado, com razão de riscos de 0,987 (IC95% 0,975-0,999, p = 0,03)^29^. O principal dano associado ao rastreamento está no número de procedimentos eletivos, que aumenta cerca de duas vezes. No entanto, isso é parcialmente compensado pela redução dos procedimentos de emergência^16^. Devido à alta mortalidade associada à ruptura e à baixa morbimortalidade do tratamento eletivo, o número necessário de homens rastreados para evitar uma morte relacionada ao aneurisma seria de 667, e o número de aneurismas tratados, de 1,5^30^. Com os dados que dispomos no momento, a idade ideal para o rastreamento em termos de custo-benefício não pode ser definida. No entanto, atualmente recomenda-se uma ecografia abdominal isolada em homens com mais de 65 anos^6^. Existe baixa evidência para rastreamento em mulheres, visto que o único estudo randomizado sobre a questão não apresentou poder estatístico para uma análise adequada. Portanto, no momento, não se recomenda rastreamento em nível populacional em mulheres^31^.

Estudos relativamente pequenos evidenciaram a associação entre doença arterial periférica e AAA. Contudo, a expectativa de vida diminuída e o alto risco cirúrgico desse tipo de paciente contrabalançam a maior prevalência nesse subgrupo. Não existem até o momento evidências inequívocas para o rastreamento em pacientes com doença arterial periférica^32^. Evidências sólidas correlacionam história familiar positiva com o risco de apresentar AAA, crescimento aórtico rápido e maior risco de rotura. Apesar desse subgrupo não ter sido avaliado adequadamente nos estudos atuais, sugere-se o rastreamento de rotina nesses pacientes em ambos os gêneros^33,34^. Da mesma forma, dada a frequente concomitância de aneurismas periféricos (ilíaca, femoral, poplítea) e AAAs, também se recomenda rastreamento nesse grupo a cada 5-10 anos^35^.

A frequência ideal de exames subsequentes não foi avaliada de forma adequada nos ensaios randomizados, mas uma modelagem utilizando um banco de dados de 15.000 pacientes^36^ sugere que os intervalos devem ser estratificados conforme o diâmetro do aneurisma. Para aneurismas entre 3-3,9 cm sugere-se intervalos de 3 anos entre exames, enquanto para AAA entre 4-4,9 cm recomendam-se exames anuais. Somente quando o aneurisma alcança 5 cm, os intervalos diminuem para 3-6 meses. Apesar de existirem informações limitadas em relação à qual a conduta ideal para pacientes com ectasia de aorta (diâmetro < 3 cm), é justificável nova ecografia em 5-10 anos em pacientes com boa expectativa de vida^37^. As recomendações de rastreio de aneurismas aórticos abdominais encontram-se na Tabela 1.

**Tabela 1:** Graus de recomendação para rastreamento de aneurismas abdominais aórticos

Recomendação	Nível de evidência
Homens devem ser rastreados para aneurisma de aorta abdominal acima dos 65 anos, com uma ultrassonografia de abdome.	IIa
Homens e mulheres com história familiar positiva para aneurisma de aorta abdominal devem ser rastreados a partir dos 50 anos.	IIb
Recomenda-se ultrassonografia para acompanhamento dos diâmetros aórticos acima de 3 cm, sendo que o intervalo vai depender do diâmetro inicial.	I

## ETIOLOGIA, FISIOPATOLOGIA E FATORES DE RISCO 

Poucos são os países que dispõem de programas de rastreamento e diagnóstico precoce do AAA na população em geral. Por isso, há uma limitação real para o conhecimento da verdadeira incidência de novos casos e prevalência de casos já existentes. Consequentemente, temos dados limitados para a elucidação mais precisa dos seus principais fatores de risco. Essa escassez de dados mundiais gera algumas distorções e vieses de interpretação das causas e mecanismos de formação do AAA. Buscar obter esses dados tem efeitos na prática, pois em lugares onde há um programa nacional de diagnóstico e prevenção (como no Reino Unido, Suécia e Austrália) há, nos últimos anos, um flagrante declínio nas taxas de mortalidade e demais complicações do AAA comparadas às mesmas taxas de países de igual nível socioeconômico, como Hungria, Áustria e Romênia^32,38-42^.

Gênero masculino, idade avançada, níveis baixos de lipoproteína de baixa densidade e tabagismo são fatores de risco historicamente relacionados ao AAA, sendo considerados critérios para indicação de exames de triagem^43,44^. Do mesmo modo, estudos demonstram relação de algumas doenças com a coexistência de AAA^11,43,45,46^. São elas: hipertensão, doença arterial periférica, insuficiência cardíaca isquêmica, infarto do miocárdio prévio e doença pulmonar obstrutiva crônica (DPOC)^47,48^.

“Como” e, principalmente, “por que” os aneurismas aparecem são perguntas frequentes vindas de pacientes e familiares que recebem o diagnóstico de AAA, direcionadas ao médico angiologista / cirurgião vascular. Nessas dúvidas, está o desejo natural de entender o que pode ser feito para evitar novas dilatações e suas temidas complicações. Por isso, nesta seção das Diretrizes serão abordados aspectos importantes do estado atual do conhecimento das causas (etiologia), formação (fisiopatologia) e fatores de risco para o AAA.

### A) Etiologia

#### “Hereditário ou Comportamental”?

Ter um AAA é algo que já está pré-determinado no nascimento, ou questões externas ao corpo e relacionadas ao ambiente são mais importantes? Será que vale a máxima de que “o ser humano é tão velho quanto as suas artérias” e as dilatações e obstruções são consequência inevitáveis da senescência hereditariamente programada, ou a senilidade aórtica com dilatação é uma doença previsível e passível de prevenção?

Falar de etiologia dos AAAs (assim como de qualquer outro processo patológico) é necessariamente falar sobre genética e sobre sua irmã mais nova: a epigenética. Ambas tentam explicar o “porquê” a parede de um vaso tão robusto como a aorta enfraquece a ponto de se tornar aneurismática. É fato que os casos de AAA se agrupam em certas famílias e há evidências mostrando forte componente genético no risco^49,50^. Estudos com gêmeos relataram que a herança genética pode chegar a 70%. O registro sueco de gêmeos relatou que um gêmeo monozigótico tem 24% de probabilidade de ter um aneurisma, em oposição a apenas 4,8% em gêmeos dizigóticos se o outro gêmeo tiver um aneurisma^51–54^ . A história familiar positiva mostrou aproximadamente o dobro do risco de desenvolver AAA nesses estudos.

Encontramos também na literatura relatos de que indivíduos com AAA de ocorrência familiar são mais propensos a apresentar ruptura e menos propensos a ter doença cardíaca, quando comparados a casos de aneurismas sem história familiar^55,56^. Isso leva alguns especialistas a considerarem uma intervenção em diâmetros menores que o habitual para os casos não familiares.

A hereditariedade no AAA pode ser mendeliana (um único gene) ou não mendeliana, com causa mais complexa devido a uma variação de genes. Doenças genéticas raras, como a síndrome de Ehlers-Danlos, síndrome de Marfan, síndrome de Loeys-Dietz e displasia fibromuscular, que podem causar AAAs, são exemplos de hereditariedade mendeliana. Entretanto, essas são doenças raras, e análises epidemiológicas revelaram que apenas 10-20% dos pacientes com AAA têm pelo menos um parente com doença genética. Isso pode sugerir que um mecanismo mais complexo, em vez da variação de um único gene, pode estar por trás da genética como causa do AAA. Há mais evidências sugerindo que mecanismos epigenéticos (fatores de risco ambientais e comportamentais) desempenham papel importante na doença vascular e na plasticidade das células lisas musculares do sistema vascular relacionada a esse processo^57^.

A epigenética refere-se a modificações hereditárias e adquiridas no genoma que alteram a expressão gênica sem alterar a sequência do DNA. Em alguns casos, as modificações epigenéticas são estáveis e transmitidas às gerações futuras, mas muitas modificações são relativamente dinâmicas e respondem a sugestões ambientais^58^. Modificações epigenéticas incluem metilação do DNA, modificações de histonas e RNA não codificante, que podem interagir diretamente com a sequência primária de nucleotídeos e regular a expressão gênica. As metiltransferases são enzimas que metilam o DNA e os elementos de suporte, incluindo histonas, para alterar a atividade gênica e a estrutura da cromatina. A metilação do DNA é um poderoso mecanismo epigenético que é importante na manutenção da estrutura do DNA, estabilidade cromossômica, inativação e mesmo ativação de cromossomos. Ocorre naturalmente como resultado do envelhecimento e da diferenciação celular, mas também é reconhecida como um importante modificador do risco de doenças. Na metilação do DNA, um grupo metil é adicionado a uma região onde uma base citosina 5' para uma guanina por enzimas DNA metiltransferase. Estudos recentes têm investigado o papel desse processo na patogênese do AAA. Um grande estudo que avaliou o DNA de células mononucleares do sangue periférico de controles de pacientes com AAA relatou que a metilação global do DNA foi significativamente maior em homens com AAA grandes em comparação com AAA pequenos e controles. Células musculares lisas de vasos isoladas de pacientes com aneurisma mostram níveis alterados de metilação do DNA. O avanço da idade, tabagismo e inflamação são os principais fatores de risco de AAA, que podem ter um impacto substancial nos padrões de metilação do DNA. Estudos do envelhecimento mostraram hipometilação em todo o genoma e hipermetilação específica do promotor da senescência/senilidade. Indivíduos tabagistas têm níveis mais baixos de metilação do que os não fumantes. A cessação do tabagismo resulta na restauração parcial do estado de metilação do DNA, mas esse nunca foi completamente revertido para níveis de não fumantes. Ainda não está claro, entretanto, se as alterações na metilação do DNA são causa ou consequência dos processos inflamatórios-degenerativos^59^.

### B) Fisiopatologia

Perda de células musculares lisas (CML), destruição da matriz extracelular (MEC), inflamação e estresse oxidativo são fenômenos centrais na fisiopatologia do AAA^60^. Estudos recentes *in vivo* e *in vitro* nos campos da genética e epigenética têm demonstrado que determinados padrões de diferenciação e proliferação das CML, associados a mudanças estruturais na MEC, levam a uma degeneração senil da parede arterial e à consequente dilatação. Infiltrados celulares e humorais típicos de reação inflamatória também foram detectados nesses estudos^61,62^.

Os cinco principais processos fisiopatológicos observado na formação do AAA são:

1. Alterações nas proteínas do tecido conectivo;
2. Desequilíbrio entre as metaloproteinases e fatores teciduais inibidores das metaloproteinases (ambos produzidos pelas CML);
3. Inflamação crônica com liberação de citocinas e, também, de metaloproteinases por neutrófilos e macrófagos;
4. Transdiferenciação das CML (transformando-se em células semelhantes a macrófagos);
5. Morte celular precoce (apoptose).

Um dado que sugere um papel central das CML na patogênese do AAA é que essas células na aorta abdominal são de uma linhagem embrionária distinta dos outros segmentos aórticos, o que deixa o segmento infrarrenal mais predisposto ao aumento de diâmetro. A origem embriológica peculiar das CML na aorta abdominal leva a uma condição específica de transcrição genética nessas células, com conteúdo celular, atividade genética e estrutura histológica diferentes.

A degradação da elastina na aorta infrarrenal é maior que a de segmentos da aorta ascendente ou do arco aórtico, sendo um dos mecanismos mais potentes que promovem a dilatação desse segmento específico da aorta. Tudo isso a torna mais suscetível a condições clínicas específicas^63^.

Em geral, a inflamação é uma marca registrada da formação de aneurismas, e seu papel no AAA é bem documentado em comparação com o aneurisma da aorta torácica. No início, neutrófilos se infiltram na parede da aorta muito cedo, embora apenas transitoriamente. Eles são fontes de metaloproteinases e radicais livres de oxigênio que têm o poder de iniciar a degradação da MEC e o enfraquecimento da parede aórtica^64^. As CML sofrem uma influência mais consistente de outra célula inflamatória: o macrófago. Macrófagos são células de origem hematopoiética. O dado relativamente novo é que mesmo células teciduais já diferenciadas, como as CML dos vasos, podem se “trans” diferenciar em outras *macrófago-like* produtores de metaloproteinases. Esse fenômeno já se encontra bem documentado em amostras teciduais de paredes de AAA humano^65-67^.

Sabe-se, hoje, que o processo de desmetilação do DNA pode ser responsável por mudanças na codificação e síntese de proteínas que, em última instância, resultam na expressão fenotípica de determinados códigos genéticos. A desmetilação da Histona H3/Lisina K4 do gene MyH11 provou-se específica para CML em tecidos de cobaias e também de humanos. Essa alteração epigenética pode ser uma pista de onde começa o processo inflamatório, do desequilíbrio entre as metaloproteinases e seus fatores inibidores teciduais, gerando degeneração e degradação da matriz extracelular da parede aórtica e consequente formação dos AAA.

Uma das mais precoces alterações teciduais flagradas na fisiopatologia do AAA é o aumento da concentração de espécime hiper-reativas de oxigênio, como o superóxido. Esses compostos químicos, conhecidos como causadores de estresse oxidativo, são capazes de induzir e potencializar a atividade de genes pró-inflamatórios, aumentar a concentração local de metaloproteinases e causar apoptose de CML. Administração de vitamina E como antioxidante demonstrou levar a reduções no tamanho de AAA e ruptura em modelos animais^68-71^.

### C) Fatores de risco

Para efeito prático da atual diretriz, foram relacionados os fatores de risco com as “razões de risco” mais expressivas e melhor documentadas na literatura até o presente momento^72^.

#### Idade

A idade é um dos fatores de risco mais importantes para o desenvolvimento de um AAA. Comparado a um homem de 40-44 anos, o risco aumenta quase 200 vezes para um homem de 75-79 anos (0,83 *versus* 164 por 100.000). Sessenta e cinco anos é o ponto de corte considerado pela maioria dos estudos, a partir de onde há uma inflexão na curva de prevalência do AAA.

#### Gênero

Há uma concordância ampla na literatura sobre a maior prevalência de AAA em indivíduos do sexo masculino. A maioria absoluta dos estudos que consideraram essa variável aponta para a maior chance de diagnóstico no sexo masculino, e para um maior risco de ruptura no sexo feminino (vide seção específica sobre AAA em mulheres). A razão de risco Homem:Mulher varia de 4,26-8,25 (média de 5,93), conforme metanálise recente realizada incluindo treze estudos que avaliaram diferencialmente a doença por gênero.

#### Tabagismo

O incremento de risco da ocorrência de AAA em pessoas que fumam (ou que já tiveram o hábito de fumar) varia de 1,20-7,30, sendo de 2,97 vezes na média, conforme seis estudos recentes. Tabagistas atuais têm aneurismas em idades mais precoces. Além disso, quem continua fumando tem mais chance de ter um AAA do que quem já parou, e o risco aumenta proporcionalmente ao tempo de tabagismo.

#### Hipertensão arterial sistêmica

A presença do diagnóstico de hipertensão aumenta o risco de o indivíduo ser diagnosticado com AAA. Esse aumento é da ordem de 1,55 vezes, variando de 1,02-2,34.

#### Diabetes mellitus

O diagnóstico de diabetes *mellitus* não está relacionado com aumento de risco significativo de AAA, já tendo sido, inclusive, considerado como “fator de proteção” em vez de “fator de risco”. Apesar de controverso, ter diabetes está associado a um risco de AAA de 1,18. Como o intervalo de confiança desse *odds ratio* vai de 0,99-1,41, cruzando a linha neutra do 1,0, podemos dizer, a partir das atuais evidências, que ser diabético (ou diabética) não é um fator de risco significativo, mas também não é um fator de proteção.

#### Doença coronariana

Intimamente relacionada com a doença arterial periférica (DAP), a doença coronariana (DAC), se presente, também aumenta a possibilidade de a pessoa ter um AAA. Conforme estudos recentes, o diagnóstico de DAC eleva o risco de AAA em 2,29 (de 1,75-3,01).

#### História familiar

São relativamente mais raros os estudos que incluem dados sobre a história familiar de pessoas com AAA. Apesar disso, esse se mostra como o fator de risco mais relevante na prática, pois o risco de parentes de primeiro grau também serem diagnosticados com aneurisma e suas complicações é de quase 10 vezes. Os estudos apontam para um *odds ratio* de 9,64 (variando de 1,72-53,98).

#### Sedentarismo

O impacto do estilo de vida no risco de doenças crônicas não transmissíveis é motivo de preocupação e atenção crescentes, sendo esse um fator de risco dos mais importantes para a DAP e para a DAC de um modo geral. No caso do AAA, poucas são as evidências de relação direta da prática de exercícios disponíveis até o momento. Sabe-se que o controle adequado da pressão arterial (PA) melhora a saúde cardiovascular geral. Realizar atividade aeróbica de intensidade moderada pelo menos 3-4 dias por semana, 30-60 min por sessão, cumpre bem esse objetivo. Entretanto, o treinamento de musculação pode aumentar a PA central da aorta e, portanto, os benefícios para aquelas pessoas com aneurisma da aorta são menos bem compreendidos. Em teoria, aumentos na PA podem contribuir para o crescimento subsequente da aorta e complicações do aneurisma. Exercícios isotônicos e isométricos de grande intensidade podem elevar o nível da PA sistólica para cerca de 300 mmHg, se executados com manobra de Valsalva associada. Mais estudos longitudinais são necessários nesse quesito. Recomendações a respeito da etiologia e fisiopatologia estão listadas na Tabela 2.

**Tabela 2:** Recomendações relacionadas a fatores de risco ambientais e genéticos na gênese do aneurisma abdominal aórtico

Recomendação	Nível de evidência
Pode ser útil para o conhecimento de causa de aneurismas da aorta abdominal considerar o papel das alterações epigenéticas sobre a expressão fenotípica de determinados genes relacionados à gênese da dilatação. Casos de ocorrência genético-familiar têm maior propensão à ruptura e menor associação com doenças cardíacas, fatos que podem ser decisivos na indicação da intervenção mais precoce.	IIa
Pode ser razoável a participação de inflamação crônica, estresse oxidativo e transdiferenciação de células musculares lisas da parede da aorta em macrófagos produtores de enzimas proteolíticas destruidoras da matriz extracelular. Contudo, a análise de marcadores inflamatórios ainda necessita de validação quanto ao seu uso como fator prognóstico.	IIb
Idade maior que 65 anos, gênero masculino, tabagismo, hipertensão arterial sistêmica, história familiar, doença arterial coronariana são os fatores que aumentam de modo significativo o risco do diagnóstico de aneurisma da aorta abdominal.	IIa

## QUADRO CLÍNICO

A maioria dos pacientes com AAA não tem sintomas. Algumas vezes referem ter um coração pulsando na barriga. Esses aneurismas assintomáticos são encontrados ocasionalmente durante o exame médico de rotina de palpação do abdome ou por algum exame de imagem para outra finalidade. Como a evolução do aneurisma é de crescimento, pode ocorrer a compressão de estruturas vizinhas, causando alguns sintomas:

- No caso do duodeno, podem aparecer sintomas como vômitos;
- Compressão da veia cava, pode ocorrer edema de membros inferiores e evoluir para trombose venosa;
- Compressão de ureter, levando à hidronefrose e até mesmo insuficiência renal;
- Compressão de coluna pode causar dores na região posterior e algumas vezes evoluir para erosão de vértebras;
- Compressão de raiz nervosa, causando sintomas neuropáticos nos membros inferiores.

Outro sintoma que pode ocorrer é a isquemia dos membros inferiores por uma trombose do aneurisma de aorta ou por embolia arterial distal. No caso de uma expansão aguda do aneurisma, podemos observar como sintoma dor abdominal, na região do aneurisma, de forte intensidade e que normalmente só regride após a correção do mesmo. Quando acontece a ruptura do aneurisma, que é a complicação mais comum, o paciente normalmente refere dor abdominal de forte intensidade, e pode também ocorrer desmaio pela hipotensão ou desfalecimento.

## DIAGNÓSTICO

O diagnóstico dos AAAs pode ser realizado através do exame clínico, mas está principalmente associado à utilização de métodos de imagem, seja de forma direta ou como achado incidental em exame realizado com outro propósito. Esta seção avalia os diferentes métodos diagnósticos para os AAAs, incluindo o diagnóstico clínico e os métodos de imagem. Nos métodos de imagem, serão avaliados sua capacidade de diagnóstico e detalhamento da anatomia dos aneurismas, além das suas vantagens e desvantagens^73,74^.

### A) Exame clínico

O exame físico pode revelar a presença de massa abdominal pulsátil; entretanto, sua sensibilidade é baixa, principalmente em pacientes obesos ou com circunferência abdominal elevada^75,76^.

### B) Exames de imagem

Ultrassonografia: ultrassonografia abdominal com ou sem Doppler é o exame indicado no diagnóstico dos AAAs. Por ser um exame de baixo custo, não invasivo e amplamente disponível, costuma ser o exame mais utilizado no diagnóstico inicial^77^. Alguns autores demonstraram elevada sensibilidade e especificidade do ultrassom no diagnóstico dos aneurismas abdominais – maiores que 95%. Entretanto, é um exame examinador dependente, que apresenta algumas limitações: metodologias diferentes entre os examinadores, obesidade, presença de gás nas alças intestinais, variações do diâmetro entre a sístole e a diástole, limitação na visualização da aorta suprarrenal, incapacidade de criar séries de imagens sequenciais para posterior reconstrução e planejamento^78^.

A ultrassonografia tem se mostrado um importante método diagnóstico que não usa contraste iodado ou radiação. No entanto, apresenta algumas limitações e não é capaz de oferecer detalhes suficientes para um planejamento cirúrgico, além de não possibilitar a manipulação e reconstrução das imagens pelo cirurgião para planejamento do tratamento, seja este aberto ou endovascular.

Tomografia computadorizada: a tomografia computadorizada é um método mais reprodutível que a ultrassonografia e não dependente do examinador para a interpretação e manipulação das imagens obtidas. A variabilidade entre observadores também é menor que na ultrassonografia^79^. Além disso, a tomografia permite em um único exame avaliar a aorta torácica e toracoabdominal, além das artérias ilíacas e femorais, o que é essencial no planejamento pré-operatório. Ainda, tem maior capacidade de detalhar o aneurisma para o planejamento cirúrgico, mostrando medidas e características da parede do aneurisma, colo proximal, presença e localização das artérias renais principais e acessórias, além dos acessos para o tratamento e possível presença de aneurismas sincrônicos. A radiação é uma característica inerente ao método da tomografia, porém a utilização de contraste pode ser evitada ou pelo menos reduzida em diversas situações com anatomia menos complexa e no caso de alergias ou disfunção renal.

Há necessidade, porém, de padronização das medidas, como o estabelecimento do diâmetro baseado nas medidas desde a parede externa da aorta. O maior acesso à utilização de *softwares* para avaliação das imagens, com a utilização de linhas centrais, reconstruções multiplanares e tridimensionais, tem possibilitado maior precisão e reprodutibilidade das medidas. Os avanços dos *softwares* têm possibilitado inclusive a fusão das imagens de tomografia com as da angiografia digital intraoperatórias, possibilitando maior precisão cirúrgica. O diâmetro do aneurisma e das zonas de selamento – colo proximal e artérias ilíacas deve ser medido no sentido axial verdadeiro da aorta, evitando-se erros atribuídos à inclinação e tortuosidades dos vasos.

Embora possam existir limitações relacionadas ao equipamento de tomografia, disponibilidade de *softwares* e características do paciente como artefatos e disfunção renal, é importante destacar que o exame ideal deve ser realizado com uso de contraste iodado, cortes axiais com espessura igual ou menor que 0,2 cm e que as imagens sejam analisadas em *software* apropriado com reconstruções multiplanares e tridimensionais^79,80^.

### Outros métodos de imagem

As radiografias simples do abdome ou coluna podem incidentalmente identificar AAAs, principalmente em aortas muito calcificadas. Esses exames não são, entretanto, indicados com essa finalidade. A ressonância nuclear magnética (RNM) apresenta algumas limitações em relação à tomografia, como a disponibilidade, claustrofobia e presença de dispositivos metálicos. A RNM prescinde, entretanto, do uso de radiação e contraste iodado. Não é um exame de fácil interpretação e não é largamente adotado pelos médicos cirurgiões vasculares. No campo dos aneurismas, não existem muitos estudos comparativos entre a ressonância e o padrão-ouro – angiotomografia^79,80^.

A tomografia por emissão de pósitrons (PET-TC) é um exame de uso restrito no território dos aneurismas, utilizado em casos selecionados de aneurismas inflamatórios, micóticos ou infecções de próteses, em que se pode identificar atividade metabólica aumentada^81^.

A angiografia com subtração digital não é um método adequado ao diagnóstico dos aneurismas da aorta. Embora muito utilizada no passado, não permite a correta aferição do diâmetro da aorta, mostrando somente seu lúmen verdadeiro. As dimensões do aneurisma podem ser subestimadas devido à presença de trombo parietal ou espessamento da parede. A angiografia é um método invasivo, com necessidade de acesso arterial, e não deve ser utilizada como método diagnóstico isolado, sendo usada no intraoperatório do tratamento endovascular.

Na Tabela 3, encontram-se as recomendações referentes ao diagnóstico dos AAAs.

**Tabela 3:** Recomendações referentes a métodos diagnósticos para os pacientes portadores de aneurisma da aorta abdominal

Recomendação	Nível de evidência
Na suspeita de aneurisma da aorta abdominal não está recomendado exame clínico de forma isolada para o diagnóstico.	IIb
A ultrassonografia é o exame recomendado para o diagnóstico inicial na investigação dos aneurismas da aorta abdominal.	I
A ultrassonografia é o exame de escolha no acompanhamento de aneurismas pequenos, em que ainda não há planejamento cirúrgico.	I
A angiotomografia computadorizada é o exame de escolha na decisão terapêutica e no planejamento cirúrgico dos aneurismas da aorta abdominal.	I
A medida do diâmetro da aorta deve ser realizada utilizando-se como parâmetro a borda externa da parede do vaso.	IIb
O processamento das imagens de angiotomografia computadorizada e a reconstrução multiplanar com três planos perpendiculares devem ser utilizadas para correta medida dos diâmetros.	IIb

## TRATAMENTO

### Tratamento clínico, indicações de tratamento cirúrgico, aspectos regulatórios, escolha de dispositivos

#### Tratamento clínico

O tratamento médico desse grupo de pacientes tem como principal objetivo retardar o crescimento, diminuir os riscos de ruptura e consequentemente impedir a necessidade de reparo cirúrgico ou endovascular. É importante realizar exames de imagem periódicos (por exemplo, eco-Doppler, angiotomografia) para monitorar o seu crescimento. Além disso, recomenda-se o controle estrito da pressão arterial, tratamento da dislipidemia, do diabetes, parar de fumar e avaliar outros fatores de risco para aterosclerose.

#### Controle da hipertensão

Está bem demonstrado que a hipertensão mal controlada está relacionada com o aumento da frequência de eventos cardiovasculares, infarto agudo do miocárdio (IAM) e acidente vascular cerebral (AVC), assim como o rápido crescimento do aneurisma ou dissecção da aorta. O uso de betabloqueadores e anti-hipertensivos (bloqueadores dos receptores da angiotensina II) com a manutenção da pressão sistólica abaixo de 130 mmHg e a diastólica abaixo de 80 mmHg, associados a estatinas, retarda o crescimento assim como minimiza a frequências de tais eventos.

De acordo com as Diretrizes do American College of Cardiology/American Heart Association (ACC/AHA)^82^ e da European Society of Cardiology (ESC)^11^, o tratamento dos AAAs com inibidores da enzima conversora de angiotensina (ECA) e betabloqueadores é considerado recomendação Classe IIa, ou seja, indica que os benefícios do tratamento superam os riscos e que o uso do tratamento é razoável. No entanto, a força das evidências que suportam o uso de ECA e betabloqueadores em AAA é considerada Nível de Evidência B, o que significa que as evidências vêm principalmente de estudos não randomizados ou registros observacionais^83-87^.

#### Uso de estatinas

Vários estudos investigaram o impacto da terapia com estatina no crescimento de aneurismas aórticos, com resultados mistos. Enquanto alguns estudos descobriram que a terapia com estatina pode retardar o crescimento de aneurismas aórticos ou reduzir o risco de ruptura, outros não encontraram nenhum impacto significativo.

A redução em 50% do LDL em pacientes menores que 75 anos mostrou uma redução do número de eventos cardiovasculares e AVC. Em outra metanálise ficou demonstrado que o uso de estatinas retardou o crescimento do aneurisma, consequência da ação sobre a matriz metaloproteinase-9 ou na concentração da interleucina-6. Essa redução tem efeito favorável sobre o processo de degeneração da camada média da parede da aorta, e, concomitante a isso, atua no curso da inflamação e da aterosclerose.

Uma metanálise de vários estudos publicados no Journal of Vascular Surgery em 2018 mostrou que a terapia com estatina está associada a uma redução significativa no risco de expansão do aneurisma e morte relacionada ao aneurisma, além de redução significativa na taxa de crescimento de aneurismas de aorta abdominal em pacientes submetidos a reparo^87^.

O uso de estatinas antes e ou depois do tratamento endovascular do AAA está associado a um de ganho de 5 anos de sobrevida quando comparados ao grupo que não usou estatina; no entanto, é importante observar que esses estudos têm algumas limitações e são necessárias mais pesquisas para entender completamente o impacto da terapia com estatina no crescimento do aneurisma da aorta. Também é importante ressaltar que a terapia com estatina não é um tratamento específico para aneurismas da aorta, e seu uso deve ser considerado como parte de uma abordagem abrangente para gerenciar os fatores de risco associados aos aneurismas da aorta^88-92^.

#### Interrupção do hábito de fumar

A interrupção do hábito de fumar é considerada uma recomendação importante para o tratamento dos AAAs, pois tem forte relação com o crescimento e ruptura do aneurisma. O tabagismo é um importante fator de risco para o desenvolvimento de AAA e está associado a uma maior incidência de crescimento e ruptura de aneurismas.

As Diretrizes do ACC/AHA^82^ e da ESC^11^ para o manejo de AAA indicam a cessação do tabagismo como recomendação Classe I. O nível de evidência para cessação do tabagismo no manejo do AAA é considerado Nível de Evidência B, o que significa que a evidência vem principalmente de estudos não randomizados ou registros observacionais. Esses estudos mostraram que os fumantes têm um risco muito maior de desenvolver AAA e de ruptura do aneurisma do que os não fumantes.

Foi demonstrado que parar de fumar reduz o risco de crescimento e ruptura de aneurismas, diminuindo a pressão sobre o aneurisma, reduzindo a inflamação e o estresse oxidativo na aorta e melhorando a saúde da parede aórtica. Além disso, parar de fumar reduz o risco de outras doenças cardiovasculares, IAM, AVC e doença arterial periférica. Portanto, os profissionais de saúde devem encorajar fortemente seus pacientes com AAA a parar de fumar e, se necessário, encaminhá-los para programas de cessação do tabagismo. Fumar é um fator de risco independente para o desenvolvimento, crescimento e complicações do AAA, além de aumentar a morbimortalidade do reparo cirúrgico ou endovascular^93-95^.

#### Uso de antiagregantes

A terapia antiplaquetária, como aspirina e o clopidogrel, é considerada um aspecto importante do manejo dos AAAs, pois é usada para reduzir o risco de eventos cardiovasculares. As Diretrizes do ACC/AHA^82^ e da ESC^11^ para o manejo do AAA recomendam o uso de terapia antiplaquetária para todos os pacientes com AAA, a menos que haja contraindicação. O uso de terapia antiplaquetária, como por exemplo o ácido acetilsalicílico na dose de 75-162 mg dia, diminui os riscos de eventos cardiovasculares nesse grupo de pacientes, considerado recomendação Classe I, indicando que os benefícios do tratamento superam os riscos e que o tratamento deve ser realizado.

Porém, quando avaliamos a velocidade de crescimento, os dados são controversos. Alguns trabalhos mostram que no grupo de pacientes portadores de AAA maior que 4 cm de diâmetro observou-se uma diminuição da velocidade de crescimento do aneurisma. Por outro lado, um estudo da Dinamarca demonstrou um aumento da mortalidade na vigência de ruptura no grupo de pacientes que utilizava antiagregante, quando comparado ao grupo que não utilizava^96,97^.

#### Triagem e vigilância

O Aneurysm Detection and Management Study (ADAM) é um estudo multicêntrico, randomizado e controlado, que teve como objetivo determinar a melhor estratégia para detectar e tratar AAAs. O estudo comparou duas estratégias: uma, envolvia triagem única com ultrassom, seguida de vigilância daqueles com aneurismas com diâmetro maior ou igual a 3 cm; e a outra, envolvia nenhuma triagem e tratamento de AAAs apenas quando eram encontrados incidentalmente ou quando causaram sintomas^98^.

Os resultados do estudo mostraram que a triagem única com ultrassonografia seguida de vigilância daqueles com aneurismas com diâmetro maior ou igual a 3 cm foi associada a uma redução significativa no número de mortes por AAA, em comparação com a estratégia sem rastreio. A classe de recomendação para triagem única com ultrassonografia seguida de vigilância daqueles com aneurismas com diâmetro maior ou igual a 3 cm é I, e o nível de evidência é A. Na Tabela 4 encontram-se resumidas as recomendações de seguimento e tratamento clínico.

**Tabela 4:** Recomendações de seguimento e tratamento clínico para pacientes portadores de aneurisma da aorta abdominal

Recomendação	Nível de evidência
Considerar associação de betabloqueadores e/ou inibidores da angiotensina II com o objetivo de manter a pressão arterial abaixo de 130 *versus* 80 mmHg. Considerar associação de estatinas para retardar o crescimento e diminuir a incidência de eventos cardiovasculares.	IIa
Recomenda-se cessação do tabagismo para todo paciente com diagnóstico de aneurisma de aorta abdominal.	IIa
Recomenda-se terapia antiagregante plaquetária em todo paciente com diagnóstico de aneurisma de aorta abdominal, salvo expressa contraindicação.	IIa
Recomenda-se seguimento regular com ultrassonografia de abdome para os pacientes com diâmetro da aorta superior a 3 cm.	I

### Indicação de tratamento cirúrgico e endovascular

Atualmente, a maioria das diretrizes internacionais recomenda o tratamento endovascular (EVAR) como o tratamento preferencial para a maioria dos pacientes com AAA, mas o reparo cirúrgico aberto (RA) ainda é recomendado para alguns pacientes, principalmente para aqueles que não são adequados para EVAR devido à extensão, localização e anatomia do aneurisma. As indicações de correção dos AAA, tanto pelo método cirúrgico quanto endovascular, podem ser descritas, de forma simplificada, como:

- Aneurisma fusiforme com diâmetro maior que 5 cm para mulheres e 5,5 cm para homens;
- Aneurisma fusiforme com crescimento rápido, sendo maior que 0,5 cm em 6 meses ou maior que 1cm em 1 ano;
- AAA associado a complicações e/ou sintomas;
- Formato da dilatação: aneurisma com formato do tipo sacular.

No entanto, existem importantes considerações a serem feitas a respeito das indicações de tratamento, que serão discutidas nos parágrafos que seguem.

## DIÂMETRO

Muito embora ter como base da indicação de tratamento somente o critério do maior diâmetro do aneurisma seja controverso, esse critério ainda continua sendo o mais utilizado. Em função disso, atualmente, o limite de 5 cm de diâmetro para mulheres e 5,5 cm para homens é, atualmente, o critério mais encontrado na literatura para a indicação do tratamento cirúrgico ou endovascular. No entanto, existe alguma controvérsia na literatura e a recomendação pode variar, dependendo da localização do aneurisma, da condição clínica e dos fatores de risco individuais do paciente.

O uso de EVAR em AAAs pequenos e assintomáticos ainda é um tópico de discussão e investigação, e as recomendações podem mudar à medida que novas evidências surgirem. Diante da expressiva diminuição da morbimortalidade cirúrgica obtida com a utilização da técnica endovascular, o reparo precoce dos aneurismas considerados pequenos (4-5 cm) voltou a ser tema de discussão recorrente. No entanto, os dados atualmente disponíveis demonstram que não existe nenhuma evidência de benefício no tratamento precoce dos aneurismas pequenos quando comparamos com o tratamento conservador. Os estudos UK Small Aneurysm Trial, ADAM e Comparison of surveillance versus aortic endografting for small aneurysm repair (CAESAR): results from a randomised trial^99-101^ corroboram essas premissas – muito embora entre os grupos comparados nos três estudos exista um número expressivo de *crossover* e também a demonstração que um de cada seis aneurismas em acompanhamento clínico perde as características anatômicas ideais para o reparo endovascular^98-102^. Se estudos randomizados de aneurismas pequenos não mostraram até o momento evidência de eficácia para reparos abaixo de 5,5 cm, isso não significa que a eficiência não possa ser melhor^103^. Dados como a demora do encaminhamento, as condições de acesso ao sistema de saúde e a mortalidade apresentada pelo serviço que vai fazer a intervenção pesam sobre esses números. Portanto, é plausível admitir que com um tamanho entre 5-5,5 cm uma determinada intervenção possa ser justificada^6,102,103^.

Para aneurismas menores de 4 cm de diâmetro, sem crescimento ou sintomas significativos, geralmente é recomendado um monitoramento rigoroso com exames de imagem. Para aneurismas de 4-5 cm de diâmetro, sem crescimento ou sintomas significativos, recomenda-se monitoramento rigoroso com exames de imagem. O EVAR pode ser considerado para aqueles com crescimento significativo ou se houver outros fatores de risco para rotura.

## CRESCIMENTO RÁPIDO

Dois dos fatores relacionados à rotura de aneurismas são diâmetro e taxa de crescimento rápida. A média de crescimento anual dos AAAs é aproximadamente 0,26 cm ao ano naqueles menores que 5 cm, passando para 0,5 cm ao ano nos aneurismas maiores que 5 cm. Dessa forma, o crescimento mais acelerado, ou seja, maior que 0,5 cm em 6 meses^82^ ou 1 cm em 1 ano^6,82^, é considerado critério primário para o reparo eletivo dos AAAs.

## PRESENÇA DE SINTOMAS

Os AAAs são tipicamente assintomáticos e frequentemente encontrados incidentalmente durante estudos de imagem para outras condições. Vale a pena notar que os sintomas de um AAA podem ser sutis e, muitas vezes, pouco perceptíveis. No entanto, à medida que o aneurisma aumenta, pode causar sintomas como:

- Dor abdominal ou nas costas (lombar);
- Dor lombar com irradiação para região inguinal;
- Sensação de plenitude ou desconforto no abdome;
- Sensação de pulsação no abdome.

A análise multivariada de alguns estudos identificou como os maiores preditores de risco para ruptura a expansão rápida documentada e a presença de dor abdominal ou lombar significante, independentemente do tamanho do AAA^104-107^.

## FORMATO SACULAR

São considerados saculares aqueles aneurismas cuja alteração ou deformidade ocorre em apenas uma das faces da aorta e determina uma dilatação focal em formato de saculação. Os aneurismas do tipo sacular são menos frequentes, e os dados sobre sua evolução natural são escassos. Conforme alguns estudos, parecem ser mais comuns em mulheres e, quando comparamos aos fusiformes, os sintomas se manifestam em diâmetros menores. De forma geral, o tratamento endovascular ou cirúrgico está indicado nos aneurismas de formato sacular, mesmo que ainda exista falta de dados e consenso sobre o tamanho dos mesmos.

Em geral, o nível de evidência para o tratamento dos aneurismas saculares é considerado de moderado a baixo. Isso se deve à falta de ensaios clínicos randomizados de boa qualidade focados nesse tipo de aneurisma. A maioria das evidências disponíveis vem de estudos observacionais e séries de casos, que podem fornecer informações úteis, mas são considerados menos confiáveis do que os ensaios clínicos randomizados. Por exemplo, a Society for Vascular Surgery recomenda o reparo endovascular para aneurismas saculares menores que 5 cm de diâmetro, mas essa é considerada uma recomendação fraca^7^. Isso ocorre porque as evidências que sustentam essa recomendação são de baixa qualidade, e a decisão de tratar deve ser baseada na consideração das características e preferências individuais do paciente. É importante ter em mente que as recomendações de tratamento para aneurismas saculares podem variar, dependendo das diretrizes ou fontes específicas às quais se está referindo, e são baseadas no conhecimento e compreensão atuais da condição^108,109^.

A Tabela 5 traz as recomendações para reparo cirúrgico eletivo do AAA.

**Tabela 5:** Recomendações de reparo cirúrgico eletivo em pacientes portadores de aneurisma de aorta abdominal assintomáticos

Recomendação	Nível de evidência
Recomenda-se intervenção cirúrgica convencional ou endovascular para aneurismas fusiformes da aorta acima de 5 cm de diâmetro em mulheres e de 5,5 cm em homens.	I
O reparo eletivo do aneurisma da aorta abdominal pode ter justificativa nos diâmetros entre 5-5,5 cm, com base nas condições clínicas e anatômicas do paciente, acesso ao sistema de saúde e mortalidade do serviço de atendimento hospitalar.	IIb
Recomenda-se intervenção cirúrgica convencional ou endovascular para aneurismas fusiformes da aorta com crescimento acima de 5 cm por ano.	Ib
Recomenda-se intervenção cirúrgica convencional ou endovascular para aneurismas fusiformes da aorta sintomáticos.	IIb
Recomenda-se intervenção cirúrgica convencional ou endovascular para aneurismas saculares da aorta com qualquer diâmetro.	IIb

### Cirurgia convencional e endovascular

Os AAAs podem ser tratados com dois tipos de procedimentos: EVAR e RA. Durante mais de 50 anos o procedimento cirúrgico com a interposição de um enxerto reto ou bifurcado foi utilizado no reparo dos AAAs como primeira opção de tratamento. No entanto, com o desenvolvimento de técnicas endovasculares, essa estratégia passou a ser substituída por procedimentos minimamente invasivos. Apesar de toda a tecnologia empregada sempre houve controvérsias sobre a superioridade de uma ou outra técnica no que se refere aos resultados a curto, médio e longo prazo. Atualmente, é de consenso na literatura que em condições iguais de risco, ou seja, pacientes com mesmas características clínicas e com anatomia adequada para a técnica endovascular, ambas as abordagens poderão ser adotadas com resultados similares – portanto, a decisão da técnica a ser empregada será decidida em consenso entre equipe e paciente. Para isso, há dados na literatura com classe de recomendação I e qualidade nível A^110,111^.

A escolha de qual procedimento usar depende de vários fatores, incluindo idade do paciente, fatores de riscos e tamanho e localização do aneurisma. O médico discutirá sobre qual a melhor opção depois de avaliar o histórico médico e a condição clínica do paciente. Existem vários estudos e diretrizes internacionais que são utilizados para orientar o tratamento de pacientes com AAAs^6,7,12,82^.

É necessário também considerar a Diretriz Brasileira para o Tratamento do Aneurisma da Aorta Abdominal publicada em agosto de 2016 pela Comissão Nacional de Incorporação de Tecnologias para o Sistema Único de Saúde^112^. Baseada em uma cuidadosa revisão bibliográfica e análise dos resultados obtidos com o tratamento endovascular do tratamento do AAA, comparado ao tratamento por cirurgia convencional nos hospitais de referência do Sistema Único de Saúde (SUS), essa diretriz recomenda fortemente o uso da técnica endovascular no tratamento dos AAAs, reservando o RA somente na impossibilidade da realização do EVAR.

Os resultados a longo prazo podem ser influenciados por muitos fatores, relacionados com características do paciente, qualidade do reparo e extensão do acompanhamento. Além disso, estudos de longo prazo também mostraram que a durabilidade das endopróteses usadas no EVAR pode ser limitada, principalmente aquelas de primeira geração, e reintervenções podem ser necessárias no futuro para substituir, complementar ou corrigir migrações da endoprótese. Importante ressaltar mais uma vez que as taxas de reintervenções e eventos adversos são geralmente mais frequentes no EVAR do que no RA, mas, ainda assim, as taxas de mortalidade são menores.

Atualmente, a maioria das diretrizes internacionais e dos estudos randomizados recomenda o EVAR como o tratamento preferencial para a maioria dos pacientes com AAA. Todos esses estudos são considerados de alta qualidade e fornecem fortes evidências para o uso de EVAR, mas o RA ainda é recomendado para alguns pacientes, particularmente aqueles que não são adequados para a técnica endovascular devido à extensão, localização ou anatomia do aneurisma.

#### Tomada de decisão

O processo de tomada de decisão para o tratamento de um AAA deve ser individualizado com base nas características do paciente. Trata-se de uma decisão que deve ser discutida em uma equipe multidisciplinar, que envolve um cirurgião vascular e outros especialistas, como intervencionistas e cardiologistas. A abordagem da equipe multidisciplinar permite a consideração de vários fatores que podem influenciar a escolha do tratamento. Essa abordagem é fundamental na adequação do paciente para diferentes opções de tratamento e a consideração dos resultados a longo prazo e possíveis complicações de cada opção. A equipe multidisciplinar permite a integração de diferentes perspectivas e conhecimentos, o que pode levar a uma tomada de decisão mais precisa e abrangente e a melhores resultados para o paciente. A abordagem em equipe também permite o manejo de possíveis complicações e o acompanhamento do paciente de forma coordenada, o que pode melhorar a continuidade do cuidado. A decisão médica compartilhada, aspecto muito discutido na tomada de decisão da atualidade, onde o paciente está no centro do processo decisório, deve ainda ser trabalhada na abordagem do AAA.

#### A adesão às instruções de uso

As IFUs são mandatórias no reparo endovascular de aneurisma para garantir a segurança e eficácia do produto e do procedimento. Elas fornecem informações sobre as indicações, contraindicações, advertências, precauções e instruções para o uso do dispositivo endovascular, como as endopróteses utilizadas no EVAR. O não cumprimento dessas IFUs pode levar a complicações, como falha do dispositivo, vazamentos (*endoleaks*), migração do dispositivo, entre outras. Em alguns casos, essas complicações podem requerer reintervenções ou conversão para RA. Dados da literatura mostram, contudo, que cerca de 42% dos casos seguem recomendações mais conservadoras de IFU, e 69% seguem recomendações mais liberais, enquanto os demais estão fora das recomendações de uso^13^.

A adesão às IFU inclui seguir a técnica de implantação adequada, garantir o correto dimensionamento e posicionamento do dispositivo e monitoramento das condições do paciente durante e após o procedimento para detectar possíveis complicações. Também é importante observar que a IFU pode mudar ao longo do tempo conforme novas informações ou atualizações forem disponibilizadas, por isso é importante manter-se atualizado com a versão mais recente da IFU e consultar o fabricante para obter informações adicionais.

### Tratamento convencional aberto

#### Indicações do reparo aberto

As indicações para o RA dos AAAs incluem os aneurismas assintomáticos e aqueles com rápida expansão, embolização distal ou ruptura, e não diferem daquelas estabelecidas para o EVAR. Por outro lado, o RA é a técnica indicada na maioria dos aneurismas infectados ou quando há necessidade de conversão após um reparo endovascular mal-sucedido.

Nas principais contraindicações relativas ao RA estão incluídos os casos de abdome hostil, comorbidades (especialmente cardíacas e renais) e curta expectativa de vida^7,38,113^.

#### Técnica do reparo aberto

Os passos da técnica para o RA não mudaram muito nas últimas 7 décadas. A incisão mais utilizada é a xifo-pubiana e o retroperitônio é abordado após rotação visceral para a direita. A dissecção do retroperitônio se faz desde o colo proximal do aneurisma até as artérias ilíacas comuns ou bifurcação dessas, dependendo da anatomia do paciente. Após heparinização sistêmica, se procede o pinçamento da aorta e ilíacas, abertura do saco aneurismático e ligadura das lombares. Um enxerto reto ou bifurcado é então interposto; as anastomoses termino-terminais são então confeccionadas com sutura contínua. Um anel de *teflon* pode ser utilizado como reforço da anastomose proximal em casos selecionados. Após liberação das pinças vasculares, as anastomoses e os sítios de ligadura das lombares são inspecionados no intuito de surpreender algum sangramento. O saco aneurismático é então aproximado sobre o enxerto e se procede a laparossíntese.

#### Preservação do fluxo pélvico e visceral

A perfusão do fluxo pélvico e visceral depende das comunicações entre a mesentérica superior, mesentérica inferior e artérias hipogástricas. A obstrução das artérias hipogástricas pode levar à disfunção erétil, claudicação de nádegas e mais raramente à isquemia colônica e medular. Durante o RA, todos os esforços devem ser direcionados para preservar o fluxo de pelo menos uma das hipogástricas.

O risco de isquemia do cólon aumenta consideravelmente quando a revascularização exclui ambas as hipogástricas (derivação aorto-femoral bilateral com exclusão das hipogástricas)^6,7^. Em estudo prospectivo canadense, o risco de isquemia do cólon aumentou em oito vezes quando ambas as ilíacas eram excluídas (de 0,3 para 2,6%)^114^.

O reimplante da artéria mesentérica inferior no enxerto como técnica profilática da isquemia do cólon tem resultados conflitantes. É razoável considerar seu reimplante em situações muito específicas, como colectomia prévia, interrupção de colaterais entre a artéria mesentérica superior (AMS) e artéria mesentérica inferior (AMI), oclusão e/ou estenose da AMS ou ausência da arcada de Riolan^115,116^. O estudo prospectivo randomizado de 2006 concluiu que o reimplante da AMI seria benéfico em pacientes idosos ou naqueles em que havia sangramento intraoperatório considerável^117^. O estudo prospectivo canadense^114^ observou que nos pacientes submetidos a reimplante (5% da série) o sangramento pós-operatório era mais frequente.

#### Tipo de incisão

##### Incisão transversa versus mediana

Um estudo prospectivo randomizado de 2005 avaliou pacientes selecionados considerando incisão transversa *versus* longitudinal^118^. A regressão logística após mais de 4 anos demonstrou, em um pequeno grupo de 69 pacientes, uma incidência de hérnia incisional maior no grupo incisão mediana (p = 0,010). É razoável indicar a incisão transversa nos pacientes com DPOC, já que a restrição respiratória é menor^7^.

##### Incisão mediana versus retroperitonial

Mesmo os estudos randomizados que compararam a incisão mediana *versus* retroperitonial apresentam resultados conflitantes^119-121^. Apenas duas variáveis mensuráveis – menor tempo de íleo adinâmico e alimentação mais precoce – favoreceram a incisão retroperitonial. A exposição limitada da artéria renal direita e da artéria ilíaca direita são desvantagens da incisão retroperitonial. No estudo prospectivo e randomizado de Sieunarine et al.^119^ não foram verificadas diferenças significativas entre as duas incisões, com exceção de abaulamento na incisão (*bulge*) e mais intensa e prolongada dor em desfavor da incisão retroperitonial. Por outro lado, a incisão longitudinal se associou a maior frequência de hérnias. Parece claro que a incisão retroperitoneal deve ser reservada para os casos de abdome hostil e casos selecionados de aneurismas inflamatórios^122^ e de rim em ferradura.

##### Reforço com tela de marlex na incisão mediana

Um estudo randomizado^123^ e uma metanálise de 2018^124^ demonstraram que o uso profilático de reforço com tela reduz o risco de hérnia incisional nas incisões xifo-pubianas. Entretanto, não há acompanhamento a longo prazo desses grupos ou relato do número de reintervenções para uma análise mais adequada. Na Tabela 6, encontram-se em destaque as recomendações referentes ao RA dos AAAs.

**Tabela 6:** Recomendações referentes ao reparo aberto dos aneurismas abdominais

Recomendação	Nível de evidência
Recomenda-se preservação do fluxo para pelo menos uma das ilíacas internas, tanto na técnica aberta quanto na endovascular.	I
Recomenda-se incisão transversa nos pacientes com doença pulmonar obstrutiva crônica.	IIb
Recomenda-se incisão retroperitonial nos casos em que há rim em ferradura, aneurisma inflamatório e abdome hostil.	IIa

#### Aneurisma justarrenal

Por definição, o aneurisma justarrenal se caracteriza pela presença de colo proximal curto o suficiente para exigir o pinçamento suprarrenal na correção aberta, mas que, anatomicamente, não compromete a aorta ao nível das artérias renais^125^. Cerca de 15% dos AAAs são classificados como justarrenais^125^. Várias séries clínicas não definem adequadamente essa situação anatômica, incluem os aneurismas pararrenais e não demonstram os resultados a longo prazo.

Não há estudos randomizados e controlados na literatura até o momento comparando RA com EVAR, sendo essa é a principal dificuldade em comparar as duas técnicas. É importante perceber que os reparos endovasculares ramificados (REVAR) e fenestrados (FEVAR), com baixas taxas de complicações, são realizados em centros altamente experientes e com baixas taxas de mortalidade. A decisão por operar aberto ou pelo método endovascular um aneurisma justarrenal é certamente multifatorial. Este documento não tem, portanto, a intenção de comparar as duas técnicas.

A baixa mortalidade do RA para o aneurisma justarrenal pode constatada em várias séries clínicas e metanálises. Uma metanálise de 2010 avaliou os resultados do RA e incluiu 1.256 pacientes de 21 estudos não randomizados em uma busca na MEDLINE, EMBASE e Cochrane. A mortalidade perioperatória foi de 2,9% (IC95% 1,8-4,6) e necessidade de diálise em 3,3% (IC95% 2,4-4,5)^126^. Em uma análise retrospectiva mais recente de cinco centros acadêmicos franceses^127^, incluindo 315 pacientes consecutivos, também se demonstrou uma baixa mortalidade do RA (0,9%).

Com o advento da correção endovascular com endopróteses fenestradas^128,129^ ramificadas ou com stents paralelos, várias séries clínicas e algumas metanálises foram publicadas comparando o RA com o reparo endovascular. Uma metanálise de 2022 confirmou os excelentes resultados das endopróteses fenestradas no tratamento dos aneurismas justarrenais^130^, porém sem redução significativa da mortalidade, comparados com os resultados do tratamento endovascular, sendo que esse, apesar da menor morbidade, se associou a maior número de reintervenções. Nesse mesmo ano foi publicada a metanálise de Dooman et al.^131^, que incluía na pesquisa os aneurismas pararenais e várias técnicas endovasculares. Essa revisão sistemática mostrava menor mortalidade para o EVAR. Uma metanálise mais recente (UK-COMPASS) feita com mais de 7.000 pacientes comparou o RA com várias modalidades de reparo endovascular^132^, que incluíam o uso de endopróteses convencionais fora das IFU, stents paralelos e endopróteses fenestradas. Foi observada uma menor mortalidade para o EVAR, mas essa diferença era perdida em 30 meses.

Um estudo comparativo em pacientes idosos mostrou que não parece haver diferença entre RA e endopróteses fenestradas em relação à mortalidade do procedimento^133^, mas é necessário lembrar que a comparação não é possível, e, mesmo com pacientes pareados, as diferenças anatômicas, critérios de seleção de pacientes, diferença da experiência das equipes, tanto para o RA quanto para o FEVAR e REVAR, dificultam este tipo de análise.

As dificuldades óbvias para uma indicação mais ampla das endopróteses fenestradas se relaciona com o seu alto custo, baixa disponibilidade e necessidade de um treinamento adequado nas técnicas endovasculares. Por outro lado, o RA dos aneurismas justarrenais impõe dificuldades técnicas e exige uma *expertise* adicional da equipe cirúrgica e cuidados intensivos e qualificados no pós-operatório.

O RA deve preferencialmente ser realizado por equipes que tratam AAA com mortalidade hospitalar inferior a 5%, e depende do número anual de cirurgias no respectivo centro^134^. Nessa condição, o RE deve ficar reservado para os pacientes com comorbidades consideráveis e/ou em centros com grande volume de procedimentos endovasculares.

Na Tabela 7, encontram-se resumidas as indicações de RA e endovascular para os aneurismas justarrenais.

**Tabela 7:** Recomendações referentes ao reparo dos aneurismas justarrenais

Recomendação	Nível de evidência
Recomenda-se cirurgia aberta para aneurismas justarrenais nos centros com mortalidade inferior a 5%.	IIb

### Tratamento endovascular do aneurisma infrarrenal

O EVAR se confirmou como importante avanço técnico e vem se tornando como primeira opção terapêutica em diversos países. O principal objetivo do EVAR é conseguir o selo proximal e distal, impedindo o contato do sangue com a parede aneurismática e finalmente prevenindo a rotura. Desde o primeiro implante no mundo, e no Brasil em 1994, muitas endopróteses sofreram modificações em relação a diâmetro, material, mecanismos de fixação proximal e navegabilidade, entre outros. Muitas limitações anatômicas foram superadas e centenas de cirurgiões vasculares receberam treinamento na técnica, atualmente disponível amplamente no território nacional, no sistema de saúde suplementar e também no SUS. As IFUs podem variar de acordo com o fabricante e recomenda-se fortemente verificar junto à bula do produto quais são os critérios anatômicos necessários para uso do material.

Existe uma série de estudos randomizados e controlados sobre o EVAR. Um dos estudos mais importantes é o Randomized Controlled Trial of Endovascular Repair versus Open Repair for Abdominal Aortic Aneurysm (EVAR 1)^135^. Esse ensaio demonstrou que o EVAR estava associado a um menor risco de morte a curto prazo em comparação com o RA. Outro importante estudo é o Endovascular Aneurysm Repair (EVAR 2), onde pacientes considerados não candidatos ao RA receberam EVAR e não houve melhora dos índices de mortalidade, com aumento da necessidade de reintervenções^136^.

O DREAM é outro estudo randomizado que encontrou menor mortalidade perioperatória para o grupo EVAR^137^. Assim como os estudos anteriores, também mostrou que o EVAR estava associado a um menor risco de morte e menor risco de complicações em comparação com o RA, apesar de maior número de reintervenções^138^. Essa vantagem fica bem evidente a favor do EVAR durante os primeiros 6 meses, mas não se sustenta a longo prazo. Assim, mesmo alguns estudos de longo prazo, como o Open versus Endovascular Repair of Abdominal Aortic Aneurysm (OVER), mostraram que a sobrevida após um longo período de seguimento (4-8 anos) foi similar entre os dois grupos^139^.

Em 2008, metanálise de estudos randomizados e observacionais que incluiu 42 estudos totalizando 21.178 pacientes, compara o RA com o EVAR. Em procedimentos eletivos, observou-se menor tempo de cirurgia, menor perda sanguínea, menor mortalidade em 30 dias, menor tempo de internação, menor tempo de permanência em unidade de cuidados intensivos e menos complicações cardíacas e respiratórias nos pacientes submetidos ao EVAR. Os autores recomendam que o EVAR seja a opção de escolha para pacientes com anatomia adequada, tanto nas cirurgias eletivas quanto nas urgências^140^.

Sajid et al.^141^, 2008 em metanálise incluindo três estudos randomizados que comparam o EVAR ao RA (total de 1.468 pacientes), confirmaram que o EVAR está́ associado à redução da mortalidade operatória, da dor pós-operatória, do tempo de permanência em UTI e do tempo de internação hospitalar. Os autores concluíram que o EVAR deve ser recomendado como tratamento de escolha em pacientes idosos e de alto risco. Revisão da Cochrane, publicada em 2014, comparou o EVAR ao RA no reparo de AAA. Foram incluídos cinco estudos, que encontraram diferença estatística a favor de EVAR em relação à mortalidade de curto prazo, embora nenhuma diferença tenha sido encontrada a médio e longo prazo. A taxa de reintervenção no grupo EVAR foi estatisticamente maior em relação à cirurgia aberta, embora tal resultado deva ser interpretado com cautela devido à heterogeneidade entre os estudos. A maioria das reintervenções foi por técnica endovascular e associada à baixa mortalidade^142^.

As evidências cada vez mais apontam que a tomada de decisão deve ser compartilhada com o paciente, especialmente com aqueles de altíssimo risco cirúrgico e frágeis, para os quais possivelmente o aconselhamento deva ser na direção de não operar. Para todos os outros com condições anatômicas e risco cirúrgico bom ou moderado, o EVAR e o RA podem ser propostos como solução terapêutica.

Na Tabela 8, encontram-se as recomendações referentes ao EVAR do AAA.

**Tabela 8:** Recomendações referentes ao tratamento endovascular dos aneurismas abdominais

Recomendação	Nível de evidência
Recomenda-se reparo endovascular como modalidade de tratamento preferencial na presença de equipe treinada e condição anatômica favorável.	IIa
Recomenda-se reparo aberto para pacientes jovens e baixo risco clínico e cirúrgico.	IIa
Recomenda-se acesso e fechamento percutâneo guiado por ultrassom no lugar de acesso cirúrgico convencional para reduzir o tempo operatório, perda de sangue, duração da internação, tempo de cicatrização da ferida e dor.	IIb

## ESCOLHA DO ACESSO PARA EVAR

A maior disponibilidade de dispositivos de fechamento percutâneo e endopróteses de baixo perfil tornaram o acesso percutâneo guiado por ultrassom e o fechamento mais viáveis. Dois estudos randomizados^143,144^ e uma grande revisão retrospectiva mostraram resultados favoráveis do acesso e fechamento percutâneo da artéria femoral comum, como redução do tempo operatório, redução da perda de sangue e melhores resultados centrados no paciente, como redução da dor.

O estudo PEVAR mostrou a não inferioridade do acesso percutâneo total e fechamento para EVAR para pacientes com anatomia adequada da artéria femoral comum^143^. No estudo PiERO (acesso femoral percutâneo no reparo endovascular *versus* acesso femoral aberto), observou-se menos dor e menor tempo de cicatrização nos pacientes submetidos a acesso percutâneo em comparação com os pacientes submetidos a acesso cirúrgico na virilha^144^. Não se observou diferença na incidência de infecção no local do acesso entre os grupos. Além disso, um estudo observacional multicêntrico de acesso à artéria femoral comum mostrou uma diminuição significativa nos hematomas na virilha com acesso guiado por ultrassom de rotina^145^.

Em pacientes submetidos à correção endovascular de AAA com anatomia adequada da artéria femoral comum, recomenda-se acesso e fechamento percutâneo guiado por ultrassom em vez de acesso cirúrgico, para reduzir o tempo operatório, perda de sangue, duração da internação, tempo de cicatrização da ferida e dor^82^.

Há uma recomendação relacionada ao acesso para EVAR na Tabela 8.

### Tratamento endovascular do aneurisma justarrenal

O EVAR apresenta menor taxa de mortalidade, tempo operatório e tempo de hospitalização do que o RA em curto e médio prazo, sendo o método de tratamento mais utilizado atualmente^135,137,146^. Porém, todas as endopróteses disponíveis comercialmente têm critérios de inclusão definidos nas IFUs. Essas instruções determinam os requisitos anatômicos mínimos para o uso desses dispositivos. Na maioria das endopróteses, o comprimento mínimo do colo proximal é de 1-1,5 cm, com angulação máxima de 45-60^o^, ausência de calcificação ou trombo circunferencial^147^.

Apesar dessas recomendações expressas nas instruções de uso dos dispositivos, um estudo retrospectivo de base de dados de pacientes submetidos à correção endovascular mostrou que somente 42% apresentavam anatomia dentro dos critérios mais conservadores, e 69% dentro dos critérios mais liberais^13^. No seguimento desses pacientes houve crescimento do saco aneurismático > 0,5 cm em 41% dos casos. Os fatores de risco associados ao crescimento foram: presença de *endoleak*, idade maior que 80 anos, diâmetro do colo proximal > 2,8 cm, angulação > 60^o^ e diâmetro da ilíaca comum > 2 cm.

A presença de colo hostil, principalmente com comprimento curto, cônico invertido ou irregular, eleva o risco de desenvolvimento de *endoleak* em quatro vezes, e de morte relacionada ao aneurisma em nove vezes em 1 ano^148^. Quando se somam critérios de colo hostil e é realizado implante em anatomias *off-label*, ou seja, fora das IFUs, aumenta a necessidade de procedimentos adjuntos, presença de *endoleaks* intraoperatórios e mortalidade geral^149^.

Portanto, o tratamento endovascular dos aneurismas com colo curto, justa ou pararrenais, exige técnicas mais avançadas do que o EVAR convencional, para assegurar uma zona de selamento adequada, com alta taxa de sucesso técnico, mas também para assegurar um reparo duradouro.

### Stents em paralelo

Uma dessas técnicas é a de stents em paralelo, seja na configuração de chaminé, periscópio ou sanduíche^150-152^. Essa técnica tem a vantagem de não usar dispositivos customizados, que levam tempo para serem produzidos, porém tem a desvantagem do risco de formação de “goteiras” que podem levar a *endoleaks*.

Vários estudos a respeito dessa técnica são registros e não estudos prospectivos, têm viés de seleção de pacientes, definição, padronização da técnica, avaliação de perviedade e seguimento de longo prazo^150^. A maior parte dos dados vem do registro PERICLES, em que 95% de 517 pacientes tinham aneurisma justarrenal^153^. A mortalidade de 30 dias em casos eletivos foi de 3,7%. A incidência de disfunção renal transitória foi de 28%, com necessidade de diálise permanente de 3%. O sucesso técnico foi de 97,1%, e 2,9% dos pacientes apresentaram *endoleak* persistente. A sobrevida global em seguimento de 17 meses foi de 79%. A perviedade das chaminés nos pacientes que tinham exame de imagem disponível foi de 94%. A média de regressão do saco aneurismático foi de 0,44 cm, apesar de não ser citada a proporção de pacientes onde houve aumento.

Em uma revisão sistemática da literatura, a incidência de *endoleaks* foi de 7,6% em comparação com 3,7% do reparo endovascular com FEVAR^154^. Os melhores resultados com stents paralelos são obtidos em pacientes onde é possível criar uma zona de selamento ≥ 1,5 cm, *oversizing* da endoprótese de 30% e no máximo duas chaminés^154,155^. Com o desenvolvimento das próteses fenestradas e ramificadas, o uso da técnica de stents em paralelo vem ficando cada vez mais reservado às situações de urgência, quando a anatomia não é adequada ao FEVAR ou como manobra de resgate, quando há oclusão inadvertida de ramos.

### Endoâncoras

Os dispositivos de endosuturas foram desenvolvidos para aumentar a fixação das endopróteses à parede aórtica, melhorando o alinhamento e prevenindo a migração ou *endoleak* IA em casos onde o EVAR convencional seria insuficiente, devido aos colos curtos ou angulados. Estudos experimentais mostram que o uso desses dispositivos aumenta a força necessária para o deslocamento entre a prótese e a parede aórtica, chegando próximo ou até ultrapassando aquela de uma sutura cirúrgica convencional^156^.

O uso desse dispositivo é relativamente simples, acrescenta uma média de apenas 17 minutos ao tempo operatório total^157^ e tem curva de aprendizado rápida.

No registro multicêntrico ANCHOR, em 208 casos foram aplicadas endoâncoras de forma profilática, em pacientes cuja anatomia os operadores julgassem como de alto risco para migração ou desenvolvimento de *endoleaks* IA. No total, 78,3% dos pacientes recrutados preenchiam critérios para colo hostil. O sucesso técnico foi de 95,2%. No seguimento médio de 14 meses, não houve rupturas, migrações ou conversão para cirurgia aberta. Nos pacientes submetidos a TC de controle, 1,5% apresentavam *endoleak* IA. O saco aneurismático > 0,5 cm diminuiu em 42,9% dos pacientes, enquanto em 1,6% houve aumento. As maiores limitações desse registro foram o seguimento incompleto e ausência de grupo controle^158^.

### Physician modified endo-grafts

Várias técnicas podem ser utilizadas para modificar uma endoprótese convencional em bancada, com a criação de fenestras, *scallops* ou ramos e assim incorporar artérias viscerais, criando uma zona de fixação em pacientes com anatomia inadequada para o implante de próteses convencionais. Essas técnicas têm a vantagem de não necessitar a espera para confecção de uma prótese customizada, sendo geralmente usadas em situações de urgência, em pacientes de alto risco cirúrgico, ou em instituições que não dispõem de próteses customizadas, quer seja no Brasil ou no mundo.

Com o desenvolvimento contínuo de próteses customizadas pela indústria, o uso de dispositivos modificados em situações eletivas tem ficado cada vez mais restrito. Em um estudo retrospectivo, Oderich et al. observaram uma mudança temporal, sendo que as próteses customizadas pelo cirurgião (PMEGs) foram mais utilizadas no início da experiência do tratamento endovascular de aneurismas justarrenais, pararrenais ou toracoabdominais, e as próteses customizadas (CMD) foram mais utilizadas no período mais recente. Nessa comparação, os pacientes tratados com PMEGs tinham mais comorbidades e aneurismas maiores. O sucesso técnico com próteses CMD foi maior (99,5% *versus* 98%, p = 0,02) e a mortalidade em 30 dias foi maior no grupo tratado com PMEGs (5,5% *versus* 0%, p = 0,0018). Em 3 anos de seguimento, a sobrevida, perviedade primária e secundária dos ramos foi semelhante nos dois grupos^159^. Recentemente, uma série grande de casos publicada com seguimento de 5 anos por um grupo bastante experiente com PMEGs mostrou boa perviedade e estabilidade dos ramos^160^.

### Endopróteses fenestradas ou ramificadas

A evolução técnica e aumento da experiência com reparo endovascular ofereceram a possibilidade de estender a zona de fixação proximal das endopróteses, incorporando as artérias viscerais e renais nos enxertos, permitindo o reparo de aneurismas justa e pararrenais^161^. A maior vantagem do reparo com próteses fenestradas/ramificadas (FEVAR/REVAR) em relação à cirurgia aberta é evitar o clampeamento aórtico e subsequente risco de disfunção renal, menor trauma cirúrgico e recuperação mais rápida, o que pode ser vantajoso para pacientes com alto risco cirúrgico. FEVAR/REVAR são técnicas desafiadoras, que devem ser desenvolvidas em centros especializados e devem ser realizadas por times cirúrgicos especializados e com grande experiência^6^.

Várias revisões sistemáticas mostraram a segurança e eficácia do FEVAR^162-165^. Em uma revisão de 14 séries de casos foram incluídos 751 pacientes^165^. A mortalidade intra-hospitalar ou em 30 dias foi de 4,1%. A prevalência de déficit renal transitório foi de 11%, com 2% de pacientes necessitando de diálise permanente. O registro GLOBALSTAR incluiu 318 pacientes tratados com FEVAR em 14 centros do Reino Unido^166^. A mortalidade perioperatória foi de 4,1%. Pacientes livres de reintervenção secundária foram 90%, 86% e 70% em 1, 2 e 3 anos, respectivamente.

Séries uni e multi-institucionais com endopróteses fenestradas e ramificadas mostraram resultados promissores^167,168^. Quando realizadas por cirurgiões experientes, o sucesso técnico foi alcançado em grande porcentagem dos casos (92-99,6%) com baixa mortalidade perioperatória. No seguimento de 1 ano, a perviedade dos ramos viscerais também se mostrou boa (96-98%) e, em 3 anos, 91% dos pacientes estavam livres de mortalidade associada ao aneurisma, com sobrevida global de 57%^169^.

Estudos observacionais recentes tiveram como objetivo comparar os resultados do tratamento dos aneurismas aórticos complexos entre RA e endovascular com FEVAR. Varkevisser et al. encontraram em um estudo comparativo entre FEVAR, RA para aneurisma aórtico complexo e EVAR um maior risco de óbito em 30 dias na correção cirúrgica aberta do que na correção endovascular (OR, 4,9; IC95% 1.4-1,9), e uma mortalidade equiparável a do EVAR^170^. É certo também que é necessário avaliar que o paciente que é o candidato a um reparo aórtico complexo como FEVAR ou REVAR já tem uma seleção por ser um candidato de risco bom ou moderado, uma vez que um paciente muito frágil ou de altíssimo risco pode não sobreviver a nenhum dos reparos, aberto ou endovascular.

Porém, a taxa de reintervenção tardia é maior após FEVAR, em comparação com a cirurgia aberta^165,171^, assim como as taxas de dano renal persistente e mortalidade em 3 anos (excluindo-se os óbitos perioperatórios) (HR 1,7; IC95% 1.1-2.6)^171^. Similar ao reparo endovascular infrarrenal, os dados disponíveis mostram achados semelhantes, com benefício inicial de sobrevida, porém com diminuição da vantagem ao longo do tempo e maiores taxas de reintervenção. Portanto, FEVAR pode ser mais benéfico para pacientes de moderado ou alto risco cirúrgico, que são mais propensos a apresentar complicações perioperatórias.

Além da questão da perviedade de ramos, outro ponto crítico a ser observado nos reparos FEVAR/BEVAR é a isquemia medular, notadamente associada a maiores extensões de cobertura aórtica. A confecção de próteses customizadas ou a modificação das próteses de prateleira disponíveis foram descritas como formas de reduzir o comprimento de cobertura aórtica^161,172^. Certamente outro ponto crítico a observar é o tempo de confecção de uma prótese sob medida, de modo que no momento é possível que as PMEGs e as próteses de prateleira continuarão a ter importante papel nos casos de urgência e nos aneurismas sintomáticos^160,173^.

Portanto, em paciente jovem com bom risco operatório, o RA é o mais recomendado, e naqueles onde há moderado ou alto risco cirúrgico, o reparo endovascular é o mais indicado, com preferência ao FEVAR/REVAR, desde que anatomicamente possível. As opções de tratamento devem ser discutidas com o paciente e familiares, levando-se em consideração as vantagens e desvantagens de cada técnica. Nos pacientes submetidos ao tratamento endovascular, deve ser feito acompanhamento rigoroso com exames anuais de imagem^167^.

Na Tabela 9, encontram-se resumidas as recomendações referentes ao tratamento endovascular de aneurismas justa e pararrenais.

**Tabela 9:** Recomendações referentes ao tratamento endovascular dos aneurismas de aorta justa e pararrenais

Recomendação	Nível de evidência
Em pacientes jovens e de bom risco clínico recomenda-se reparo aberto do aneurisma justa ou pararrenal acima dos 5,5 cm.	I
Em pacientes de moderado a alto risco cirúrgico e naqueles sintomáticos, considerar o reparo endovascular com uma das técnicas disponíveis.	IIa
Em pacientes frágeis e de altíssimo risco cirúrgico, considerar não tratar cirurgicamente, por via aberta ou endovascular, em decisão compartilhada.	IIa

## SEGUIMENTO PÓS-OPERATÓRIO

Após o tratamento dos AAAs, o objetivo do acompanhamento é evitar complicações ou mortes relacionadas ao aneurisma. Após o tratamento por cirurgia aberta, a formação de pseudoaneurismas junto às anastomoses ou a dilatação e formação de novos aneurismas da aorta ou artérias ilíacas é incomum no acompanhamento inicial, com taxas em torno de 1% em 5 anos, 6-12% em 10 anos e até 35% em 15 anos do pós-operatório^174-176^.

Nos pacientes submetidos ao tratamento endovascular, a taxa de complicações e a necessidade reintervenções é significativamente maior, e as complicações mais encontradas são os *endoleaks*. Podem também ser encontrados crescimento do saco aneurismático sem *endoleak* detectável, migração das endopróteses e falhas do material das endopróteses.

Embora o seguimento pós-operatório com exames de imagem seja recomendado desde os primórdios do reparo endovascular dos AAAs, esse seguimento dificilmente é obtido da forma planejada, com falhas de seguimento em mais de 60% dos casos^177,178^. A taxa de rotura tardia após o tratamento endovascular pode chegar a 5% em 8 anos, e é importante salientar que essa incidência está mais relacionada a uma seleção inadequada dos casos, principalmente nos casos em que há anatomia desfavorável e utilização dos dispositivos fora das IFUs. Esses fatores parecem ser mais relevantes que propriamente uma falha intrínseca da técnica endovascular ou do material utilizado^13^.

A redução do saco aneurismático durante o acompanhamento é um importante indicativo da exclusão bem-sucedida do aneurisma e demonstrou ser um preditor de baixo risco de complicações durante os primeiros 5 anos pós-operatórios^179^. Por outro lado, nenhum estudo foi capaz de demonstrar aumento de sobrevida ou menor taxa de roturas em pacientes submetidos a um protocolo rígido de acompanhamento^179^.

Embora não exista consenso entre os diferentes serviços e publicações, as recomendações de seguimento no pós-operatório do tratamento endovascular estão baseadas em exame de angiotomografia 30 dias após o procedimento inicial e, na ausência de vazamentos e com áreas de selamento satisfatórias, o paciente deve ser submetido anualmente a exame de angiotomografia. Caso haja *endoleak* ou aumento do saco aneurismático, o exame deve ser realizado em 6 meses^180,181^.

Alguns autores demonstraram segurança no acompanhamento com ultrassonografia, seja de forma isolada ou após um exame inicial com angiotomografia que demonstre zonas de selamento adequadas e ausência de *endoleak* do tipo I ou III^180,182^. A ultrassonografia com contraste é um exame ainda pouco utilizado, mas que apresenta sensibilidade superior à da angiotomografia na identificação de *endoleaks*. É um exame mais barato, não utiliza radiação e não apresenta toxicidade renal. As desvantagens desse exame são semelhantes ao ultrassom convencional, sendo dependente da experiência do examinador e com limitações relacionadas à obesidade e presença de gases abdominais^183^. Nos pacientes submetidos à cirurgia aberta o exame de imagem deve ser realizado a cada 5 anos, preferencialmente com angiotomografia^184^.

Na Tabela 10, encontram-se as recomendações referentes ao seguimento pós-operatório dos AAAs tratados por RA ou EVAR.

**Tabela 10:** Recomendações referentes ao seguimento pós-operatório dos aneurismas abdominais tratados de forma convencional ou endovascular

Recomendação	Nível de evidência
No seguimento após tratamento endovascular do aneurisma da aorta abdominal está recomendado exame de angiotomografia 30 dias após a cirurgia.	I
Após angiotomografia inicial, caso não haja *endoleak* ou aumento do aneurisma, o próximo exame deve ser realizado em 12 meses. Esse exame pode ser angiotomografia ou ultrassom Doppler, de acordo com as características do serviço e do paciente.	I
Caso a angiotomografia inicial de 30 dias demonstre *endoleak* tipo II, um exame de imagem deve ser repetido em 6 meses. Esse exame pode ser angiotomografia ou ultrassom Doppler, de acordo com as características do serviço e do paciente.	IIb
Caso o exame de imagem após 12 meses demonstre ausência de *endoleak*, ausência de aumento do saco aneurismático e áreas de selamento adequadas, um exame de imagem deve ser realizado anualmente ou em intervalo mais longo, não maior que 5 anos.	IIb
Tomografia da aorta e ilíacas deve ser realizada a cada 5 anos em pacientes submetidos ao tratamento aberto do aneurisma da aorta abdominal.	IIb

## COMPLICAÇÕES PÓS-OPERATÓRIAS

### Infecção de prótese

A infecção de prótese aórtica é uma infecção em uma prótese primária. Tal conceito inclui tanto próteses utilizadas no RA quanto endopróteses utilizadas no reparo endovascular. Trata-se de uma complicação rara, com incidência reportada na literatura de até 1%^185,186^, e, em sua maioria, no primeiro ano de pós-operatório. Dados recentes da literatura não apontam diferença estatística significativa entre sua incidência em pacientes submetidos ao RA em relação ao reparo endovascular^187^. As principais fontes de infecção são: contaminação no momento do implante; fístula aorto-entérica (FAE) ou erosão para órgão adjacente (trato gastrointestinal ou via aérea); ou, mais raramente, via disseminação hematogênica^188^.

Infecções precoces (até 3 meses da intervenção) frequentemente estão associadas a quadros de febre, dor abdominal ou lombar e leucocitose, enquanto nas infecções tardias (após 3 meses da intervenção) os sintomas apresentam-se de maneira insidiosa, com fadiga, mal-estar, perda ponderal, com ou sem febre^185^. Pacientes com FAE apresentam com frequência sintomas mais graves, como sangramentos, sepse e choque hemorrágico^189^.

Os exames complementares iniciais para diagnóstico incluem exames laboratoriais, hemocultura e exame de imagem, preferencialmente angiotomografia computadorizada. Em casos de suspeita de FAE ou hemorragia, é indicada a realização de endoscopia e/ou colonoscopia. Critérios diagnósticos propostos por Lyons et al.^190^ são de grande utilidade para casos suspeitos de infecção de prótese aórtica. Os achados radiológicos incluem líquido ao redor da prótese ≥ 3 meses após implante, gás ao redor da prótese ≥ 7 meses após implante ou aumento da quantidade de gás em TC seriadas, abcesso, alterações inflamatórias, rápido crescimento do aneurisma ou pseudoaneurisma^189,190^.

O tratamento conservador é o tratamento de exceção, e inclui drenagem percutânea e antibioticoterapia prolongada. Pode ser iniciado para pacientes em preparo para o tratamento cirúrgico ou para pacientes de alto risco cirúrgico como medida paliativa^185,191,192^.

Para pacientes com risco cirúrgico aceitável e infecção de prótese aórtica, o tratamento preconizado é a excisão completa da prótese/endoprótese e de tecidos infectados como tratamento definitivo^185,193^. A escolha do tipo de reconstrução arterial deve ser realizada de maneira individual, levando-se em consideração o tipo de prótese infectada, experiência do cirurgião, *status* do paciente e materiais disponíveis.

As técnicas disponíveis para reconstrução arterial incluem *bypass* extra-anatômico ou *in situ* com enxerto autólogo, enxertos criopreservados (não disponíveis no território nacional) ou enxerto sintético embebido em rifampicina. Não há evidência de superioridade entre os enxertos, e o uso de qualquer uma destas opções é aceitável em paciente estáveis sem extensa infecção por microorganismos multirresistentes^185,194^.

Para pacientes com abcesso extenso periprótese ou infecção por bactérias multiresistentes, como *S. aureus* resistentes à meticilina e *Pseudomonas,* as técnicas de *bypass* extra-anatômico ou reconstrução *in situ* com veia femoral ou aloenxerto podem oferecer maior tempo livre de reinfecção^195^.

Reconstruções com enxerto autólogo *in situ* possuem as menores taxas de reinfecção; no entanto, estão associados a maior tempo operatório, incompatibilidade de tamanho com artéria e complicações venosas em membros inferiores^195,196^. Pacientes instáveis que necessitam de rápido controle vascular proximal e reconstrução arterial, devem ser manejados preferencialmente com aloenxerto (se prontamente disponível) ou prótese embebida em rifampicina^189,195-197^.

O tratamento endovascular tem seu papel quando é necessário rápido controle de hemorragias, e pode aumentar sobrevida em pacientes com FAE quando realizado como terapia ponte até o tratamento definitivo. Em pacientes sem condições clínicas de serem submetidos à excisão do enxerto infectado, o reparo endovascular pode ser considerado como tratamento definitivo. Nesses casos, a antibioticoterapia perene deve ser considerada^185,189,198^.

A infecção de prótese aórtica apresenta alta taxa de mortalidade precoce, variando entre 15-22%^185^. Apesar do aumento progressivo do tratamento de aneurismas de aorta nos últimos anos, com o advento do reparo endovascular, é uma complicação rara e, portanto, as evidências científicas sobre seu tratamento ainda são limitadas.

O tratamento e seguimento desses pacientes deve ser realizado por uma equipe multidisciplinar, envolvendo infectologistas para determinar antibioticoterapia apropriada. O tratamento com antibiótico endovenoso é recomendado por um período de 6 semanas, seguido de manutenção com terapia oral por 3-6 meses, dependendo da extensão da infecção, microorganismo associado e tipo de reparo realizado^195,199,200^. A antibioticoterapia perene é recomendada em casos selecionados, como pacientes com infecção extensa, microorganismos resistentes, reconstruções *in situ* com prótese ou reparo endovascular sem ressecção completa do enxerto infectado^185,198,201,202^.

O seguimento deve ser realizado com exames de imagem e exames laboratoriais a cada 3-6 meses no primeiro ano, e de maneira contínua a cada 6-12 meses^185^.

### Endoleak

O EVAR associa-se a complicações que podem levar à falência da endoprótese e rotura do saco aneurismático ao longo do tempo, e, por isso, necessita de seguimento e monitorização^203-206^. Esta seção tem por objetivo abordar criticamente as principais complicações pós-EVAR relacionadas com vazamentos (*endoleaks*), suas implicações no seguimento, visando a reconhecê-las e tratá-las antes que culminem com reenchimento e pressurização do saco aneurismático^206,207^.

As complicações pós-EVAR ocorrem entre 16-33% dos pacientes^206,207^. Em estudo mais recente, observou-se 26% de complicações, sendo 39,4% identificadas durante o primeiro ano^206^. A mais temida dessas complicações é a rotura do aneurisma, podendo ocorrer como consequência de *endoleaks*, pressurização do saco aneurismático (endotensão), migração, deterioração das endopróteses ou degeneração do colo proximal por progressão da doença aneurismática^204,206^.

*Endoleak* significa a presença de fluxo no saco aneurismático fora da endoprótese após EVAR^208^ e ocorre em até 1/3 dos casos^205^, embora a prevalência dependa do tipo de endoprótese utilizada, bem como do exame de imagem realizado durante o seguimento^203,206,207^. Os *endoleaks* são classificados em primários/precoces (presentes no momento do reparo) ou secundários/tardios (detectados no seguimento pós-operatório com imagens de controle normais prévias^209^, assim como quanto à causa do fluxo periendoprótese. A presença de *endoleak* afeta o encolhimento do saco aneurismático ao longo do tempo devido à pressurização do saco aneurismático^205^. Aproximadamente metade dos vazamentos (principalmente do tipo II) se resolvem espontaneamente, sem necessidade de reintervenção^205^. A terapia anticoagulante pode aumentar o risco de desenvolvimento de *endoleaks* após o EVAR^210^. O reconhecimento precoce dessa complicação, antes de se tornar clinicamente relevante, possibilita o seu tratamento e previne situações mais graves. Dessa forma, torna-se necessário que o cirurgião vascular se familiarize com os exames de imagem disponíveis para o seguimento pós-EVAR para diagnosticar e melhor conduzir as eventuais complicações que possam surgir^211^.

Atualmente, cada vez mais, pacientes com critérios anatômicos desfavoráveis ao tratamento endovascular têm sido submetidos a essa modalidade terapêutica, podendo cursar com uma elevação da necessidade de procedimentos adjuvantes e um aumento das taxas de intervenção secundária, apesar do aprimoramento dos materiais utilizados. Dessa forma, espera-se que o cirurgião que ofereça o EVAR para seu paciente esteja familiarizado com tais complicações, seja capaz de utilizar adequadamente a propedêutica armada, classificar precisamente o tipo de vazamento e indicar o momento oportuno de uma intervenção secundária^212^.

### Endoleak tipo I

O *endoleak* tipo I é definido como selamento circunferencial inadequado nas áreas de fixação da prótese na parede aórtica, promovendo fluxo direto persistente no saco aneurismático^203,205,206^. É subdividido em tipo IA, quando há vazamento oriundo da fixação proximal (colo proximal); IB, quando há vazamento na região de fixação distal (eixo ilíaco); e IC, quando o vazamento ocorre por comunicação direta entre a aorta e a ilíaca após o implante de um *plug* oclusor na ilíaca durante a utilização de endoprótese monoilíaca^213^. Como há pressurização direta do saco aneurismático nesse tipo de vazamento, frequentemente cursando com progressão no crescimento do aneurisma e consequente risco elevado de rotura, o *endoleak* tipo I deve ser prontamente tratado, com o objetivo de excluir o aneurisma da circulação pressurizada. As opções endovasculares para o tratamento do *endoleak* tipo IA incluem dilatação por balão nos pontos de fixação da endoprótese com ou sem implante de stent ou fixação do tecido da endoprótese na parede aórtica com grampos endovasculares (endoâncoras), se não tiver havido migração e houver uma zona de selamento (colo proximal) apropriada para obter a vedação^214^. Mais comumente, há necessidade de extensão proximal da zona de selamento, sendo necessário implante de cuff proximal ou endoprótese fenestrada. No tipo IB, uma extensão para ilíaca distal normalmente é suficiente^214^. Contudo, caso ocorra a progressão da doença por degeneração do colo distal, acometendo a bifurcação ilíaca, deve-se ponderar entre estender para a artéria ilíaca externa ou utilizar uma endoprótese bifurcada de ilíaca. Se uma opção endovascular não estiver disponível em tempo razoável e o paciente estiver apto para correção aberta, recomenda-se conversão com resultados aceitáveis^215^.

### *Endoleak* tipo II

O *endoleak* tipo II é definido como o enchimento do saco aneurismático por fluxo sanguíneo retrógado, proveniente de ramos colaterais aórticos. As artérias mais comumente envolvidas nesse tipo de vazamento são a artéria mesentérica inferior e as artérias lombares^203,205,206^. Quando há expansão do saco aneurismático devido a uma suspeita de *endoleak* tipo II, exames de imagem adequados devem ser realizados para descartar outras causas, como vedação inadequada ou *endoleak* interno do tipo III (conexão, integridade do enxerto ou orifícios de sutura)^216^. É classificado em tipo IIa, quando apenas um ramo colateral está envolvido, e em tipo IIb, quando dois ou mais ramos fornecem fluxo para o saco aneurismático^217^.

São divididos em:

- Tipo II precoce, quando diagnosticado em menos de 30 dias pós-EVAR;
- Tipo II tardio, quando diagnosticado entre 30 dias-6 meses pós-EVAR;
- Tipo II persistente, quando se mantém por mais de 6 meses pós-EVAR.

Em metanálise com 2.367 pacientes com EVAR, 18% tiveram *endoleaks* precoces do tipo II que se resolveram espontaneamente, 5% mantiveram *endoleaks* persistentes do tipo II e 11% desenvolveram novo vazamento do tipo II durante o acompanhamento^212^. Aproximadamente metade dos pacientes com *endoleaks* persistentes ou tardios evoluiu com crescimento do saco, com taxa de reintervenção de 50% em 2 anos. Os fatores associados ao *endoleak* persistente ou recorrente do tipo II incluíram embolização com molas das artérias ilíacas internas, extensão distal da endoprótese, idade acima de 80 anos e fatores anatômicos como número de ramos laterais pérvios decorrentes do aneurisma, trombo no saco aneurismático e diâmetro das artérias lombares (> 0,2 cm) e mesentéricas inferiores (> 0,3 cm)^218,219^. A embolização pré-operatória do saco aneurismático em pacientes selecionados foi sugerida como uma técnica para reduzir o risco de desenvolvimento de *endoleak* tipo II^220,221^, mas o benefício de redução nas reintervenções ou diminuição da incidência de ruptura ainda é controverso^222^.

A evolução da maioria dos *endoleaks* do tipo II parece ser benigna, mas a ruptura do aneurisma pode ocorrer mesmo assim^223^. No entanto, em revisão sistemática baseada em estudos retrospectivos, menos de 1% dos *endoleaks* do tipo II resultou em ruptura, e a reintervenção foi indicada para correção de vazamento persistente com expansão do saco aneurismático^216,224^. Embora a maioria dessas rupturas pareça estar relacionada com expansão do saco aneurismático, também foram relatadas rupturas na ausência de expansão do aneurisma^217^. Alguns centros tratam *endoleaks* tipo II se houver expansão do saco aneurismático >1 cm, e outros, > 0,5 cm, sendo esse o limite inferior para detecção de expansão do saco aneurismático entre dois exames de imagem usando a mesma modalidade^212^.

A correção endovascular pode ser realizada através da embolização do saco aneurismático e/ou dos vasos de retroalimentação por via transarterial, translombar, transcaval ou *transealing* (entre o ramo ilíaco da endoprótese e parede da artéria ilíaca nativa), utilizando-se vários dispositivos^216,224^. O sucesso técnico é alcançado em 60-80% dos casos; no entanto, falta uma definição objetiva na indicação e conduta para esses casos, o que pode afetar a interpretação desses resultados^212^.

As opções de tratamento cirúrgico incluem a ligadura laparoscópica ou aberta dos ramos laterais que retroalimentam o saco aneurismático, sutura dos óstios desses por visão direta após a abertura do saco aneurismático ou retirada cirúrgica da endoprótese, com conversão para cirurgia convencional em casos de falha do tratamento endovascular^212,216,225^.

### *Endoleak* tipo III

O *endoleak* tipo III pode ser definido como vazamento secundário a alguma falência estrutural da endoprótese. É classificado em tipo IIIA, se causado por desconexão de módulos, e tipo IIIB, se por defeito de fabricação, sendo esse último subdividido naqueles com orifícios maiores ou menores que 0,2 cm. Esses *endoleaks* podem ocorrer devido ao mau posicionamento das endopróteses com sobreposição inadequada, migração da endoprótese proximal ou distal ou fadiga do material^203,205,206^. A incidência é de 2,1% em até 4 anos após o EVAR, sendo 56% tipo IIIA e 44% tipo IIIB^226^. No entanto, quando são utilizadas endopróteses mais modernas, a incidência pode ser reduzida para 1%.

Assim como nos *endoleaks* do tipo I, nesse vazamento há pressurização direta do saco aneurismático, com risco subsequente de ruptura^223^. Portanto, a correção endovascular imediata é recomendada, sendo as opções mais utilizadas o implante de extensão ilíaca, cuff coaxial no sítio do vazamento, implante de nova endoprótese bifurcada ou conversão para endoprótese monoilíaca, seguida por revascularização do membro contralateral por ponte fêmoro-femoral cruzada. A conversão para cirurgia aberta é necessária apenas se as medidas endovasculares descritas não tiverem sido capazes de controlar o vazamento^226,227^.

### *Endoleak* tipo IV

O *endoleak* tipo IV é muito raro na prática atual e relaciona-se com a porosidade do tecido de revestimento da endoprótese, podendo estar relacionado ao uso de terapia anticoagulante ou antiplaquetária. O vazamento se dá através do tecido intacto, e na extensa maioria dos casos é resolvido nos primeiros 30 dias de pós-operatório, sendo considerado de natureza benigna^203,205,206^. De acordo com uma revisão de rupturas pós-EVAR relatadas na literatura até 2008, nenhum caso de ruptura por vazamento tipo IV foi encontrado^223^. O vazamento tipo IV é raro na maioria dos dispositivos modernos e não requer reintervenção.

### *Endoleak* tipo V ou endotensão

O *endoleak* tipo V, também conhecido por endotensão, significa a presença de expansão do saco sem qualquer *endoleak* identificável. A incidência está entre 1,5-5% e todos os outros tipos de *endoleaks* precisam ser excluídos antes de um diagnóstico definitivo^203,205,206^. Vários mecanismos possíveis para endotensão foram sugeridos, incluindo aumento da permeabilidade das endopróteses, resultando na transmissão direta de pressão através das endopróteses para a parede aórtica, ou o uso de endopróteses de primeira geração de politetrafluoretileno, que poderia dificultar a organização do trombo e a fibrinólise no saco aneurismático^228^. No entanto, dada a definição, é possível que os casos classificados como endotensão sejam devido a um *endoleak* que não pode ser definido com as modalidades de imagem atuais^228^. O tratamento é indicado quando há crescimento significativo do saco aneurismático (>1 cm) e consiste no realinhamento ou retirada da endoprótese por cirurgia aberta^229^.

### Migração

Conceitualmente, a migração da endoprótese é definida como movimento da endoprótese > 1 cm em comparação com pontos anatômicos fixos de referência, verificados na linha central de reconstruções de imagens tomográficas, ou qualquer migração resultando em sintomas ou reintervenção^230^. A migração da endoprótese já foi um evento comum, e a maioria dos estudos sobre fatores de risco para migração do dispositivo proximal foi realizada a partir de séries de casos com endopróteses de primeira geração, mas no desenvolvimento de fixação ativa supra ou infrarrenal em endopróteses mais modernas, a prevalência foi reduzida^231,232^.

A migração pode resultar em *endoleak* tipo I, separação dos componentes da endoprótese, *kinking* e oclusão de ramos. Os fatores que podem contribuir para migração proximal incluem fixação proximal curta, colo angulado, aneurisma de grandes dimensões, tipo de endoprótese^230,233,234^ e superdimensionamento da endoprótese > 30%, que, apesar de controverso, mostra evidências de que também pode contribuir com a migração^235,236^. A progressão da doença com dilatação do colo proximal pode ser causa de migração e está relacionada com diâmetro inicial do mesmo^237^.

A migração também pode ocorrer devido a alterações na morfologia do aneurisma ou encolhimento do saco aneurismático após o EVAR. Um comprimento de fixação ilíaca (colo distal) de pelo menos > 2 cm ou preferencialmente até a bifurcação ilíaca reduz o risco de migração da endoprótese^238,239^.

Na Tabela 11, encontram-se resumidas as recomendações referentes ao tratamento dos *endoleaks.*

**Tabela 11:** Recomendações referentes aos tratamentos de *endoleaks* após correção endovascular de aneurisma da aorta abdominal

Recomendação	Nível de evidência
Em pacientes com *endoleak* tipo I após correção endovascular de aneurisma de aorta abdominal é recomendada a reintervenção precoce para obter o selamento e exclusão do aneurisma, preferencialmente por técnica endovascular.	I
Recomenda-se a preservação de pelo menos uma das artérias hipogástricas caso haja acometimento da bifurcação ilíaca.	I
A reintervenção para tratamento de *endoleak* tipo II após reparo endovascular de aneurisma de aorta abdominal deve ser considerada na presença de crescimento significativo do aneurisma, devendo ser feita preferencialmente por via endovascular.	IIa
A expansão do diâmetro do saco aneurismático > 1 cm verificada durante o seguimento pós-correção endovascular do aneurisma da aorta abdominal, utilizando a mesma modalidade de imagem e método de medição, pode ser considerado um critério razoável de detecção de crescimento significativo nos casos de endoleak tipo II.	IIb
Em pacientes com *endoleak* tipo III após correção endovascular de aneurisma de aorta abdominal, a reintervenção é recomendada, preferencialmente por técnica endovascular.	IIa
Crescimento significativo do saco aneurismático após o reparo endovascular do aneurisma da aorta abdominal sem *endoleak* identificável nos exames de imagem padrão deve considerar avaliação diagnóstica adicional ou eventualmente o realinhamento ou explante da endoprótese.	IIa

## ANEURISMA DE AORTA ABDOMINAL ROTO

O aneurisma de aorta roto é uma das condições mais dramáticas no paciente de emergência. Pode ser letal na ampla maioria dos casos, senão todos, quando não tratado. A taxa de rotura tem como principais fatores o tamanho, a morfologia e a velocidade de crescimento^240^. No Brasil, estima-se que de 2000-2016, 38.000 pessoas morreram em decorrência direta da rotura, o que representa 55% de todas as mortes relacionadas a aneurisma de aorta^4^. Historicamente, a mortalidade do aneurisma roto chegava a 50% no intra-hospitalar, mas a difusão do tratamento endovascular causou uma queda de mortalidade, que atualmente é da ordem de 20-30%^240-243^.

A tríade clássica de sintomas do AAA roto inclui hipotensão, dor abdominal e massa pulsátil. Outras manifestações, contudo, podem ocorrer, como dor nas costas, na virilha, e no caso de perfuração de uma víscera pode haver sangramento do trato gastrointestinal, com consequente hematêmese ou melena. Existe uma situação de rotura peculiar que é a rotura para dentro da veia cava inferior, que se manifesta como fístula aortocaval, cujo sinal frequente é um frêmito abdominal, e pode cursar com insuficiência cardíaca aguda e grave e embolia pulmonar paradoxal^6^.

Anatomicamente, o local da rotura também está associado a fatores prognósticos. A rotura do AAA é a mais comum, e existe diferença se ela ocorre na parede anterior do aneurisma ou posterior. Na parede anterior, comumente perfura também o retroperitôneo, ganhando a cavidade peritoneal, o que dificulta o tamponamento do sangramento, e muitos pacientes nessa situação evoluem para óbito sem receber atendimento hospitalar. O aneurisma roto tamponado é frequentemente decorrente de rotura de parede posterior, e a coluna vertebral e o par de músculos íleopsoas ajudam na contenção do sangramento, e o paciente tem maior chance estatística de chegar vivo ao atendimento hospitalar.

O diagnóstico certamente começa na suspeita clínica, e possivelmente mais fácil quando o paciente sabe o diagnóstico de AAA previamente. O desafio talvez seja manter um alto grau de suspeição para aqueles doentes que se encontram hipotensos, apresentam risco de vida, mas a causa não é tão aparente.

A criação de protocolos e volume e experiência do serviço estão associadas a melhores desfechos e maiores chances de sobrevida no mundo todo^7,244-246^. Tanto a parte diagnóstica quanto de encaminhamento ao tratamento necessita de fluxo previamente estabelecido com os setores hospitalares.

O paciente com aneurisma roto deve receber seu primeiro atendimento já na sala de emergência. Nesse ambiente, deve-se providenciar acesso periférico calibroso e já a primeira coleta de laboratório. A reposição volêmica deve ser feita tendo como meta o conceito de hipotensão permissiva^7,244^, que significa manter o paciente consciente com pressão sistólica entre 70-90 mmHg. Essa estabilização é recomendada caso o paciente deva ser transferido para um centro com um time de aorta e preparo para ambas as modalidades terapêuticas, endovascular ou convencional.

O ultrassom de abdome e a radiografia tórax costumam ser exames amplamente disponíveis na sala de emergência e podem revelar, no caso do ultrassom, líquido livre abdominal ou no recesso costal, e no caso da radiografia de tórax poderá ser visto velamento de um hemitórax, ou aumento da área cardíaca, caso seja uma rotura torácica.

Esses exames, contudo, não excluem a rotura porque o sangramento pode estar contido, e o exame diagnóstico preferencial é a angiotomografia de aorta, que deve incluir aquisições desde a aorta ascendente e o arco até as artérias femorais comuns. A técnica adequada é fundamental para que o planejamento cirúrgico possa acontecer.

A angiotomografia pode demonstrar sinais claros ou indiretos da rotura da aorta abdominal. São eles o borramento do músculo psoas, descontinuidade da parede aórtica, extravasamento de contraste, sinal do crescente ou fissura no trombo mural, hematoma intraperitoneal ou retroperitoneal^247^.

O tratamento do aneurisma roto historicamente apresentava altas taxas de mortalidade, e o advento da cirurgia endovascular causou uma diminuição significativa dessa taxa. Após 20 anos de reparo endovascular consistente, há robustas evidências, com estudos randomizados e metanálises, que frente a uma rotura de aneurisma da aorta o tratamento endovascular é seguramente melhor que o aberto^6,7,240,242,243,248,249^.

Esse melhor resultado se traduz não apenas em menor mortalidade em 30 dias, mas também no médio e longo prazo, além de recuperação com menor taxa de complicações, como estomias e amputações, e melhor qualidade de vida^248^. Dessa forma, capacitar a equipe, disponibilizar material e sala cirúrgica preparada para o reparo aberto ou endovascular trazem impacto direto nas chances de o paciente sobreviver a uma rotura^244^.

A principal limitação para oferecer o EVAR em um caso de AAA roto é a anatomia, e especificamente o diâmetro e o comprimento do colo proximal onde a endoprótese vai ser fixada. Portanto, na ausência dessas condições ou de material e equipe para o tratamento endovascular, a cirurgia aberta se impõe, e o centro de referência deve estar preparado para ambas as opções.

A hipotensão permissiva e o preparo do paciente para anestesia com a equipe posicionada para intervenção podem diminuir a chance de ocorrer o destamponamento da rotura. Por isso, a coordenação dos passos com a equipe de anestesia é fundamental. Reposição volêmica adequada e de hemoderivados faz parte de uma terapia eficaz.

Para ambos os reparos, no momento do acesso pode ser necessário o clampeamento supracelíaco da aorta em caso de choque hipovolêmico grave. Esse procedimento pode ser feito por via aberta, através de laparotomia mediana, dissecção romba da curvatura menor do estômago e clampeamento da aorta contra a coluna. É possível também fazer por via endovascular, e até mesmo sob anestesia local, através de acesso femoral por dissecção, colocação de introdutor 12FR e fio guia ipsilateral para passagem de balão de oclusão da aorta, que pode ser insuflado na altura da 12a. vértebra torácica^244^. É importante lembrar que o paciente hemodinamicamente instável apresenta altas taxas de mortalidade, e a pressão sistólica abaixo de 70 mmHg é um marcador prognóstico de óbito independente. Finalmente, é necessário dizer que, nesses casos, submeter o paciente instável a um reparo convencional é quase em sua totalidade fatal, e reparo endovascular se justifica ainda mais nesta condição^7^.

Existe uma vantagem adicional do EVAR em relação ao tratamento convencional na rotura que é a possibilidade de submeter o paciente à anestesia local, e somente usar a sedação se necessário^244^ – e a evidência atual aponta para melhores desfechos quando o paciente é submetido a tratamento com anestesia local se a hemodinâmica permitir^250-252^. Além disso, não intubar o paciente na situação de rotura está associado a melhores chances de sobreviver^240^.

A escolha da endoprótese entre bifurcada e mono-ilíaca certamente deve envolver a experiência e preferência do cirurgião, uma vez que obter rapidamente o controle hemostático é crucial. O tempo de cateterização do ramo contralateral não deve ser prolongado, mas é certo que evitar um enxerto femoro-femoral cruzado também traz vantagens para o paciente. De modo geral, priorizar o uso de endoprótese bifurcada parece razoável conforme a experiência dos grupos avança^6^.

Na Tabela 12, é possível encontrar as recomendações referentes ao tratamento dos AAAs rotos.

**Tabela 12:** Recomendações referentes ao tratamento dos aneurismas abdominais de aorta rotos

Recomendação	Nível de evidência
Frente a um aneurisma de aorta abdominal roto recomenda-se o reparo endovascular.	I
Caso o hospital não tenha condições de oferecer reparo endovascular, é aceitável transferir o paciente para centro especializado, desde que a condição clínica e hemodinâmica do paciente permitam.	IIa
Reposição volêmica deve ser feita na medida a deixar o paciente consciente e com pressão sistólica entre 70-90 mmHg.	I
No paciente que chega ao hospital, considerar estabilização e aquisição de imagem de angiotomografia toracoabdominal para ajudar no planejamento cirúrgico.	IIb
Dar preferência ao uso de endoprótese bifurcada desde que haja condições anatômicas para tal.	IIb

## ANEURISMA INFLAMATÓRIO

Os aneurismas aórticos inflamatórios são caracterizados por uma camada espessa de tecido inflamatório que frequentemente cursa com fibrose periaórtica, retroperitoneal e aderência de órgãos e tecidos adjacentes. O infiltrado inflamatório exuberante na camada adventícia está entre as principais diferenças para um aneurisma degenerativo aterosclerótico^253^. Essa condição o torna de difícil abordagem cirúrgica quando considerado o RA, e potencialmente letal^254^. A incidência reportada na literatura varia de 5-18%^254,255^.

Do ponto de vista fisiopatológico, os mecanismos que levam a essa inflamação ainda não são completamente compreendidos. Sintomas clínicos, como dor lombar, febre, emagrecimento e falta de apetite, podem estar presentes. Sexo masculino, tabagismo e fatores genéticos estão entre os principais fatores de risco identificados até o momento^253,255^. Ao contrário de outras doenças inflamatórias ou reumatológicas, mais frequentemente o achado da inflamação da aorta é isolado e não acomete outras artérias^254^.

Os exames laboratoriais podem estar alterados, incluindo leucócitos e marcadores inflamatórios como proteína C reativa. A angiotomografia de aorta vai revelar alguns sinais tais como o espessamento periaórtico, podendo também acometer o retroperitôneo, e é um exame com boa sensibilidade para diagnóstico^253^. A ultrassonografia de abdome, apesar de não trazer uma imagem tão nítida quando comparada a angiotomografia, consegue identificar muitas vezes um halo hiperecogênico envolvendo a parede aórtica. Tomografia por emissão de pósitrons também pode revelar inflamação periaórtica^254^.

O RA pode ser desafiador e eventualmente letal devido às aderências aos órgãos adjacentes, como veia cava inferior, ureteres e intestino delgado, o que pode ser causa de enterectomias^253,256^. Dado esse histórico, é comum ver cada vez mais intervenções endovasculares para o tratamento dos aneurismas inflamatórios. É importante frisar, contudo, que após análise dos dados de literatura, faltam estudos randomizados e acompanhamento de longo prazo para demonstrar sua eficácia e segurança^254,256^.

## ANEURISMA EM MULHERES

Os AAAs são menos comuns em mulheres do que em homens, numa proporção que varia de 1:4 a 1:9 na literatura. Não existem estudos clínicos randomizados que abordem o AAA especificamente em mulheres. Todavia, existem muitos estudos científicos de valor sobre esse importante tema. A prevalência dos AAAs na população de mais de 60 anos é de 0,7% e aumenta rapidamente com o envelhecimento^103^. A mortalidade operatória aumenta com a idade e as mulheres apresentam AAA clinicamente relevantes com idade mais avançada que os homens. A morfologia dos AAAs difere muito entre os sexos: homens tendem a ter aortas, ilíacas e femorais mais calibrosas do que as mulheres. As mulheres são mais idosas e com AAAs menores por ocasião do tratamento^19,36,103,257^.

Os estudos randomizados EVAR 1, DREAM, OVER e ACE mostraram uma significativa redução de mortalidade entre cirurgia aberta e endovascular e sobrevida de longo prazo similar^212^. Esse benefício não se estendeu claramente a ambos os sexos. Mulheres tiveram menos vantagens, mas como o número dessas nos estudos foi pequeno, de 0,6-9%, não é possível uma análise estatística precisa.

Várias teorias foram propostas para explicar as diferenças entre homens e mulheres portadores de AAA, mas conclusões definitivas ainda não existem. Alguns trabalhos com dados estatísticos merecem ser destacados: Ulug et al*.* ^103^ *,* em metanálise sistemática de nove estudos realizados entre 2005-2016, com 52.018 homens e 11.076 mulheres, encontrou uma mortalidade de 30 dias com EVAR, em homens, de 1,4%, e em mulheres, de 2,3%. Na cirurgia aberta os números foram piores: homens totalizaram 2,8% e mulheres, 5,4%. Sidloff et al.^258^, em análise do UK National Vascular Registry (Reino Unido, 2010-2014), de 23.245 pacientes, dos quais 13% mulheres, encontrou uma mortalidade de 30 dias após EVAR em 0,7% de homens, e em 1,8% de mulheres. Na cirurgia aberta, os números de mortalidade foram piores: homens, 4%, e mulheres, 6,9%. Deery et al.^259^, em análise do American College of Surgeons National Surgical Improvement Program (2011-2014), encontraram uma mortalidade de 30 dias em homens, 1,2%, e mulheres, 3,2%, quando tratados com EVAR. Na cirurgia aberta, os números foram ainda piores: homens, 4%, e mulheres, 8%. Erben et al.^260^ relataram uma taxa de complicações e reintervenções maior em mulheres, sobretudo decorrentes de trombose arterial dos membros inferiores.

Embora a mortalidade do tratamento do AAA tenha diminuído exponencialmente na última década, a mortalidade em mulheres, sobretudo na cirurgia aberta, permanece excessivamente elevada. Wanhainen et al.^6^, nas Diretrizes que coordenaram, encontraram em mulheres uma mortalidade de 6,9% no tratamento aberto de AAA contra 1,8% no tratamento com método endoluminal. Embora sem importância estatística, em nível estatisticamente significativo persistem taxas maiores de complicações e de mortalidade em mulheres. Trabalhos recentes, como os de Tumer et al., Corsi et al., Ilyas et al. e Tedjawira et al. continuam mostrando mortalidade mais alta em mulheres^261-264^.

As razões são multifatoriais: mesmo sendo menos afetadas, as mulheres apresentam AAA em idade mais avançada, com mais comorbidades associadas, geralmente subdiagnosticadas. De relevância especial é que se apresentam com idade mais avançada, têm mais doença obstrutiva arterial associada, o que gera uma anatomia desafiadora para tratamento endovascular. Por essas razões, as mulheres têm maiores taxas de complicações e de mortalidade pós-operatória, tanto na cirurgia aberta como com o tratamento endovascular. Em suma, têm um pior prognóstico. Para maior segurança prognóstica, devem ter seus AAAs controlados com maior frequência a partir de 4 cm de diâmetro^36,103,257–265^

Em mulheres, o rompimento de AAAs ocorre em diâmetros menores do que em homens. Solberg et al.^266^ demonstrou que os AAAs crescem mais rápido em mulheres do que em homens. Estima-se que no tocante ao risco de rotura, um AAA de 4,5 cm de diâmetro em mulheres tem o mesmo risco de romper que um de 5,5 cm em homens. Com um diâmetro de 5 cm, a mulher tem risco de rotura de 1/30 casos – 3,3%. Também se sabe que com diâmetros idênticos o risco de rotura no sexo feminino é 4 vezes maior do que nos homens^19,36,257,265^. As razões também podem ser multifatoriais, mas certamente a maior delas é que, em valores absolutos, os diâmetros das artérias femininas são cerca de 1/3 menores do que nos homens, ou seja, o diâmetro dos AAAs é proporcionalmente muito maior nas mulheres, o que facilitaria seu rompimento. Também não podemos comparar os dados anatômicos da população anglo-saxônica, com a qual a maioria dos estudos populacionais sobre AAA foi produzida, com a brasileira, de porte muito menor, sobretudo das mulheres. Esses dados foram muito bem analisados na população asiática, de porte semelhante à nossa^267^. Assim, nos últimos anos, tem havido muitas publicações que indicam o tratamento do AAA em mulheres a partir de 4,5 cm de diâmetro, sobretudo não deixando ultrapassar 5 cm, em casos eletivos^252,254,257-263^. O risco cirúrgico do tratamento endovascular em vários estudos é de menos de 2%. Assim, é lógico indicar esse tratamento efetivo para o AAA em mulheres, de forma profilática, assim que o diâmetro ultrapassar 4,5 cm.

É sabido que comumente mulheres têm morfologia aortoilíaca desfavorável ao EVAR – colos curtos e angulados e artérias ilíacas e femorais menores, muitas vezes incompatíveis com o diâmetro dos sistemas introdutores das endopróteses. Sweet et al^264^. relataram que 70% dos homens e somente 40% das mulheres tinham anatomia compatível com as instruções de uso das endopróteses disponíveis em 2011. Nas décadas anteriores, essa realidade era ainda pior. Até o presente momento, não há endoprótese desenhada especificamente para mulheres, mas recentemente os desafios anatômicos da anatomia feminina passaram a ser objeto de estudo específico, com desenvolvimento de dispositivos que pudessem se adequar e assim expandir as indicações do EVAR em mulheres, inclusive reduzindo as taxas de complicações. Dados recentes da literatura sugerem que os maus resultados históricos possam estar relacionados a problemas técnicos e não ao sexo propriamente. Foram desenvolvidos três sistemas de endopróteses de ultrabaixo perfil: Incraft^®^, Ovation^®^ e Altura^®^. Outros estão em desenvolvimento. Estudos de longo prazo estão disponíveis com os dois primeiros sistemas e se comparam favoravelmente aos resultados históricos do tratamento do AAA em mulheres^265-267^.

Mulheres com AAA são tratadas em idade mais avançada, com mais comorbidades e com aneurismas proporcionalmente maiores. Frequentemente tem anatomia hostil ao EVAR, com aortas, ilíacas e femorais de pequeno calibre e com doença aterotrombótica mais avançada. São situações que demandam dispositivos específicos para suplantar esses desafios. Nas mulheres, endopróteses de baixo perfil e alta flexibilidade tem apresentado melhor resultado em relação ao sucesso técnico e mortalidade, reduzindo as complicações relacionadas aos procedimentos.

Aperfeiçoamento dos materiais, redução de perfil sem perda de resistência e acurácia do implante devem diminuir a estreita referência de resultados entre mulheres e homens. Casuísticas maiores e multicêntricas com os novos dispositivos e comprovação de sua eficácia e durabilidade em longo prazo são necessários para um posicionamento definitivo nesse tema.

Na Tabela 13, encontram-se as recomendações referentes ao tratamento dos AAAs em mulheres.

**Tabela 13:** Recomendações referentes ao tratamento dos aneurismas aórticos abdominais em mulheres

Recomendação	Nível de evidência
Mulheres com aneurisma da aorta abdominal que atinjam o diâmetro aórtico de 4 cm devem ser submetidas a controle de seus aneurismas semestralmente.	IIa
Recomenda-se o tratamento dos aneurismas da aorta abdominal eletivos em mulheres com diâmetros aórticos a partir de 5 cm.	IIa
Para o tratamento endovascular de aneurisma da aorta abdominal em mulheres deve-se buscar um dispositivo com dimensões dos sistemas de entrega que sejam compatíveis com a anatomia da paciente. Endopróteses de baixo perfil devem ser consideradas se necessário.	IIb
